# P2MAT: A machine learning (ML) driven software for Property Prediction of MATerial

**DOI:** 10.1186/s13321-026-01247-w

**Published:** 2026-07-03

**Authors:** Methun Kamruzzaman, Alexander Landera, Nalini Menon, Mark D. Allendorf, Kunal Poorey

**Affiliations:** 1 Applied Biosciences and Engineering, Sandia National Laboratories, 7011 East Ave, Livermore, CA 94550 USA; 2 Material Characterization and Performance, Sandia National Laboratories, 7011 East Ave, Livermore, CA 94550 USA; 3 Material Lifecycle management, Sandia National Laboratories, 7011 East Ave, Livermore, CA 94550 USA; 4 (Former) Materials Research, Sandia National Laboratories, 7011 East Ave, Livermore, CA 94550 USA; 5 Honorary Sr. Visiting Scholar, Washington University in St. Louis, 1 Brookings Drive, St. Louis, MO 63130 USA; 6 (Former) Applied Biosciences and Engineering, Sandia National Laboratories, 7011 East Ave, Livermore, CA 94550 USA; 7 Department of Computer Science and Data Science, Meharry Medical College, 1005 Dr DB Todd Jr Blvd, Nashville, TN 37208 USA

**Keywords:** QSPR modelling, Property prediction, Melting point, Boiling point, Machine learning, Linear regression, Ensemble, Deep learning, Explainable machine learning

## Abstract

Accurate prediction of melting points for pure molecules remains a significant challenge in predictive chemistry, with implications across various scientific fields, including materials science, drug discovery, and separations chemistry. Traditional methods, such as group contribution (GC) techniques, have shown limited success due to the complex relationship between molecular structure and melting point. In this study, we present a data-driven machine learning (ML) approach to predict the melting points of organic compounds, leveraging both 2D and 3D molecular descriptors. Our results indicate that ML models can significantly improve melting-point predictions, providing a robust tool for the scientific community.

**Scientific contribution**

Our detailed analysis on melting point prediction, along with SHAP explainability, reveals the top influencing features for the prediction. The P2MAT application we developed as part of this study can predict both melting and boiling points from a SMILES string. P2MAT is available as an easy-to-install, user-friendly GUI for maximum outreach to the scientific community. Our benchmark analysis demonstrates the excellence of our method for predicting melting points.

**Supplementary Information:**

The online version contains supplementary material available at https://doi.org/10.1186/s13321-026-01247-w.

## Introduction

An important and long-standing goal in predictive chemistry is the development of an accurate melting point model that is generally applicable to a wide range of molecules. Many decades of work have been invested in this endeavor, but thus far, none are sufficiently accurate to make useful and confident predictions for compounds without a measured melting point. Indeed, an improved understanding of melting point would be useful across large areas of science, including, but not limited to, materials for energy applications [1–3], drug discovery [4, 5], organic hydrogen getters [6, 7], ionic liquids [8, 9], and separations chemistry [10, 11] as well as the prediction of properties such as viscosity [12]. Accurate predictions of melting points are difficult because large differences in melting points can arise from small changes to molecular structure. Existing melting point models fall broadly into two categories: those that leverage group contribution (GC) theory and those that use machine learning (ML) techniques. Krzyzaniak et al. [13] used the solid–liquid phase transition equation, $T_m = \frac{\Delta H_m}{\Delta S_m}$, to develop a melting point model. The enthalpy of melting ($\Delta H_m$) was estimated using GC methods, and the entropy of melting ($\Delta S_m$) was estimated using an equation that depends on the rotational symmetry number and the molecular flexibility number of the target molecule. Using a database of 596 melting points containing only aliphatic non-hydrogen-bonding molecules, their method was found to have a root mean square error (RMSE) of $34.3\,^\circ$C. Subsequent papers from this group have suggested that the largest source of error comes from the estimate of $\Delta S_m$. The model was later expanded to include aromatic and aliphatic rings using a modified expression for the molecular flexibility number, yielding a comparable average absolute error (AAE) of $33.2\,^\circ$C [14]. Estimating $\Delta S_m$ is challenging and limits the utility of methods that make use of the phase transition equation. Marrero and Gani [15] developed a different GC method that accounts for the chemical environment around a chemical group via the use of second-order and third-order group contributions. Second and third-order group contributions are corrections to the original, first-order contribution. Special emphasis was given to improving the prediction of cyclic groups (both aliphatic and aromatic). When both second and third-order groups were used, the AAE was reduced to $20.22\,^\circ$C (down from $24.9\,^\circ$C when only first-order corrections were used). ML methods that incorporate molecular descriptors can serve as an alternative to GC-based methods. For example [16], used a series of ML methods incorporating 2D and 3D features. 2D features are chemical descriptors that encode topological information, whereas 3D features encode 3-dimensional shape and functionality information. Several approaches were used, in which different sets of databases, different ML methods, and different sets of features were used. These approaches achieved an MAE of 25–$29\,^\circ$C. [17] used the Cambridge Crystallographic Data Center (CCDC) database, which contains 58, 810 unique crystal structures with associated melting points (Zhu 2023). Some of these crystal structures are duplicates, but are considered unique by the authors because they are different enough. The authors argue that because their goal is to generate 3D descriptors from the crystal structures, these duplicates are worth keeping in the dataset. The authors went on to train their melting point model by using Deep learning [18], XGBoost [19], and Random forest methods [20], using 2D, 3D, and 2D + 3D descriptors. The best-performing model was trained using XGBoost with only 2D descriptors. This model achieved an MAE of $28.1\,^\circ$C.

Here, we report the development of several melting point ML models trained using a database of 19, 811 data points [21]. The models differ based on the ML technique (linear regression [22], deep learning, etc.), the use of 2D structural Padel features vs. 2D + 3D structural Padel features, and the degree of feature engineering used. Errors are quantified using 5-fold validation. Explainability is incorporated into the best-performing model via the SHAP algorithm [23], and a discussion of the accuracy of the best-performing melting point model on subsets of chemically relevant molecules (molecules containing only *C*, *H*, and *O*, for example) is given.

## Implementation

**Fig. 1:**
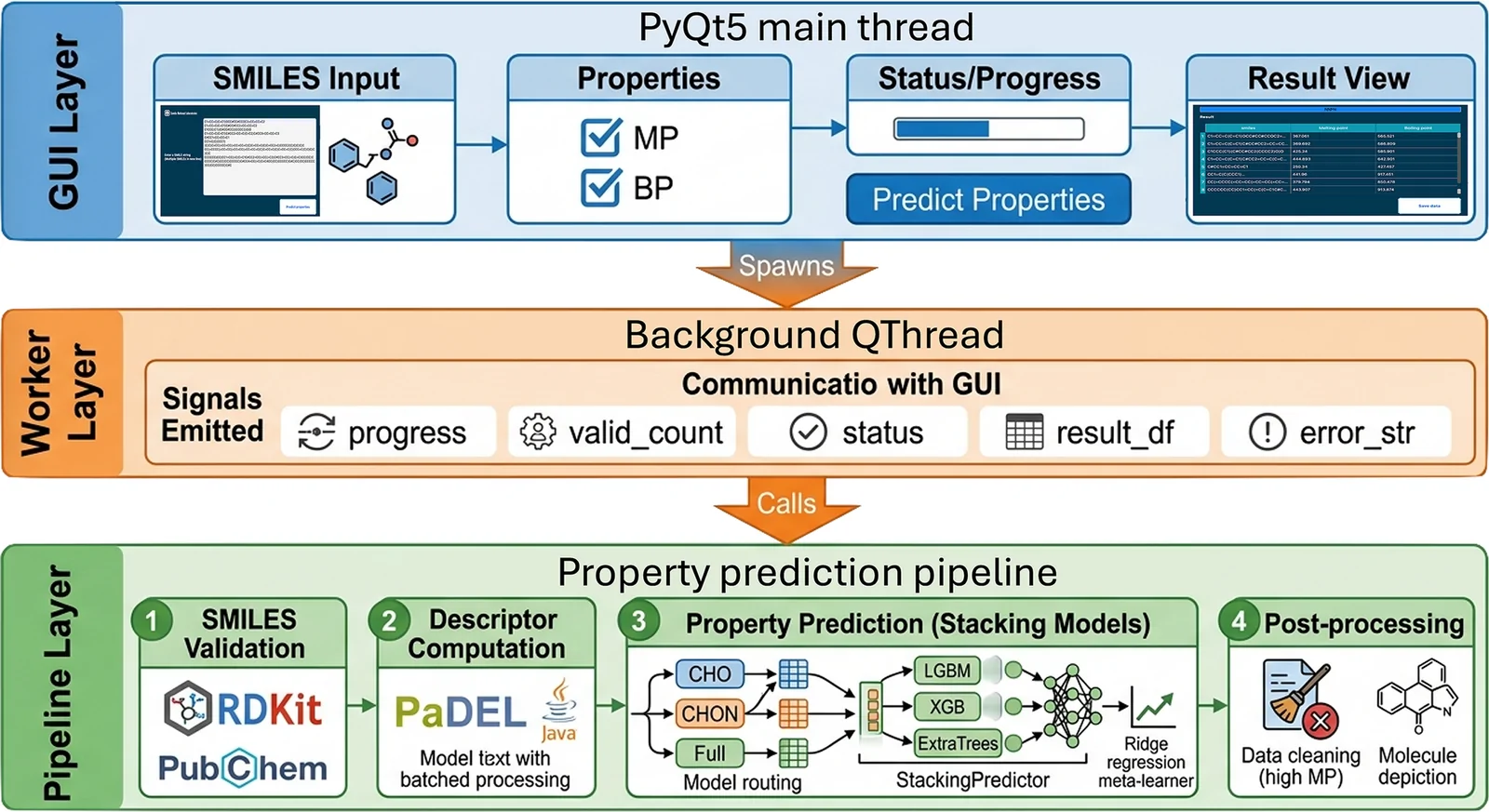
High-level system architecture of P2MAT. The application has a multi-threaded design. A PyQt5-based GUI manages user interactions. A dedicated background worker handles the four-step structural property prediction pipeline. This includes molecule validation, batch descriptor computation, and domain-specific stacking ensemble prediction

P2MAT is a desktop application that works on multiple platforms. It predicts the thermophysical properties of chemical compounds using SMILES input strings. This section explains the third-party packages that support the software, the technical setup that connects these components, the installation, the license, and the known limitations of the current version. Table 1 lists the main third-party libraries used in P2MAT. The software needs Python 3.12 and operates within a specific conda environment [24] (qsai), as defined in QSAI.yml. Figure 1 provides a schematic overview of how the different parts of P2MAT work together.

**Table 1:** Key third-party packages used in P2MAT

Package	Role	Version channel
PyQt5	Desktop GUI framework	pip
RDKit	SMILES validation and molecular manipulation	conda-forge
PaDEL-Descriptor (via padelpy)	Molecular descriptor computation	pip
scikit-learn	ML models and joblib serialization	conda-forge
XGBoost	Gradient-boosted tree regressor	conda-forge
LightGBM	Gradient-boosted tree regressor	conda-forge
PyTorch	Deep neural network (DNN) regressor	conda-forge
NumPy	Numerical array operations	conda-forge
Pandas	Tabular data management	conda-forge
Joblib	Model serialization and loading	conda-forge
Java (OpenJDK)	PaDEL-Descriptor runtime	system
PyInstaller	Frozen binary packaging	pip

***Graphical user interface***

The application window (GUI Layer), shown in Fig.1, is built using PyQt5 [25]. To keep the Qt event loop responsive during predictions, all blocking tasks are handled in a QThread subclass called PredictionWorker. This worker communicates with the main thread using four specific Qt signals: progress(int), result(object), invalid_smiles(list), and error(str). When the application starts, it loads the visible properties and the enabled state of each checkbox from a single config.json file. This setup allows users to change properties without needing to recompile or repackage the application.

***Molecular descriptor computation***

Molecular features are computed using PaDEL-Descriptor [26], a Java-based cheminformatics tool that generates both 2D and 3D descriptors from SMILES strings. The padelpy Python wrapper calls PaDEL through the system’s Java runtime, producing up to about 1,240 numerical descriptors per molecule. To process large query sets efficiently, SMILES strings are submitted to PaDEL in batches of 128 molecules by default. A divide-and-conquer fallback strategy ensures that if a batch call fails, the batch is split in half until individual molecules are processed. This way, one problematic structure does not stop the analysis of the remaining molecules. The temporary .smi input and .csv output files created during each PaDEL call are stored in the operating system’s temporary directory and are deleted right after they are read, whether the call succeeds or fails.

***SMILES validation***

 Input SMILES strings are checked for structure using RDKit [27] before calculating any descriptors. Specifically, Chem.MolFromSmiles processes each candidate string. A return value of None or any exception indicates an invalid structure. Invalid SMILES strings are sent back to the caller and shown to the user without interrupting predictions for the other molecules.

***Path resolution and packaging***

Resource paths for models, feature lists, CSS, and images are managed by the helper.resource_path utility. This tool checks during runtime whether the application is running as a standard Python script or as a PyInstaller-frozen binary (sys._MEIPASS). This method provides consistent and dependable access to assets in both deployment modes.

### Installation

P2MAT is open-source software available for download at https://doi.org/10.11578/ dc.20260318.3. Detailed instructions for setting up and using this software are available on the GitHub readme page, along with a visual representation that should help users get started directly.

P2MAT is distributed as a platform-specific installer that automates all prerequisite setup. Three platforms are currently supported: macOS (Apple Silicon), Windows (x64), and Linux (x86_64 / aarch64). Table 2 summarises the installer type, distribution format, install location, and approximate disk footprint for each platform.

**Table 2:** P2MAT installation summary across supported platforms

Platform	Installer	Install location	Disk
macOS (Apple Silicon)	install.command (bash)	$^{\sim }$/Applications/P2MAT/	∼3.5 GB
Windows (x64)	Install-P2MAT.ps1 (PowerShell)	%LOCALAPPDATA% \ P2MAT \	∼4 GB
Linux (x86_64 / aarch64)	install.sh (bash)	$^{\sim }$/.local/share/P2MAT/	∼4 GB

All three installers follow the same six-stage sequence: (1) verify the system and architecture, (2) install Java, (3) install Miniconda3, (4) create the qsai Conda environment from QSAI.yml, (5) copy application files, and (6) create a launcher or shortcut. It requires an active internet connection for the initial installation; all subsequent launches can be done offline. The macOS installer requires Homebrew as the package manager. The Linux installer automatically detects the distribution from /etc/os-release and selects the right package manager (apt, dnf, zypper, or pacman) to install OpenJDK,17. On Windows, Java can be obtained through winget or by direct MSI download if winget is not available. An optional Inno Setup wizard installer (P2MAT-v1.0.0-Windows-x64-Setup.exe) is provided for users who want a guided graphical installation experience. For advanced users who already have conda and Java installed, the application can be launched directly from the repository without running the specific installer.

conda env create -f QSAI.yml

conda activate qsai

cd P2MAT && python p2mat.py

### License

P2MAT is released under the BSD 3-Clause License.

Copyright © 2025, National Technology & Engineering Solutions of Sandia, LLC (NTESS). Under the terms of Contract DE-NA0003525 with NTESS, the U.S. Government retains certain rights in this software.

Redistribution and use in source and binary forms, with or without modification, are allowed if the following three conditions are met.

1. Redistributions of source code must keep the copyright notice, this list of conditions, and the disclaimer.
2. Redistributions in binary form must include the copyright notice, this list of conditions, and the disclaimer in the accompanying documentation.
3. Neither the name of NTESS nor the names of its contributors can be used to endorse or promote products derived from this software without explicit prior written permission.

The software is provided as is, without any express or implied warranty, including warranties of merchantability or fitness for a particular purpose. The BSD 3-Clause License is a permissive open-source license. It imposes minimal restrictions on reuse while protecting the name of the originating institution. A copy of the full license text is in the LICENSE file at the root of the P2MAT distribution.

### Limitations

The current release of P2MAT has the following known technical and usability limitations.

***Technical limitations***

- Platform support: The macOS distribution has been compiled and tested only on Apple Silicon (M-series) hardware. An Intel Mac build is unavailable because the melting-point DNN was trained with PyTorch’s Metal Performance Shaders (MPS) backend. Running the same artifact on an x86 CPU would need retraining on that hardware.
- Java dependency: PaDEL-Descriptor requires a Java runtime environment, specifically OpenJDK 11 or later. If Java is not installed or not in the system PATH, descriptor computation may fail due to a timeout or subprocess error. The installers take care of this requirement automatically. However, for manual installations, users must ensure that Java is properly installed and configured.
- PaDEL timeout and complex molecules: Each molecule has a 10-second timeout in PaDEL. Very large, highly branched, or structurally unusual molecules might exceed this limit, causing them to be skipped without notice after the divide-and-conquer method has been exhausted. Processing these molecules one at a time or increasing the timeout value may help with this problem.
- Applicability domain: All machine learning models for predicting melting and boiling points were trained on limited, curated datasets made mostly of organic molecules. As a result, predictions for structures outside the chemical space covered by the training data, such as inorganic salts, organometallic compounds, or molecules with very rare functional groups, might be unreliable. Since there is no automatic filter for applicability domains, users must carefully interpret results for unusual inputs.
- Internet requirement: An active internet connection is needed during the first installation to download Miniconda, the package dependencies, and OpenJDK. Offline installation is not supported.

***Usability limitations***

- Input format: P2MAT accepts only SMILES strings as input. Other common molecular representations, like InChI, MOL/SDF files, and CAS registry numbers, are not supported. They must be converted to SMILES externally before use.
- Batched descriptor computation: Molecular descriptors are created in batches of 128 SMILES strings using PaDEL-Descriptor, a Java-based application that runs as a subprocess. PaDEL starts once per batch instead of once for the entire dataset, causing the Java Virtual Machine (JVM) startup time to occur repeatedly, about $\lceil \frac{N}{128}\rceil$ times for N molecules. Since each JVM initialization usually takes several seconds, this cumulative time becomes significant for large datasets. This is the main reason processing hundreds of molecules can take several minutes.
- Single-session results: Prediction results are kept in memory only for the session. The system does not have history tracking or session persistence. Any results that are not exported to a CSV file before closing the window will be lost permanently.
- Minimum one property constraint: The graphical user interface requires users to select at least one property checkbox at all times. If all checkboxes are unchecked, the melting point option is automatically turned back on as a fallback. This design stops prediction runs from happening with no properties selected, even though it may be surprising for users who only want to check SMILES input without running a model.

## Method

**Fig. 2:**
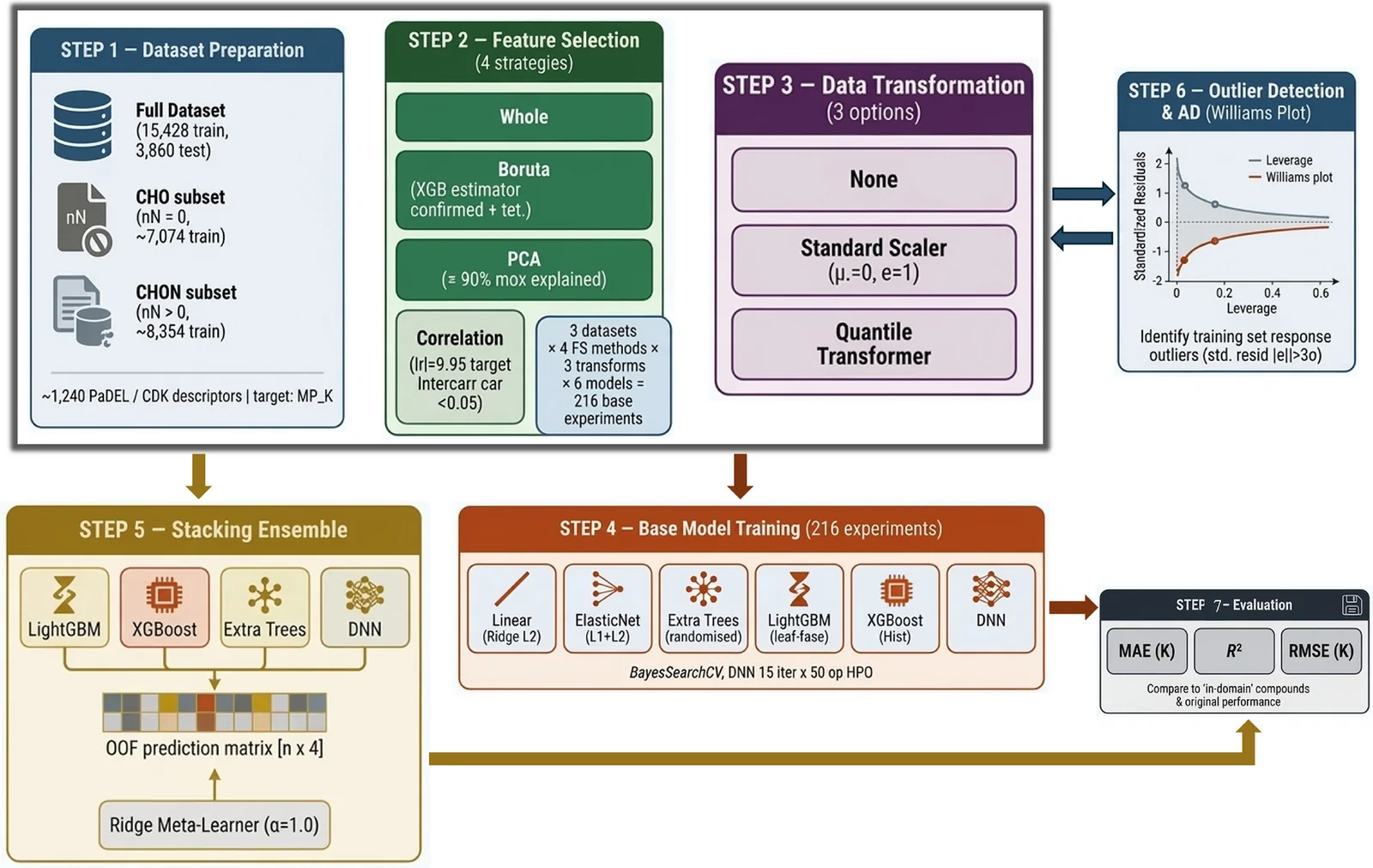
Schematic of the full modeling pipeline, from data preprocessing and feature selection to model training and evaluation

The modeling pipeline (Fig. 2) was created as a clear and fully reproducible framework. It included preprocessing methods, feature selection techniques, and machine learning algorithms across three chemically defined datasets: CHO, CHON, and FULL. Each dataset went through three input transformation methods: no transformation, StandardScaler, and Quantile Transformer. Four feature selection approaches were applied: Whole (no feature removal), Boruta [28], Pearson correlation [29] filtering, and principal component analysis (PCA) [30]. The resulting feature sets trained six regression models: Ridge [31], ElasticNet [32], Extra Trees [33], LightGBM [34], XGBoost [19], and a deep neural network (DNN) [35], resulting in a total of 216 distinct model configurations. For each configuration, we optimized hyperparameters independently using Bayesian search [36] to ensure fair comparison. The best-performing base learners were then combined into a two-level stacking ensemble [37]. Out-of-fold predictions served as meta-features for a Ridge regression meta-learner. This approach created a final model with lower generalization error than any individual model. We also conducted an applicability domain analysis using the Williams plot [38]. This analysis identified response outliers and high-leverage compounds, assessed the structural coverage of the training set, and evaluated the reliability of predictions on the test set. Finally, to understand the predictions of the top-performing tree-based models and identify the molecular descriptors that mainly drive changes in property prediction, we calculated Shapley Additive Explanations (SHAP) [23] using the TreeExplainer algorithm. This analysis provided precise feature attributions for each compound while meeting the key game-theoretic principles of efficiency, symmetry, and additivity.

### Data transformation

#### Standard Scaler Transformation

In this pipeline, StandardScaler was employed as one of three input transformation methods, together with no transformation and QuantileTransformer. To prevent data leakage, it was fitted exclusively to the training set. This approach centers each molecular descriptor to a mean of zero and scales it to unit variance. Consequently, models that are sensitive to feature gradients, such as Ridge, Elastic Net, and deep neural networks (DNNs), are less likely to be disproportionately affected by descriptors with large numerical ranges.

#### Quantile Transformer

The Quantile Transformer was adopted as an alternative to the Standard Scaler to better accommodate the skewed, heavy-tailed distributions frequently present in computed molecular descriptors, including topological indices and connectivity counts. Instead of scaling features to unit variance, this method transforms each feature’s marginal distribution into a uniform or approximately normal distribution. Consequently, it offers increased robustness to outliers, which might otherwise distort variance estimates and negatively influence the performance of distance-based or gradient-based machine learning algorithms.

### Feature selection

#### Boruta

Boruta feature selection approach [28] was designed to identify all features that provide meaningful information about the target variable, rather than stopping once a minimal sufficient subset had been found. In the current pipeline, XGBoost was used as the base estimator, configured with 100 estimators and tree_method=“hist” on the CPU. The algorithm was allowed to run for up to 100 iterations. In each iteration, shadow versions of all features were generated by permuting their values, and the model was trained on the combined set of original and shadow features. A feature was considered confirmed if its importance exceeded the maximum importance of the shadow features at the 100th percentile, which served as a stringent selection criterion. To reduce unnecessary computation, early stopping was applied after 20 consecutive rounds without change. Both confirmed and tentatively confirmed (weakly confirmed) features were retained according to the support criterion. This procedure yielded compact feature subsets ranging from 78 to 127 features, depending on the dataset (e.g., 101 for CHO and 127 for FULL). Overall, this represented approximately a 90% reduction from the original ∼1,100 to 1, 200 descriptors while preserving the most informative variables.

#### Correlation-coefficient

Feature selection was performed using a two-stage Pearson correlation [29] filter applied exclusively to the training set. In the first stage (termed relevance filtering), features (*X*) were retained only when the absolute Pearson correlation between each feature and the target variable (*Y*) exceeded 0.05. This step eliminated descriptors with little linear association with *Y*. In the second stage (referred to as redundancy reduction), the remaining features were ranked in descending order according to their correlation with the target and then pruned greedily. A feature was removed if its correlation with any previously accepted feature was greater than or equal to 0.85. This procedure reduced multicollinearity while preserving the most informative representative from each related feature cluster. The final subset was of moderate size and low redundancy, comprising approximately 306–389 features across the datasets.

#### PCA

Principal component analysis (PCA) [30] was used as a feature selection strategy to transform the scaled molecular descriptor space into an orthogonal, lower-dimensional representation. The method was configured to retain the smallest number of principal components (PCs) required to explain at least 90% of the total variance in the training set, while also enforcing a minimum of 10 components to prevent cases in which only a few PCs captured nearly all of the variance. As a result, between 84 and 113 PCs were retained, depending on the dataset, representing a substantial reduction from the original 1,100–1,200 descriptors. Although PCA reduces collinearity and noise by discarding low-variance directions, it also decreases interpretability because each component represents a combination of the original descriptors. More importantly, because PCA is an unsupervised method that maximizes variance rather than predictive relevance, it can weaken the relationship between features and target variables. This limitation was reflected in the observed model performance.

### Data split

**Fig. 3:**
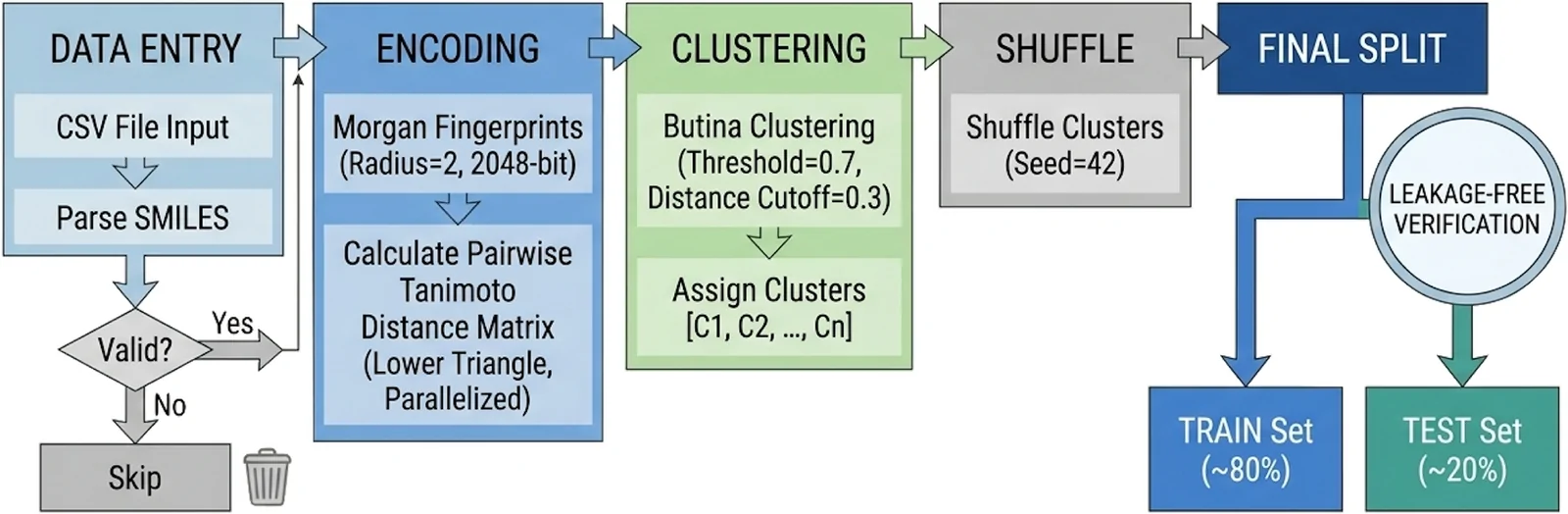
Butina clustering was used to split the data, as shown here. The full dataset was grouped based on Tanimoto similarity of molecular fingerprints. Clusters were then assigned to the training and test sets to minimize structural overlap. Table 3 presents the resulting distributions of pairwise similarities within and between the sets

To minimize structural overlap between training and test sets and reduce the risk of information leakage in model evaluation, a chemistry-aware cluster-based split was employed in place of a simple random partition. Morgan circular fingerprints [39, 40] (radius 2,2048 bits) were computed for all valid compounds using RDKit’s MorganGenerator. Pairwise Tanimoto distances [41, 42] were then computed in parallel across all compound pairs, and the Butina algorithm [43] was applied to cluster compounds at a similarity threshold of 0.8 (distance cutoff 0.2). The resulting clusters were randomly shuffled and greedily assigned to the test set until approximately 20% of the dataset was reached, ensuring that structurally similar compounds always reside in the same partition. The quality of the split was assessed by computing three Tanimoto similarity distributions: pairwise within-train, pairwise within-test, and the maximum cross-set similarity of each test compound to its nearest training neighbor. A low mean cross-set similarity and a small fraction of test compounds with nearest-neighbor similarity ≥0.8 confirm that the cluster-based partition effectively separates the chemical spaces of the training and test sets. Figure 3 illustrates the train-test splitting strategy used to minimize information leakage.

### Machine learning models

#### Ridge regression

Ridge regression [31] is a regularized extension of ordinary least squares (OLS). It is designed to reduce multicollinearity and limit overfitting by adding an $L_2$ penalty to the loss function. It aims to minimize the residual sum of squares while enforcing a constraint on the squared Euclidean norm of the coefficient vector:

$$\mathcal {L}(\textbf{w}) = |\textbf{y} - \textbf{X}\textbf{w}|_2^2 + \alpha |\textbf{w}|_2^2$$

where $\alpha \ge 0$ is the regularization hyperparameter. The closed-form solution, $\hat{\textbf{w}} = (\textbf{X}^\top \textbf{X} + \alpha \textbf{I})^{-1}\textbf{X}^\top \textbf{y}$, always exists. This holds true even when $\textbf{X}^\top \textbf{X}$ is singular, which improves numerical stability in high-dimensional descriptor spaces. As α increases, the coefficient magnitudes shrink toward zero. This reduces model variance, but it also increases bias. Unlike Lasso, Ridge keeps all features and applies the penalty evenly across correlated predictors instead of selecting one variable arbitrarily. In this study, Ridge Regression is the linear baseline for comparing the nonlinear models. We optimized the regularization strength, α, using Bayesian hyperparameter optimization over the range $[10^{-4},10^3]$ with a log-uniform prior. This approach reflects the wide dynamic range usually seen for this parameter in high-dimensional QSAR settings.

#### ElasticNet

 Elastic Net regression [32] combines the $L_1$ penalty from Lasso and the $L_2$ penalty from Ridge regression within a single optimization framework. This allows for variable selection and coefficient shrinkage to happen at the same time.

$$\mathcal {L}(\textbf{w}) = |\textbf{y} - \textbf{X}\textbf{w}|_2^2 + \alpha \left[ \rho |\textbf{w}|_1 + \frac{1-\rho }{2} |\textbf{w}|_2^2 \right]$$

where α controls the overall regularization strength and $\rho \in [0,1]$ (the l1_ratio) determines the balance between the two penalties. When ρ=1, Elastic Net becomes Lasso. When ρ=0, it becomes Ridge. The $L_1$ component encourages sparsity by shrinking irrelevant coefficients to exactly zero, which provides implicit feature selection. The $L_2$ component stabilizes the solution when there are groups of highly correlated descriptors. This is common in molecular fingerprint spaces, where similar fragments create correlated binary bits. Elastic Net was chosen over Support Vector Regression (SVR) [44] because it has a much lower computational cost on large datasets, training in seconds instead of minutes. It also fits well with high-dimensional, sparse descriptor matrices. Both α and ρ were optimized together using Bayesian search.

#### Extra trees regressor

 The Extra Trees (Extremely Randomized Trees) [33] algorithm is an ensemble learning method that builds a large number of unpruned decision trees and averages their predictions. It expands on the Random Forest framework [20] by adding another layer of randomness. In Random Forest, the optimal split threshold for each candidate feature is chosen. In contrast, Extra Trees randomly selects thresholds from the range of each feature. Specifically, at each node, *K* features are picked at random, and for each feature ($f_k$), a threshold ($t_k$) is sampled uniformly from ($[\min (f_k), \max (f_k)]$). The split ($(\hat{f}, \hat{t})$) that maximizes variance reduction is selected from this random set. This dual randomization of both features and thresholds further reduces the correlation among trees compared to Random Forest. It reduces ensemble variance while only slightly increasing bias. In practice, Extra Trees usually trains two to four times faster than Random Forest, as it avoids the computationally heavy threshold search during node splitting. In high-dimensional molecular descriptor datasets, this random splitting method acts as an effective form of implicit regularization. It helps prevent individual trees from overfitting to correlated fingerprint features. The main hyperparameters, such as the number of estimators, maximum tree depth, minimum samples needed for splitting and for leaf nodes, and the proportion of features considered at each split, were optimized using Bayesian search.

#### LightGBM

 LightGBM [34] is a gradient boosting framework created by Microsoft. It delivers top performance on tabular data through two main innovations compared to traditional gradient boosting machines (GBMs) [45]. The first innovation is Gradient-based One-Side Sampling (GOSS). It keeps all instances with large gradients or high errors, while randomly selecting instances with small gradients. This approach reduces data volume while maintaining the most important training signals. The second innovation is Exclusive Feature Bundling (EFB). This method finds sparse features that rarely take on nonzero values at the same time and merges them into a single bundle, which decreases the effective number of features.

Tree construction uses a histogram-based method. This bin’s continuous feature values into discrete buckets. As a result, the computational cost of finding splits drops from the expensive O(nlogn) needed by traditional GBMs to *O*(*B*), where *B* is the number of bins. Importantly, LightGBM uses a leaf-wise growth strategy. At each step, it splits the leaf that offers the greatest reduction in loss, no matter the tree’s depth. This approach usually leads to deeper, uneven trees that achieve lower training errors with fewer trees compared to the level-wise growth used by XGBoost and scikit-learn’s GBM. However, it needs careful regularization with parameters such as min_child_samples, num_leaves, and max_depth to avoid overfitting.

The model trains using the gradient and Hessian of the loss function, which is Mean Absolute Error in this study. It also supports parallel split finding across CPU cores through OpenMP.

#### XGBoost

 XGBoost (eXtreme Gradient Boosting) [19] is a scalable, regularized gradient-boosting implementation that builds on the classical GBM framework by adding $L_1$ and $L_2$ regularization terms directly into the tree-building goal. At iteration *t*, the regularized objective function is defined as:

$$\mathcal {L}^{(t)} = \sum _{i=1}^{n} l(y_i, \hat{y}_i^{(t)}) + \Omega (f_t), \quad \Omega (f) = \gamma T + \frac{1}{2}\lambda |\textbf{w}|^2 + \alpha |\textbf{w}|_1$$

where *T* denotes the number of leaves, $\textbf{w}$ represents the leaf weights, γ is the minimum loss-reduction threshold needed for splitting, and λ and α correspond to the $L_2$ and $L_1$ regularization penalties, respectively. XGBoost finds the optimal split by using a second-order Taylor expansion of the loss function, which includes both the gradient $g_i$ and the Hessian $h_i$. In this study, we use the histogram-based tree method. This method employs the same histogram binning strategy as LightGBM and improves the efficiency of finding splits in large datasets. Unlike LightGBM’s leaf-wise growth approach, XGBoost uses level-wise tree growth. It builds each tree to a fixed depth before going further, which usually leads to more balanced trees. This more cautious exploration of the hypothesis space may help with generalization on smaller datasets.

#### Deep neural network

**Fig. 4:**
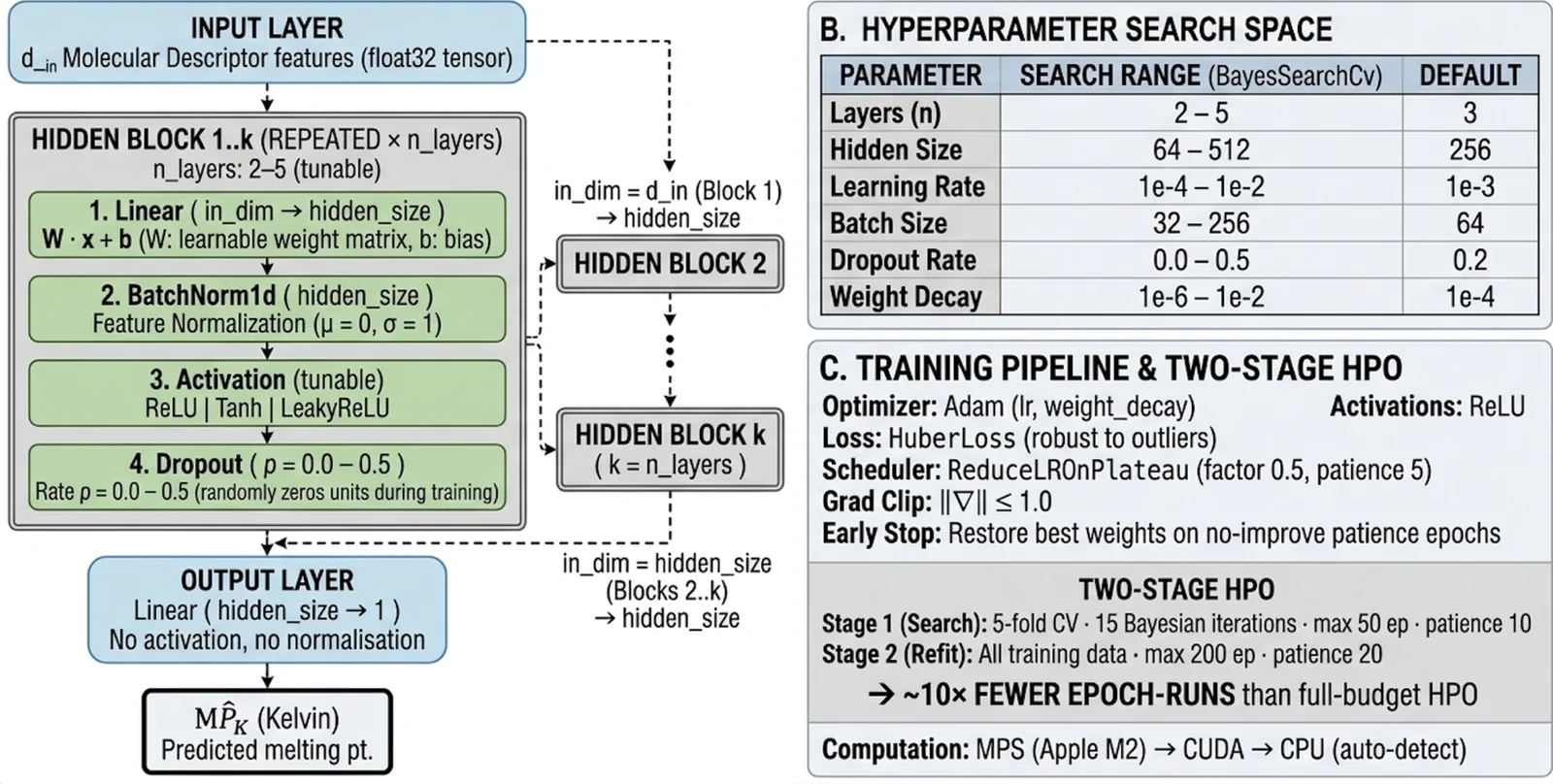
Deep Neural Network architecture diagram. The network consists of $n_{layers}$ fully-connected blocks, each containing a linear layer, Batch Normalization, a non-linear activation function, and dropout regularisation, followed by a single linear output neuron for regression

 The deep neural network (DNN) [35] used in this study is a fully connected feed-forward regression model. It is implemented in PyTorch and wrapped in a scikit-learn compatible interface to help integrate with the Bayesian hyperparameter optimization pipeline. The network architecture (shown in Fig. 4) includes $n_{layers}$ repeated blocks. Each block consists of a linear projection, batch normalization, a nonlinear activation function, and dropout regularization. This is followed by a single linear output neuron.

$$\textbf{h}^{(l)} = \text {Dropout}\left( \sigma \left( \text {BN}\left( \textbf{W}^{(l)} \textbf{h}^{(l-1)} + \textbf{b}^{(l)} \right) \right) \right) , \quad l = 1, \ldots , n_\text {layers}$$

Batch normalization is applied to each pre-activation in the mini-batch to reduce internal covariate shift and allow for higher learning rates. The activation function, (σ), is chosen through hyperparameter optimization from ($\text {ReLU}$), ($\text {Leaky ReLU}$), and (tanh). The network is trained using the Huber loss, which acts quadratically for small residuals and linearly for large residuals. This improves robustness to outliers caused by polymorphism or measurement inconsistencies in the dataset. Optimization is performed with Adam, using a learning rate sampled from $[10^{-4}, 10^{-2}]$ under a log-uniform prior. Learning rate annealing is carried out with ReduceLROnPlateau. Early stopping with a set patience helps prevent overfitting. To take advantage of Apple hardware, the model is assigned to the Metal Performance Shaders (MPS) backend when available. If MPS is not available, it falls back to CUDA and then to the CPU. To make Bayesian hyperparameter optimization more manageable, a two-stage training strategy is used. First, candidate configurations are evaluated with a lower budget of 50 epochs and a patience of 10 during the search phase. Then, the best-performing configuration is retrained from scratch using the full budget of 200 epochs and a patience of 20.

### Stacking ensemble

**Fig. 5:**
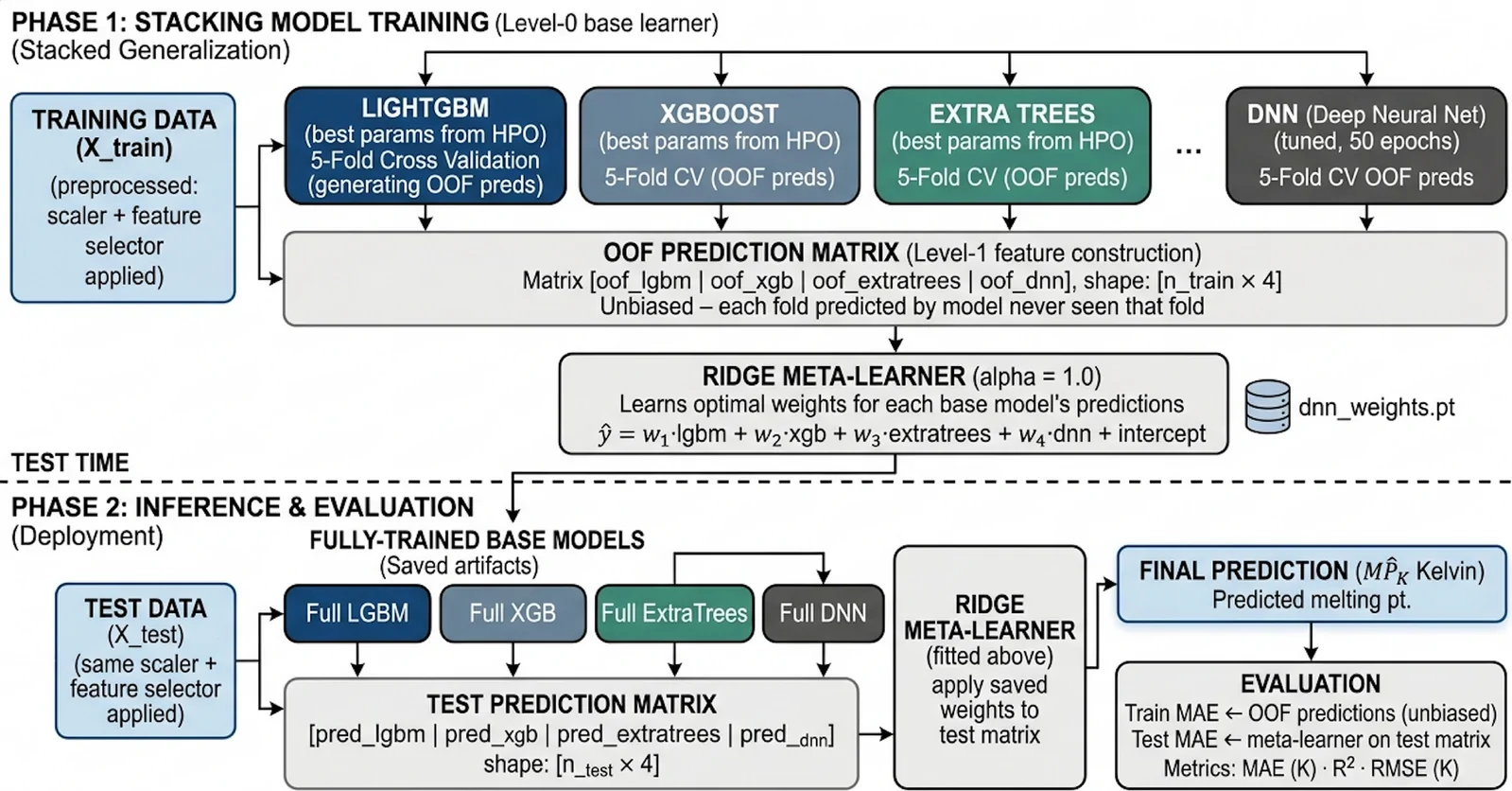
Stacking ensemble architecture diagram. The ensemble consists of four base learners (LightGBM, XGBoost, Extra Trees, DNN) that generate out-of-fold predictions on the training set to train a Ridge regression meta-learner. At inference time, the base learners produce predictions on the test set, which the meta-learner combines to produce the final output

The stacking ensemble (shown in Fig. 5) is a two-level meta-learning architecture designed to combine the strengths of LightGBM, XGBoost, Extra Trees, and a deep neural network (DNN) into a single model that has a lower generalization error than any individual component. In the first level, each base learner is trained separately using its own tuned hyperparameters, feature selection method, and data transformation strategy. This results in four unique predictors with different error profiles. To give the meta-learner an unbiased training signal and avoid data leakage, out-of-fold (OOF) predictions are created through five-fold cross-validation. Each base model is re-instantiated with its best saved hyperparameters and trained on four folds of the training data. It then generates predictions for the remaining held-out fold. This method results in a prediction matrix $\textbf{Z}\in \mathbb {R}^{n_\text {train} \times 4}$, where each column $\textbf{z}_k =[\hat{y}_1^{(k)},\ldots ,\hat{y}_n^{(k)}]^\top$ holds the OOF predictions from base model *k*. In the second level, a Ridge regression meta-learner is trained on $\textbf{Z}$.

$$\hat{y} = w_1 \hat{y}^{\text {LGBM}} + w_2 \hat{y}^{\text {XGB}} + w_3 \hat{y}^{\text {ET}} + w_4 \hat{y}^{\text {DNN}} + b$$

$L_2$ regularization in the ridge meta-learner prevents the model weights from leaning toward a single base learner. This helps use complementary information from all four contributors. During inference, the four fully trained base models make predictions for the test set. These predictions are then arranged into a matrix, $\textbf{Z}_{\textrm{test}} \in \mathbb {R}^{n_{\textrm{test}} \times 4}$, and passed through the fitted meta-learner to get the final prediction. To avoid unnecessary computation when adding new base models or retuning the meta-learner, the out-of-fold and test predictions for each base model are saved to disk as NumPy arrays and reloaded in future runs. Only predictions from new models are recalculated. The stacking framework does not depend on the feature spaces used by the individual base models. Each model applies its own fitted scaler and feature selector internally. This makes the approach strong when different feature-selection methods create descriptor subsets of varying sizes.

### Outlier detection

Outlier detection used the Williams plot method, a common applicability domain analysis technique recommended by the OECD [46] guidelines for QSAR model validation [38]. In this study, the Williams plot was created in the meta-feature space of the stacking ensemble. This four-dimensional space is defined by the out-of-fold predictions from the base models, which include LightGBM, XGBoost, Extra Trees, and DNN. It was not built in the raw descriptor space.

For each compound, two values were calculated: leverage (*h*) and standardized residuals. Leverage measures how much a compound impacts the centroid of the training set. It was determined using the formula $h_i = x_i^T(X^TX)^{-1}x_i$, where *X* is the OOF meta-feature matrix. The leverage threshold, $h^* =\frac{3(k+1)}{n}$, sets the boundary for the applicability domain, where *k* is the number of base models, and *n* is the training set size.

Standardized residuals were calculated by scaling the prediction errors of both training and test compounds with the standard deviation of the training residuals. This step ensures a uniform scale across data splits. Three types of outliers were identified: (i) response outliers, for which $|\text {standardized residual}|> 3$, indicating possible measurement errors or incorrect SMILES; (ii) leverage outliers, for which $h > h^*$, representing compounds outside the structural applicability domain; and (iii) bad leverage points, which meet both criteria, indicating the most questionable compounds.

### Model explainability

Model explainability was evaluated using SHAP (SHapley Additive exPlanations) [23], a framework for understanding machine learning model predictions. Based on cooperative game theory, SHAP values quantify how much each feature contributes to an individual prediction. In this study, we calculated SHAP values for the top-performing base learners, LightGBM and XGBoost, as well as for the Ridge meta-learner in the stacking ensemble. We used TreeExplainer for the tree-based models and LinearExplainer for the Ridge model. We determined global feature importance by averaging the absolute SHAP values across the test set. The SHAP beeswarm plot provided a comprehensive summary of the ensemble model’s behavior. In this visualization, each row represents a feature, ranked by mean absolute SHAP value from most to least important. Each dot corresponds to a single compound in the test set. The horizontal position of each dot shows the SHAP value: points to the right of zero indicate that the feature increased the predicted values, while points to the left indicate a decrease. Dot color indicates the raw feature value, with red for high values and blue for low values. This reveals whether a feature has a direct effect or a varying impact across compounds. This analysis identified the molecular descriptors that most significantly influenced prediction and showed how their effects differed among compounds.

### Uncertainty quantification

Two complementary measures of uncertainty were calculated for the stacking ensemble on each test set. First, we measured disagreement among the base models by computing the standard deviation of predictions from the four base learners (LightGBM, XGBoost, ExtraTrees, and DNN) for each test compound [47]. This metric acts as a per-sample indicator of uncertainty since compounds with greater disagreement between the base models are expected to have larger absolute prediction errors. We checked this relationship by calculating the Spearman rank correlation (ρ) [48] between the standard deviation of the base models and the absolute test error.

Second, we generated split-conformal prediction intervals using the out-of-fold (OOF) training residuals as the calibration set [49, 50]. The nonconformity score for each training compound was defined as $s_i = |y_i - \hat{y}i^{\textrm{OOF}}|$. We calculated the conformal quantile at a desired coverage level α as $q_\alpha = \textrm{quantile}(s, \lceil (n+1)\alpha \rceil / n)$, where *n* is the number of training compounds. We defined the prediction interval for a test compound as $[\hat{y} - q_\alpha , \hat{y} + q_\alpha ]$. This method offers a distribution-free coverage guarantee: if the calibration set is properly specified, the proportion of test compounds whose true melting points fall within the interval approaches α as *n* increases. We evaluated empirical coverage at nominal levels of $50\%, 60\%, 70\%, 80\%, 90\%, 95\%$, and 99% to check interval calibration across the entire confidence spectrum.

### Description of physics-based methods of boiling point

A variety of methods are available for estimating boiling points, each with its own set of pros and cons. Coefficient-based methods, such as DIPPR-based methods, rely on experimental data to regress and determine a set of coefficients that can then be used to calculate vapor pressure via a governing equation (“Design Institute for Physical Properties" 1996). Those coefficients can then be used to determine the temperature at which the vapor pressure equals 1 Atm, yielding the boiling point. When available, coefficient-based methods provide high-quality estimates of the boiling point. However, not all molecules have associated coefficients, and if experimental data is not available, those coefficients cannot be determined. Group contribution methods offer an alternative to coefficient-based methods. Group contribution methods assign a value to each chemical group. These values are obtained by regression from a wide range of boiling-point data. By applying these values to a working equation, boiling-point estimates within a few degrees of experimental measurements are often possible. The Joback method is a popular method for estimating boiling points. Based on a set of 438 molecules, the Joback method has an average absolute error of 12.9 °C [51]. Brown and Stein improve on the Joback method by adding a correction term [52]. Based on a dataset of 6, 584 boiling points, the Brown-Stein method has an absolute average error of 20.4 °C. Group-contribution methods are easy to implement and can provide reasonable boiling-point estimates. They work best for molecules similar to those found in the database used in regression. Group contribution methods do not have values for every possible group and cannot be applied to molecules that lack a represented chemical group. If the desired group is unavailable, other methods must be used. More rigorous, physics-based methods, such as the Equation of State (EoS), have been proven to provide very accurate vapor pressures while often running on just a laptop’s resources. Examples here include the Saft-gamme Mie, SAFT-VR, Peng Robinson, and Cubic Plus Association (CPA) [53]. Like coefficient-based methods, EoS methods require experimental data to estimate parameters. However, this is not an impediment, as experimental data using a variety of different physical properties are usually available.

## Results and discussion

This section provides a detailed overview of the QSAR melting-point prediction pipeline (considering 2D features) across three chemical-space datasets: CHO, CHON, and Full. Similar analyses of the melting point (considering 3D features) and the boiling point are presented in the supplementary document.

### Dataset

Three separate datasets were created from the curated experimental melting-point database to evaluate model performance across chemical spaces with increasing diversity. The CHO subset includes compounds made only of carbon, hydrogen, and oxygen. It represents aliphatic and aromatic oxygenates, such as alcohols, ethers, aldehydes, ketones, and carboxylic acids. The CHON subset builds on the CHO set by adding nitrogen-containing compounds, which include amines, amides, imines, nitro groups, and heterocyclic structures. The Full dataset includes all compounds, regardless of their elemental makeup. This dataset contains halogenated, sulfur, phosphorus, and metal-containing molecules, along with CHO and CHON compounds.

#### Data partitioning

The complete dataset was divided into 15, 428 training compounds and 3, 860 test compounds using Butina clustering with a Tanimoto similarity threshold of 0.7. In the training set, the mean pairwise Tanimoto similarity was 0.142, and the median was 0.107. In the test set, the corresponding values were 0.139 and 0.105. Across both sets, the mean maximum similarity between each test compound and its nearest training neighbor was 0.121, with a median of 0.094 and a maximum of 0.698. No test compound showed a nearest-neighbor similarity that met or exceeded the 0.7 threshold. These results (shown in Table 3) indicate that the cluster-based split effectively reduced structural overlap between the training and test sets, which helps prevent information leakage during model evaluation.

**Table 3:** Tanimoto similarity statistics are presented for the training and test sets, together with the maximum similarity of each test compound to its nearest neighbor in the training set

Metric	Train	Test	Cross
Compounds	15.428	3,860	–
Mean similarity	0.142	0.139	0.121
Median similarity	0.107	0.105	0.094
Max similarity	1.000	1.000	0.698
% pairs ≥ threshold (0.7)	3.1	2.9	0.0

The low mean and median similarities observed within and between the sets, along with the absence of test compounds with similarity values above the 0.7 threshold, confirm that the cluster-based split effectively minimizes structural overlap

Table 4 summarizes the resulting sample sizes after partitioning the data. The full dataset is about 2.4 times larger than the CHO and CHON datasets. This highlights the wider range of the complete experimental collection. Since the CHO and CHON subsets have overlapping compounds, the differences in model performance between these two sets mainly show the effect of including nitrogen-containing chemistry. They do not reflect differences attributable solely to dataset size.

**Table 4:** Data demography for the three datasets used in melting-point prediction

Dataset	$n_\text {Total}$	$n_\text {Train}$ (80%)	$n_\text {Test}$ (20%)
CHO	6,957	5,583	1,374
CHON	8,078	6,431	1,647
Full	19,288	15,428	3,860

All counts refer to unique compounds after curation and duplicate removal

### Model performance

#### Individual model performance

Before stacking, six base learners were trained independently using every combination of the three datasets and four feature-selection methods: the full feature set, Boruta, correlation-based selection, and principal component analysis (PCA). This process led to 72 individual training experiments. Table 6 summarizes the mean absolute error (MAE, in K) for each combination.

Across all three datasets, several consistent patterns appear. Gradient-boosted tree ensembles, specifically LightGBM (LGBM) and XGBoost, have the lowest mean absolute errors (MAEs) among the individual models in every instance. For the Full dataset, LGBM with correlation-based feature selection produced a test MAE of 28.29 K. For the CHO dataset, LGBM using the complete feature set achieved 26.46 K. For the CHON dataset, XGBoost with the complete feature set reached 29.45 K. In contrast, the linear models, Ridge Regression and ElasticNet, perform about 3 to 5 K worse under every feature-selection strategy. This reinforces the idea that melting-point variation across chemical space is inherently nonlinear.

Although ExtraTrees does not match the performance of LGBM or XGBoost, it consistently outperforms all linear regressors. The deep neural network (DNN) shows the most variability across feature-selection strategies. It performs poorly when trained on the full raw feature set, with MAE values as high as 43.57 K on the Full dataset. However, it becomes competitive with ExtraTrees when using Boruta-selected features, achieving 29.41 K on Full and 30.70 K on CHO. This pattern likely reflects the DNN’s need for a more compact and less collinear input space. Lastly, PCA-based feature selection consistently yields the highest MAE across all models. This indicates that the principal components explaining the greatest variance in the descriptor matrix are not the most informative for predicting the target property.

#### Stacking ensemble performance

A stacking ensemble was created by training a Ridge meta-learner on the out-of-fold predictions from the four best-performing base models: LightGBM, XGBoost, ExtraTrees, and a deep neural network (DNN). Table 5 shows the training and test performance of the stacking ensemble for each dataset, using the full feature set without removing outliers. The results show that the stacking ensemble achieved better generalization, as evidenced by reduced overfitting on the training data. Table 6 further expands this comparison by including all feature-selection strategies.

**Table 5:** The stacking ensemble’s performance was assessed using three datasets with the full feature set and no outlier removal

Dataset	Training performance			Testing performance		
	MAE(K)	$R^2$	RMSE(K)	MAE(K)	$R^2$	RMSE(K)
CHO	24.12	0.8456	33.77	25.29	0.83	36.28
CHON	27.89	0.7163	37.81	28.73	0.70	38.62
Full	25.86	0.8377	36.06	27.32	0.82	38.21

Training metrics came from in-sample out-of-fold (OOF) predictions. Test metrics were taken from the held-out 20% test set

**Table 6:** Test MAE (K) of all models, including the stacking ensemble, across datasets and feature-selection strategies

Dataset	FS	LR	E.Net	E.Trees	LGBM	XGB	DNN	Stack
Full	Whole	32.53	32.18	29.68	28.49	28.50	43.57	**27**.**32**
	Boruta	36.36	36.36	29.85	28.61	28.69	29.41	**27**.**33**
	Correlation	33.43	33.40	29.69	28.29	28.57	32.91	**27**.**07**
	PCA	48.88	48.88	41.47	39.37	39.21	43.78	**29**.**08**
CHO	Whole	30.33	30.23	27.87	26.46	26.90	35.95	***25.29***
	Boruta	34.35	34.36	28.17	26.98	27.44	30.70	**26**.**10**
	Correlation	31.71	31.63	28.45	26.84	27.16	30.17	**25**.**52**
	PCA	47.16	47.15	41.78	39.61	39.47	40.14	**27**.**83**
CHON	Whole	32.79	32.84	30.75	29.53	29.45	39.74	**28**.**73**
	Boruta	34.93	34.92	31.27	30.69	30.25	31.63	**29**.**39**
	Correlation	33.82	33.72	30.72	29.61	29.76	33.20	**28**.**81**
	PCA	42.78	42.78	41.40	39.62	39.77	40.58	**30**.**94**

*FS* feature selection, *LR* Linear regression, *E.Net* Elastic Net regression, *E.Trees* Extremely Randomized Trees Regressor, *LGBM* Light Gradient Boosting Machines, *XGB* Extreme Gradient Boosting, *DNN* Deep Neural Network

Bold entries mark the lowest MAE for each row (best model per dataset–feature-selection combination)

Across all datasets, the stacking ensemble reduced test MAE compared to the best individual model. For the CHO dataset, test MAE decreased from 26.46 to 25.29 K, a 4.4% reduction. For CHON, the MAE declined from 29.45 to 28.73 K, a 2.4% improvement. Similarly, for the Full dataset, the MAE dropped from 28.29 to 27.07 K, showing a 4.3% reduction (see Table 6 for the best stacking setup for each dataset). Although these improvements are modest, they are consistent. This suggests that the four base learners capture different sources of variance, and the meta-learner adjusts each model’s contribution based on the chemical subspace where it performs best. Among the datasets, CHO had the highest test-set $R^2$ of 0.829, likely due to its narrower, more uniform chemical space. In contrast, CHON had a lower $R^2$ of 0.701, likely due to the greater structural diversity associated with nitrogen-containing functional groups and more complex interactions, such as intramolecular hydrogen bonding and amide resonance stabilization. These interactions are more difficult to characterize with PaDEL descriptors alone. The Full dataset showed an intermediate $R^2$ of 0.818, benefiting from a larger training set and greater chemical diversity. Finally, the small difference between training and test performance–for instance, 24.12 K versus 25.29 K for CHO–suggests that the stacking ensemble generalizes well without significant overfitting.

The stacking ensemble shows its biggest improvement over the individual models with PCA-based feature selection. For instance, XGBoost performs 29.08 K compared to 39.21 K with the full feature set. This indicates that the meta-learner is particularly good at finding predictive signals when the base models work in a compressed input space that loses some information. Throughout all 12 datasets and feature selection combinations, the stacking ensemble consistently ranks first, highlighting the strength of the meta-learning approach.

**Fig. 6:**
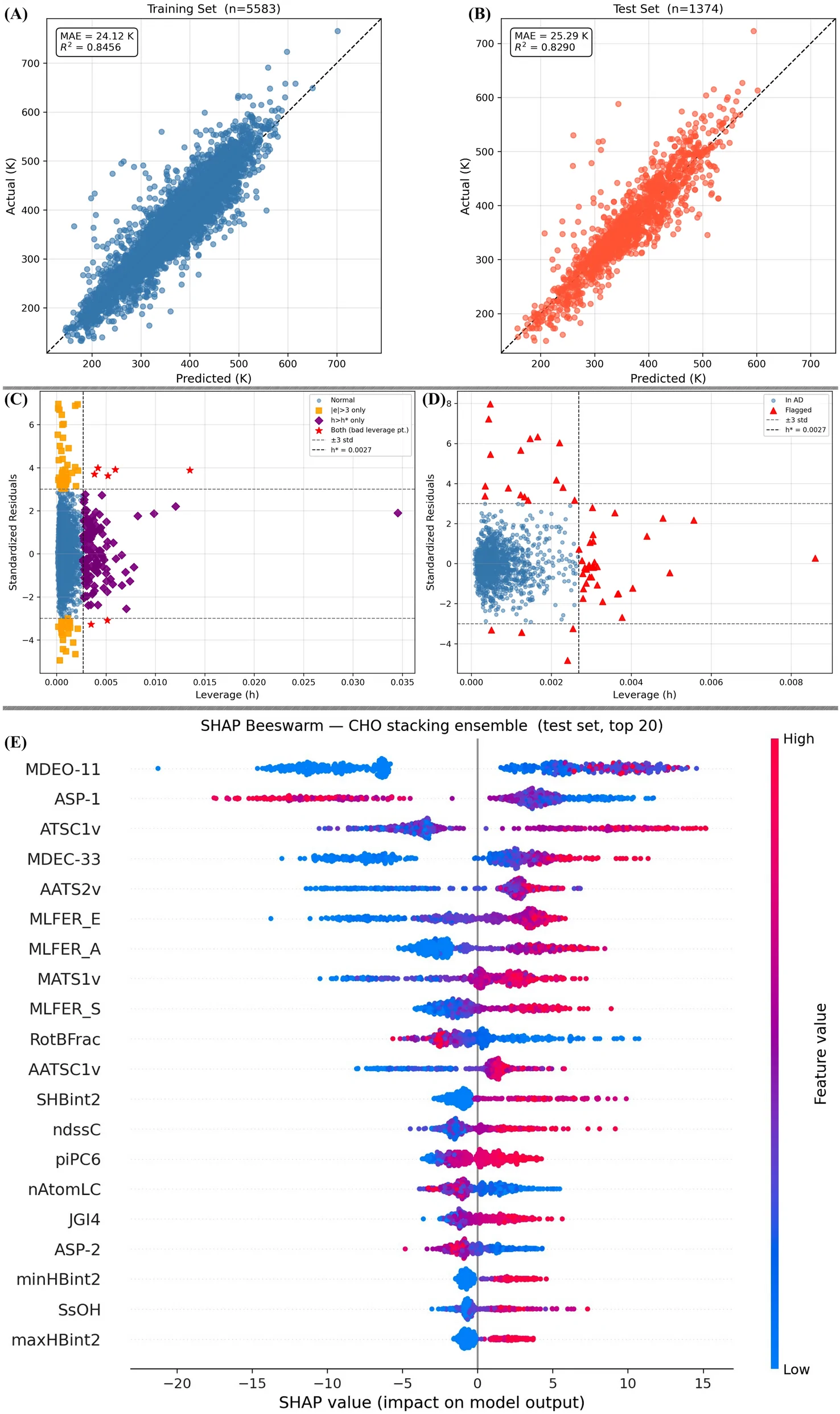
Panel **A**, **B** Stacking ensemble predicted vs. experimental melting points for the **CHO** dataset (whole features, Standard Scaler). The dashed diagonal line represents perfect prediction. Color density reflects point overlap. Panel **C**, **D** Williams plot for outlier detection. Each point is a training compound plotted at its leverage $h_i$ (x-axis) against its standardised residual $\varepsilon _i$ (y-axis). Vertical dashed line: leverage warning threshold $h^* = 0.00269$. Horizontal dashed lines: |ε|=3. Compounds in the upper/lower region (|ε|>3) are response outliers; those to the right of $h^*$ are leverage outliers; those satisfying both criteria are bad leverage points. Panel **E** SHAP beeswarm plot for model explainability (500 test compounds, whole features, Standard Scaler). Features ranked by mean $|\text {SHAP}|$ (K). The top-ranked feature MDEO-11 (modified detour matrix eigenvalue, $\overline{|\text {SHAP}|}=8.4$ K) exhibits a wide spread reflecting structural diversity in CHO space; ASP-1 (asphericity, rank 2) shows a clean negative monotone relationship (red left, blue right)

Figure 6A, B shows scatter plots comparing stacking-ensemble predictions with experimental melting points for the training and test sets of each dataset. The close grouping of the points along the diagonal indicates a strong agreement between predicted and observed values. As expected, the training scatter (panel A) is more tightly clustered than the test scatter (panel B), showing a slight advantage in performance for the training set. Noticeable deviations from the diagonal occur at the extremes of the melting-point range. Compounds with experimental melting points below approximately 200 K tend to be overpredicted, while those above 550 K tend to be underpredicted. This compression toward the mean is a known issue in QSAR models trained on right-skewed target distributions and likely reflects the limited number of compounds with extreme melting points in the training data.

#### Outlier detection using stacking ensemble model

Figure 6C, D shows the Williams plots for train and test data. In each plot, the horizontal axis represents the leverage, $h_i$, of a training compound. This reflects its structural distance from the training centroid in descriptor space. The vertical axis displays the standardized residual, $\varepsilon _i = (y_i - \hat{y}_i)/s$, where *s* is the standard deviation of the training residuals. The dashed vertical line at $h = h^*$ marks the boundary of the applicability domain. The horizontal dashed lines at $\varepsilon = \pm 3$ indicate the threshold for response outliers. Compounds in the upper-left quadrant (|ε|>3, $h \le h^*$) are response outliers without structural issues and likely show incorrect experimental values. Those in the lower-right quadrant ($h > h^*$, $|\varepsilon | \le 3$) are leverage points that the model predicts well. Compounds in the upper-right quadrant are bad leverage points that raise both types of concerns. To assess whether removing response outliers from the training set improves model generalization, the entire stacking pipeline was retrained from scratch after excluding the identified outliers (|ε|>3 and bad leverage points). Pure leverage outliers (h>h∗ only) were kept because, while they are structurally unusual, not having a large prediction error does not suggest measurement error.

**Table 7:** Williams plot applicability-domain analysis and effect of outlier removal from the stacking ensemble test performance

Dataset	$n_\text {train}$	$n_\text {train}^\text {clean}$	$n_\text {test}$	$h^{*}$	Training outliers			MAE (K)		$R^{2}$	
					|ε|>3	$h>h^{*}$	Both	All	Clean	All	Clean
CHO	5,583	5,509	1,374	0.003	74	127	7	25.29	25.93	0.83	0.82
CHON	6,431	6,363	1,647	0.002	68	93	4	28.73	29.01	0.70	0.70
Full	15,428	15,224	3,860	0.001	204	318	14	27.32	27.23	0.81	0.82

MAE$_\text {all}$ and $R^2_\text {all}$: metrics on the complete test set; MAE$_\text {clean}$ and $R^2_\text {clean}$: metrics on the complete test set when the model is trained on clean training data (after removing outliers)

$n_\text {train}^\text {clean}$: Number of train compounds after removing outliers. $h^{*} = 3(p+1)/n$, where p=4 base models and $n=n_\text {train}$, rounded to 3 decimal places. |ε|>3: response outliers (standardised residual >3σ); removed in the cleaned retrain. $h>h^{*}$: pure leverage outliers; retained (structurally unusual but not poorly predicted). Both: bad leverage points satisfying both criteria; removed in the cleaned retrain

Table 7 summarises the AD statistics for all three datasets. Data cleaning after removing outliers from the training data resulted in noticeably tighter fit sets across all three datasets. Training MAE improved (not shown in the table for space limitation) from 24.12 to 22.28 K for CHO, from 27.89 to 26.50 K for CHON, and from 25.86 to 24.03 K for the Full dataset. This confirms that the removed compounds were true high-residual observations that distorted the meta-learner’s fit. However, the effect on test-set generalization was inconsistent. For the Full dataset, removing 204 compounds (1.3% of the training data) led to a slight improvement of 0.09 K in test MAE (27.32–27.23 K; +0.3%). In contrast, the cleaned models performed slightly worse for CHO and CHON, with test MAE increased by 0.64 K (+2.5%) for CHO and by 0.28 K (+0.97%) for CHON. This decline suggests that the flagged compounds in the smaller datasets were not erroneous measurements but valid compounds located at the structural extremes. Removing these compounds reduced the model’s coverage of the melting-point distribution. Therefore, the original, uncleaned models were retained as the primary models across all three datasets.

#### Explainability of stacking ensemble model

Figure 6E shows the importance of each feature and how they influence the predicted melting point based on SHAP analysis. The results reveal patterns that make sense both chemically and physically. In each plot, features are listed from top to bottom according to their mean absolute SHAP values, and each dot represents an individual compound. The horizontal position of a dot reflects its SHAP contribution to the predicted melting point. Positive values increase the prediction, while negative values decrease it. The color of the dot indicates the raw feature value, with red signifying high values and blue indicating low values.

In the CHO dataset, the top features, especially the molecular distance edge descriptor MDEO-11 [54] and the average simple path index ASP-1 [55], shows the key role of global molecular structure and specific atomic connections in determining activity. Next in importance, van der Waals volume-weighted spatial autocorrelation descriptors like ATSC1v and AATS2v suggest that the arrangement of steric bulk significantly affects target interactions [55]. The model’s predictive ability is also strongly influenced by thermodynamic solvation parameters from Abraham’s Molecular Linear Free Energy Relationship descriptors, including MLFER_E, MLFER_A, and MLFER_S, which capture polarizability and hydrogen-bond acidity [56]. Additionally, local electronic environments, as indicated by electrotopological state indices such as SHBint2 and SsOH  [57], along with molecular flexibility, as characterized by the fraction of rotatable bonds (RotBFrac) [55], provide a clearer picture of ligand-binding potential. Overall, the SHAP profile for the CHO dataset shows that model predictions mainly rely on large-scale structural connectivity, then steric distribution and solvation thermodynamics.

In the CHON dataset (Fig. S1 (E) in the supplementary document), which includes nitrogen in the chemical space, the feature ranking shifts significantly toward descriptors associated with hydrogen bonding and electronic effects. MLFER_E moves to the top position, while maxHBint2, defined as the highest intramolecular hydrogen bond at a two-bond distance (r = +0.92), and SHBd, the total of hydrogen-bond donor E-state values [57], ranked second and third, respectively. This trend highlights the strong role of nitrogen-mediated donor-acceptor interactions in crystal cohesion. In the Full dataset (Fig. S2 (E) in the supplementary document), MLFER_S, which reflects Abraham’s dipolarity/polarizability, outshines all other features, with an average SHAP value of 11.4 K and a range from −42 to +22 K. This prominence mostly comes from the large polarity differences between ionic and zwitterionic compounds, like sulfonates and phosphates, compared to nonpolar hydrocarbons that define this broader chemical space.

Across all three datasets, MLFER_E, MLFER_S, and MLFER_A consistently rank among the most important predictors, reinforcing the theoretical importance of linear free-energy relationships in melting-point prediction [56]. In contrast, the fraction of rotatable bonds (RotBFrac [55]) is the only descriptor that consistently shows a negative SHAP direction, ranking 10th, 5th, and 6th in the CHO, CHON, and Full datasets, respectively. Increased flexibility affects the entropic penalty during crystallization, lowering the melting temperature regardless of elemental makeup. Overall, this SHAP-based profile supports the models’ physical interpretability, showing a shift from macro-structural connectivity to steric distribution and solvation thermodynamics, and finally to local electronic environments. It also offers molecular-level insight for designing compounds with specific melting points. Section 1.3 of the supplementary document describes each feature presented in the three datasets using our SHAP analysis.

#### Uncertainty quantification of stacking ensemble predictions

**Fig. 7:**
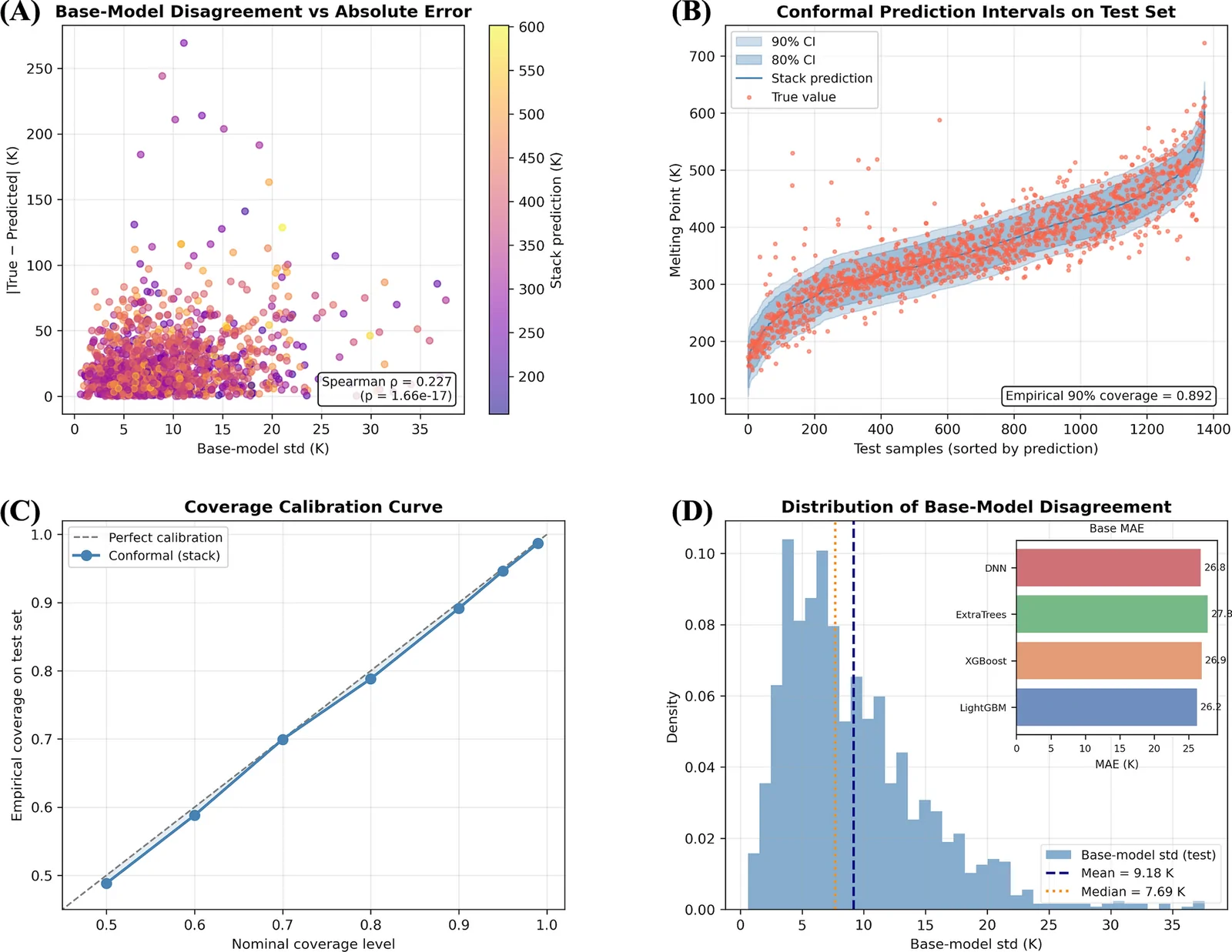
A four-panel uncertainty analysis was carried out for the **CHO** dataset stacking ensemble using the complete feature set with StandardScaler ($n_\text {test}=1{,}374$). Panel **A** illustrates the relationship between base-model disagreement and absolute error. It features a scatter plot showing the standard deviation of predictions from the four base models (LightGBM, XGBoost, ExtraTrees, and DNN) against the absolute prediction error $|y - \hat{y}|$ for each test compound. Points are colored based on the stack prediction value (plasma scale). The Spearman rank correlation (ρ=0.227, p<0.0001) suggests that greater disagreement among models is a statistically significant, though modest, sign of increased prediction error. Panel **B** shows conformal prediction intervals for the test set, with compounds arranged by increasing stack prediction value along the x-axis. Blue shaded bands indicate the symmetric 80% and 90% split-conformal prediction intervals. The solid line represents the stack prediction, while red dots show the true experimental melting points. The empirical 90% coverage (89.2%) is close to the nominal 90% target (±1.2 percentage points), which supports calibration. The conformal quantile is $q_{90} = \pm 53.3$ K. Panel **c** presents the coverage calibration curve, plotting nominal coverage against empirical coverage across seven levels (50%–99%). The dashed gray diagonal indicates perfect calibration. The conformal curve closely follows it across all levels, indicating no systematic over- or under-coverage. Panel **d** displays the distribution of base-model disagreement as a density histogram of the base-model standard deviation across all test compounds (mean =9.18 K, with the median marked by dashed lines). The inset horizontal bar chart shows the individual test MAE of each base model (LightGBM, XGBoost, ExtraTrees, and DNN), providing context for interpreting the level of disagreement

Prediction uncertainty was characterized through two complementary approaches: base-model disagreement and split-conformal prediction intervals. Figure 7 presents the four-panel uncertainty diagnostic for each dataset.

***Base-model disagreement***: For each test compound, the standard deviation of the four base-learners predictions (LightGBM, XGBoost, ExtraTrees, DNN) were computed as a per-sample epistemic uncertainty proxy. The Spearman rank correlation between this standard deviation and the absolute prediction error was ρ=0.227 (p<0.0001) for CHO, ρ=0.175 (p<0.0001) for CHON, and ρ=0.201 (p<0.0001) for Full, confirming that higher inter-model disagreement is a statistically significant, though modest, signal of elevated prediction error. The mean base-model standard deviation across test compounds was 9.18 K (CHO), 9.95 K (CHON), and 10.08 K (Full), indicating a broadly similar level of base-learner diversity across all three chemical spaces.

***Conformal prediction intervals***: Split-conformal intervals were constructed using the out-of-fold (OOF) training residuals as a calibration set. The non-conformity score for each training compound was defined as $s_i = |y_i - \hat{y}_i^\text {OOF}|$, and the conformal quantile at coverage level α was computed as $q_\alpha = \text {quantile}(s,\, \lceil (n+1)\alpha \rceil /n)$. The resulting symmetric prediction interval for each test compound is $[\hat{y} - q_\alpha ,\; \hat{y} + q_\alpha ]$. At a nominal 90% coverage level, the conformal quantiles were ±53.3 K (CHO), ±59.6 K (CHON), and ±56.5 K (Full), with empirical test-set coverages of 89.2%, 89.2%, and 88.8%, respectively — all within 1.2 percentage points of the nominal target. The close agreement between nominal and empirical coverage across all seven evaluated levels (50%–99%) confirms that the conformal intervals are well-calibrated and reliably bound the true melting point without systematic over- or under-coverage. The wide 90% interval widths reflect the intrinsic scatter of the melting-point distribution rather than a deficiency of the model: the distribution-free guarantee holds regardless of the underlying data distribution, and the intervals correctly widen for compounds in sparsely populated regions of chemical space where individual base-model errors are larger.

#### Benchmark analysis

To put P2MAT’s predictive performance into context, we compared our top stacking model (MAE = 25.29 K, RMSE = 36.28 K, and $R^2$ = 0.83 on the internal test set) with seven melting-point prediction models reported over the last two decades, as shown in Table 8. For each benchmark study, we applied P2MAT directly to the authors’ original compound set and provided MAE, RMSE, and $R^2$ alongside the published values. Lower error values and higher $R^2$ indicate better performance.

**Table 8:** Comparison of published melting-point prediction models with P2MAT

Ref.	$n_\text {comp}$	Method	Published performance			P2MAT performance			MMTS	LF(%)
			MAE	RMSE	$R^2$	MAE	RMSE	$R^2$		
A. Jain [14]	1, 215	MLR	33.20			25.51	37.17	0.86	0.623	34.05
Karthikeyan [58]	4, 173	ANN	32.60	49.30	0.66	22.20	35.65	0.68	0.685	45.13
Modarresi [59]	323	MLR		42.00	0.66	20.88	32.08	0.80	0.574	34.94
Tetko [60]	21, 840	ASNN		38.00		21.28	35.10	0.83	0.842	66.01
Sivaraman [16]	21, 840	GPR	30.03		0.77	21.28	35.10	0.83	0.842	66.01
McDonagh [61]	1, 100	RF		41.30	0.75	18.01	27.43	0.90	0.665	42.21
Bergstr$\ddot{\text {o}}$m [62]	277	PLS		49.80	0.57	40.91	48.86	0.03	0.415	10.26

Published performance metrics are taken directly from each reference; P2MAT performance (right-most three columns) is computed by applying our best stacking model to each author’s own compound set

$n_{comp}$ Number of chemical compounds, *MLR* Multiple linear regression, *ANN* Artificial neural network, *ASNN* Associative neural network, *GPR* Gaussian process regression, *RF* Random forest, *PLS* Partial least squares, *MMTS* Mean Maximum Tanimoto Similarity, *LF* Leakage fraction in %

To better understand the performance differences, given the overlap in training data, we measured structural similarity between our training set and each external dataset using two metrics derived from Morgan circular fingerprints (ECFP4; radius = 2, 2048 bits) implemented in RDKit [27]. For each compound in one dataset, we identified the highest Tanimoto coefficient with any compound in the other dataset using bulk similarity search. We defined the mean maximum Tanimoto similarity (MMTS) as the average of these maximum values per compound. This serves as a continuous measure of chemical space overlap that does not depend on a threshold. We defined the leakage fraction (LF) as the percentage of compounds whose nearest-neighbor Tanimoto similarity was at least 0.8, which is the near-duplicate threshold used for scaffold-based clustering [43]. Both metrics were calculated in both directions and summarized as symmetric scores: $\text {MMTS}\text {sym} = (\text {MMTS}{A\rightarrow B} + \text {MMTS}{B\rightarrow A})/2$ and $\text {LF}\text {sym} = (\text {LF}{A\rightarrow B} + \text {LF}{B\rightarrow A})/2$. Higher values for either metric indicate greater structural overlap, suggesting a higher likelihood of information leakage.

Overall, P2MAT surpassed the published model in six out of seven external comparisons, and the MMTS and LF values help clarify the observed results. On the dataset from McDonagh et al. [14] (MMTS = 0.623,  LF = 34.05%), P2MAT lowered the reported MLR error from 33.20 to 22.20 K, marking an improvement of roughly 23%. For the large MDPI dataset from Karthikeyan et al. [58] (4, 173 compounds; MMTS = 0.685, LF = 45.13%), the MAE decreased from 32.60 to 22.20 K and the RMSE from 49.30 to 35.65 K, while $R^2$ stayed similar at 0.68. On the drug-compound dataset from Modarresi et al. [59] (MMTS = 0.574, LF = 34.94%), the RMSE dropped from 42.00 to 32.08 K, and $R^2$ increased from 0.66 to 0.80. When tested on the large OCHEM dataset used by both Tetko et al. [60] and Sivaraman et al. [16] (21, 840compounds; MMTS = 0.842, LF = 66.01%), P2MAT achieved an RMSE of 35.10 K, compared to Tetko’s 38.00 K, and an $R^2$ of 0.83, compared to Sivaraman’s 0.77. The strong performance likely stems from the high overlap with our training set. P2MAT performed best on the dataset from McDonagh et al. [61] (1, 100 compounds; MMTS = 0.665, LF = 42.21%), where the RMSE improved from 41.30 to 27.43 K and $R^2$ rose from 0.75 to 0.90. Notably, this dataset is a subset of the compounds used to train P2MAT, which suggests that the high LF reflects direct overlap with the training set rather than genuine external generalization. The only exception was the dataset from Bergström et al. [62] (277 compounds; MMTS = 0.415, LF = 10.26%), which includes structurally constrained, drug-like molecules and had the least overlap among the benchmark sets. On this out-of-distribution dataset, P2MAT produced an RMSE of 48.86 K and an $R^2$ of 0.03, consistent with its limited structural similarity to our training data. Together, these results indicate that P2MAT generalizes well across chemically diverse external datasets, while instances of poorer performance relate closely to lower structural overlap with the training set.

## Discussion

The stacking ensembles achieved test MAEs of 25.3–28.7 K and $R^2$ values of 0.70–0.83. This performance puts them within the 2–35 K range reported for similar QSAR and deep-learning models predicting melting points in the literature [58, 62, 63]. Across all 12 dataset and feature-selection combinations, the stack consistently improved MAE by 2–4% compared to the best individual base model. This confirms that the Ridge meta-learner effectively uses the complementary strengths of the four base learners. The significantly lower $R^2$ for CHON (0.701) compared to CHO (0.829) indicates that nitrogen-containing compounds introduce extra structural variability. This variability includes intramolecular hydrogen bonding, tautomerism, and resonance delocalization in heterocycles that fixed 2D PaDEL descriptors cannot fully capture. Incorporating graph neural network embeddings that respond to electronic structure represents a logical way to improve further.

Together, the SHAP and applicability-domain analyses create a physically sound and practically useful uncertainty framework. SHAP rankings show that the Abraham LFER descriptors (MLFER_E, MLFER_S, and MLFER_A) are the most influential predictors across all three datasets. This supports the theoretical basis of linear free-energy relationships for melting-point prediction. In contrast, RotBFrac is the most consistent negative predictor. This indicates that molecular flexibility generally lowers the melting point by increasing the crystallization entropy penalty, regardless of elemental composition. For deployment, the Williams-plot applicability-domain boundary ($h_i > h^*$) and conformal prediction intervals provide two complementary levels of uncertainty communication. In-domain predictions are linked to a well-calibrated 90% interval of ±53 to 60 K and have an expected MAE of 23–27 K. However, out-of-domain queries (3.9% of test compounds in each dataset) must be interpreted carefully because structural extrapolation introduces an additional systematic error of about 2–3 K.

## Availability and requirements

Project name: e.g. My bioinformatics project

## Supplementary Information

Supplementary material 1.

## Data Availability

The data is available in the data folder located in the github of the project: https://github.com/sandialabs/P2MAT.

## Declarations

### Availability and requirements

• Project name : P2MAT

• Project home page : https://github.com/sandialabs/P2MAT

• Operating system(s) : MacOS, Windows, Linux

• Programming language : Python

• Other requirements : Java, XCode, and Anaconda/Miniconda

• License : BSD 3-Clause

No restriction for non-academic use.
