# Supplementary material for: P2MAT: A machine learning (ML) driven software for Property Prediction of MATerial
Source: J Cheminform. 2026 Jul 3;18:144. doi: 10.1186/s13321-026-01247-w (PMC13679253; doi:10.1186/s13321-026-01247-w)
Supplement: Supplementary file 1 [file 13321_2026_1247_MOESM1_ESM.pdf]

P2MAT: A machine learning (ML) driven software for Property Prediction of MATerial

Methun Kamruzzaman[6,7*], Alexander Landera[1], Nalini Menon[2], Mark D. Allendorf[4,5], Kunal Poorey[3*]

[1]Applied Biosciences and Engineering, Sandia National Laboratories, 7011 East Ave, Livermore, 94550, CA, USA.

[2]Materials Research, Sandia National Laboratories, 7011 East Ave, Livermore, 94550, CA, USA.

[3]Materials Aging and Reliability, Sandia National Laboratories, 7011 East Ave, Livermore, 94550, CA, USA.

[4](Former) Materials Research, Sandia National Laboratories, 7011 East Ave, Livermore, 94550, CA, USA.

[5]Honorary Sr. Visiting Scholar, Washington University in St. Louis, 1 Brookings Drive, St. Louis, 63130, MO, USA.

[6](Former) Applied Biosciences and Engineering, Sandia National Laboratories, 7011 East Ave, Livermore, 94550, CA, USA.

[7]Department of Computer Science and Data Science, Meharry Medical College, 1005 Dr DB Todd Jr Blvd, Nashville, 37208, TN, USA.

*Corresponding author(s). E-mail(s): md.kamruzzaman@mmc.edu; kpoorey@sandia.gov;

Contributing authors: alande@sandia.gov; ncmenon@sandia.gov; mdallendorf@wustl.edu;

Contents

S1 Melting point using 2D features on CHON and FULL dataset 3
- S1.1 Stack based model performance on CHO and FULL dataset 3
- S1.2 Uncertainty analysis on CHO and FULL dataset 3
- S1.3 SHAP analysis on three datasets (CHO, CHON, and FULL) 3
  - S1.3.1 Summary and Takeaways 11

S2 Melting point using 2D and 3D features 15
- S2.1 Dataset partitioning 15
- S2.2 Model Performance 16
  - S2.2.1 Individual Model Performance. 16
  - S2.2.2 Stacking Ensemble Performance. 17
- S2.3 Outlier Detection and Applicability Domain Analysis. 18
- S2.4 SHAP-Based Explanation of Stacking Ensemble Predictions. 22
- S2.5 Uncertainty Quantification of Stacking Ensemble Predictions. 23
- S2.6 Discussion 23

S3 Boiling point 28
- S3.1 Dataset partitioning 28
- S3.2 Model Performance 29
  - S3.2.1 Individual Model Performance. 29
  - S3.2.2 Stacking Ensemble Performance. 30
- S3.3 Outlier Detection and Applicability Domain Analysis. 31
- S3.4 SHAP-Based Explanation of Stacking Ensemble Predictions. 35
- S3.5 Uncertainty Quantification of Stacking Ensemble Predictions. 36
- S3.6 Discussion 37

S1 Melting point using 2D features on CHON and FULL dataset

S1.1 Stack based model performance on CHO and FULL dataset

Fig. S1 presents the experimental analysis for CHON, and Fig. S2 represents the experimental analysis of the full data.

S1.2 Uncertainty analysis on CHO and FULL dataset

Fig. S3 presents the experimental analysis for CHON, and Fig. S4 represents the experimental analysis of the full data.

S1.3 SHAP analysis on three datasets (CHO, CHON, and FULL)

Three descriptors appear in the top 15 features of *all three* datasets: `MLFER_E`, `MLFER_S`, and `RotBFrac` (Table S1). Their consistent importance suggests these features encode *universal* physicochemical principles of melting rather than dataset-specific artifacts.

Table S1: Descriptors appearing in the top-15 features of all three datasets, with their dataset-specific ranks and SHAP directions.

Feature	CHO rank	CHON rank	Full rank	Direction / physical basis
`MLFER_E`	6	1	2	Raises MP; electronic polarisability → stronger crystal dispersion and π-stacking
`MLFER_S`	9	6	1	Raises MP (predominantly); dipolarity → stronger dipole–dipole crystal contacts
`RotBFrac`	10	5	6	Lowers MP; molecular flexibility → reduced crystal-packing efficiency

Beyond these three, a second tier of features is shared between at least two datasets, reflecting the physicochemical overlap between the subsets. `ASP-1` (asphericity, lowers MP) is prominent in both CHO and CHON; `SHBd` and `nHBint2` (H-bond donor descriptors, raise MP) appear in both CHON and the full dataset; and `MLFER_A` (H-bond acidity) is top-ranked in CHO and reaches the top 10 in the full set, indicating that H-bond donor capacity becomes less discriminating as N-rich CHON compounds introduce competing acceptor sites.

Table S2 lists all 36 unique features that appear in the top-20 SHAP rankings of at least one of the three datasets (CHO, CHON, Full), together with their descriptor category, beeswarm rank in each dataset, and plain-language definition. A dash (—) indicates the feature did not rank in the top 20 for that dataset. Features are sorted by category and then alphabetically within each category.

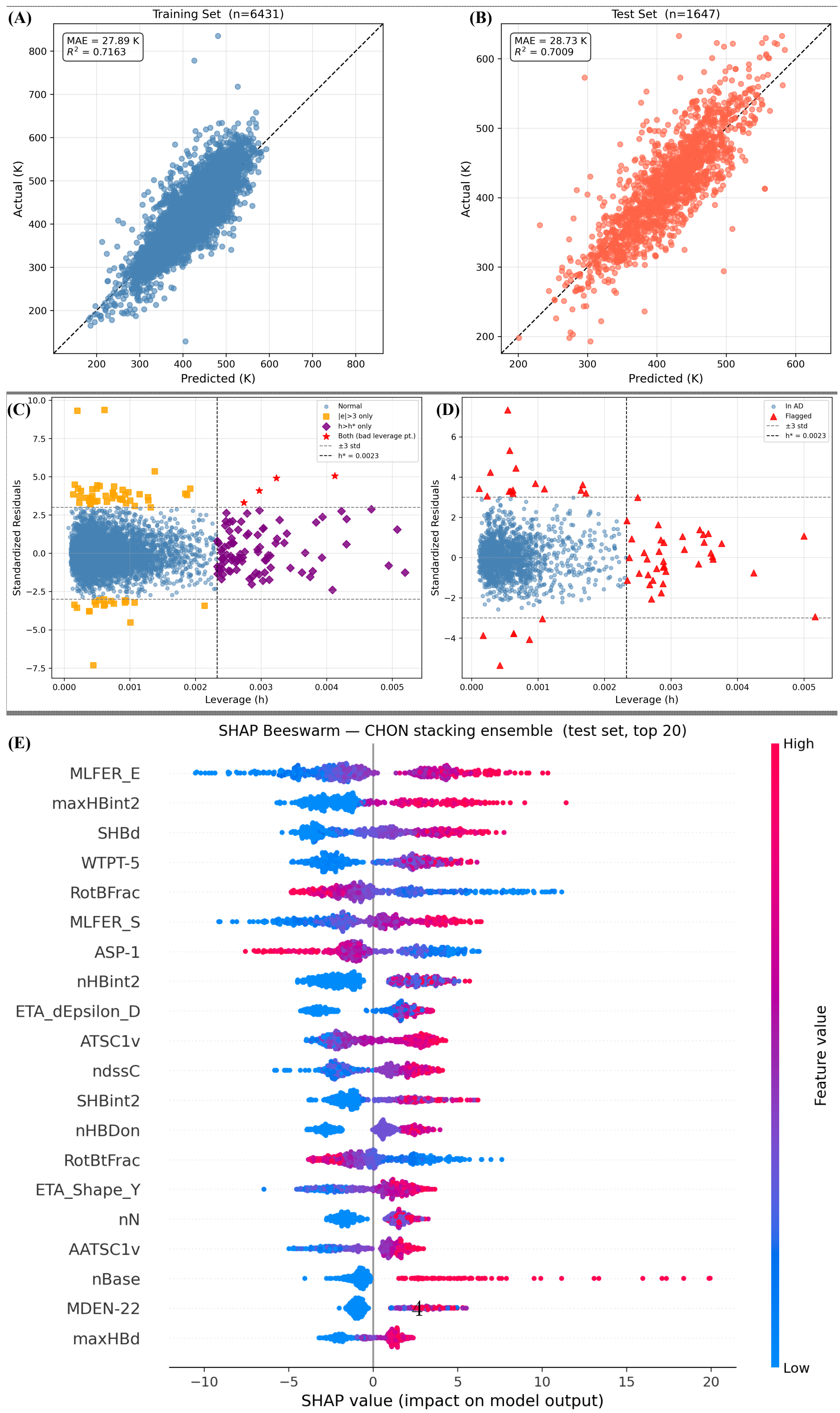

Fig. S1: **Panel (A–B)** Stacking ensemble predicted vs. experimental melting points for the **CHON** dataset (whole features, Standard Scaler). The dashed diagonal line represents perfect prediction. Color density reflects point overlap. **Panel (C–D)** Williams plot for outlier detection. Each point is a training compound plotted at its leverage h_i (x-axis) against its standardised residual ε_i (y-axis). Vertical dashed line: leverage warning threshold $h^* = 0.00233$. Horizontal dashed lines: $|\varepsilon| = 3$. Compounds in the upper/lower region ($|\varepsilon| > 3$) are response outliers; those to the right of h^* are leverage outliers; those satisfying both criteria are bad leverage points. **Panel (E)** SHAP beeswarm plot for the **CHON** dataset stacking ensemble (500 test compounds, whole features, Quantile Transformer). `MLFER_E` (Abraham excess molar refraction) dominates with $\overline{|\text{SHAP}|} = 3.6\,\text{K}$ and a strongly positive monotone direction ($r = +0.91$). Hydrogen-bond descriptors (`maxHBint2`, `SHBd`, rank 2–3) emerge prominently due to the introduction of nitrogen-containing donors and acceptors.

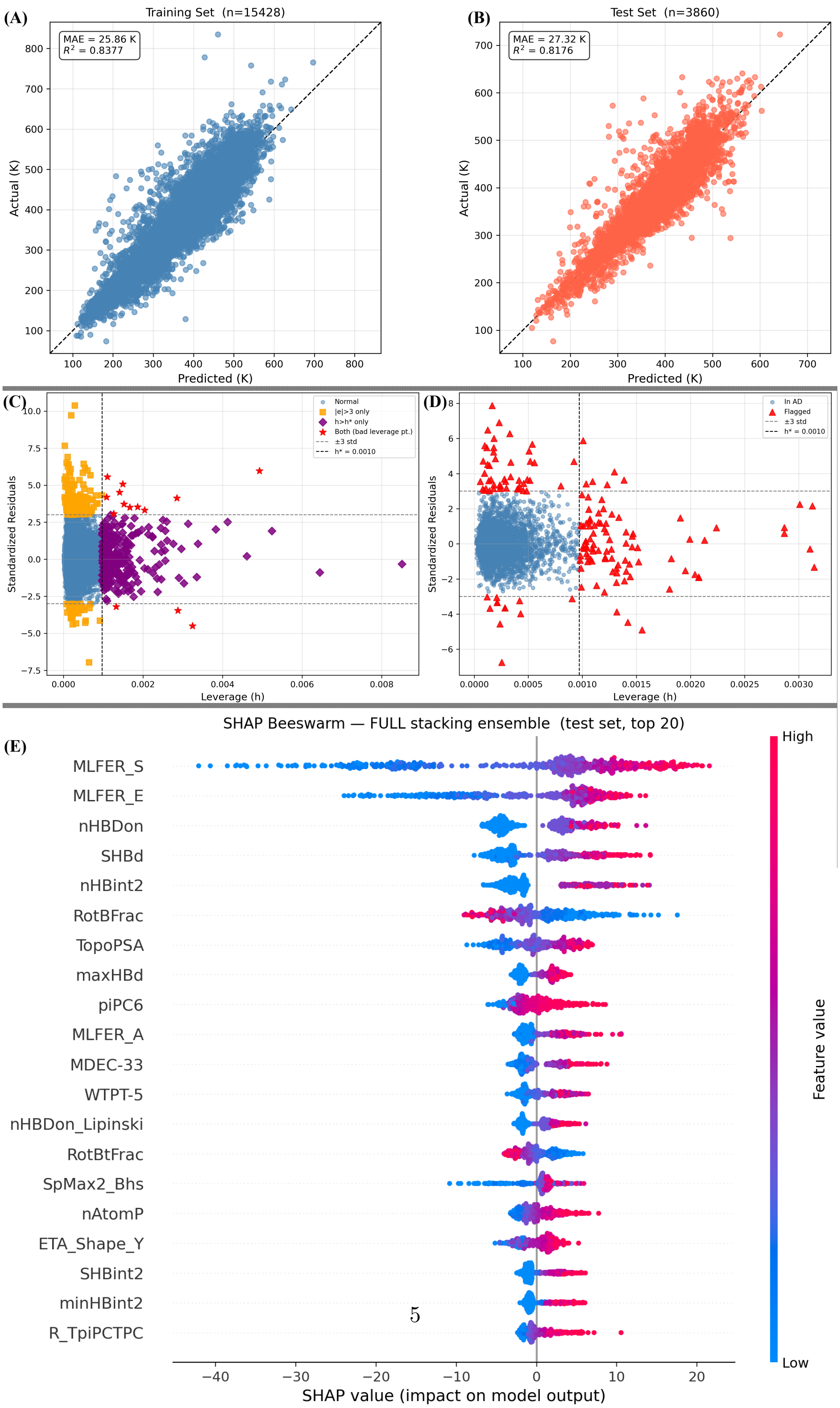

Fig. S2: **Panel (A–B)** Stacking ensemble predicted vs. experimental melting points for the **FULL** dataset (whole features, Standard Scaler). The dashed diagonal line represents perfect prediction. Color density reflects point overlap. **Panel (C–D)** Williams plot for outlier detection. Each point is a training compound plotted at its leverage h_i (x-axis) against its standardised residual ε_i (y-axis). Vertical dashed line: leverage warning threshold $h^* = 0.00097$. Horizontal dashed lines: $|\varepsilon| = 3$. Compounds in the upper/lower region ($|\varepsilon| > 3$) are response outliers; those to the right of h^* are leverage outliers; those satisfying both criteria are bad leverage points. **Panel (E)** SHAP beeswarm plot for the **Full** dataset stacking ensemble (500 test compounds, whole features, Standard Scaler). `MLFER_S` (Abraham dipolarity/polarisability) is the dominant feature with $\overline{|\mathrm{SHAP}|} = 11.4\,\mathrm{K}$ and a SHAP range of -42 to $+22\,\mathrm{K}$, reflecting the extreme polarity contrast between ionic/zwitterionic compounds and nonpolar hydrocarbons in the full chemical space.

Table S2: Definitions of all features appearing in the top-20 SHAP beeswarm rankings for the CHO, CHON, and Full melting-point datasets. Ranks are by mean absolute SHAP value; — = not in top 20.

**Feature**	**Category**	**CHO**	**CHON**	**Full**	**Definition**
Abraham Linear Free Energy Relationship (LFER) parameters					
MLFER_A	LFER	7	—	10	Abraham hydrogen-bond acidity ($\mathcal{A}$): measures the compound's capacity to donate a hydrogen bond; high values indicate strong H-bond donors (phenols, carboxylic acids, primary amides).
MLFER_E	LFER	6	1	2	Abraham excess molar refraction ($\mathcal{E}$): electronic polarisability above that of a saturated alkane of the same size; reflects contributions from π-electrons, lone pairs, and heavy halogens.
MLFER_S	LFER	9	6	1	Abraham dipolarity/polarisability ($\mathcal{S}$): encodes dipole–dipole and dipole–induced-dipole interactions; high values correspond to highly polar functional groups such as nitro, sulfonyl, and carbonyl.
Three-dimensional shape descriptors					
ASP-1	3-D shape	2	7	—	Asphericity (1st component): measures deviation of the molecular shape from a perfect sphere based on the principal moments of inertia; 0 = globular, large values = elongated or disc-like.
ASP-2	3-D shape	17	—	—	Asphericity (2nd component): complementary moment-of-inertia ratio capturing anisotropy in the plane perpendicular to the longest molecular axis.
ETA_Shape_Y	3-D shape	—	15	17	ETA shape descriptor Y: quantifies three-dimensional molecular anisotropy via the Extended Topochemical Atom (ETA) formalism using atomic contributions weighted by bond orders.
Autocorrelation descriptors (topological)					

Continued on next page

Table S2 *(continued)*

Feature	Category	CHO	CHON	Full	Definition
AATSC1v	Autocorrelation	11	17	—	Centred lag-1 Broto–Moreau autocorrelation of van der Waals volumes: mean-centred version of ATSC1v; encodes relative deviation of adjacent atom sizes from the molecular average.
AATS2v	Autocorrelation	5	—	—	Averaged lag-2 Moreau–Broto autocorrelation of van der Waals volumes: encodes average pairwise similarity of van der Waals volumes for atoms separated by two bonds (next-nearest neighbours).
ATSC1v	Autocorrelation	3	10	—	Lag-1 Broto–Moreau autocorrelation of van der Waals volumes: captures how similar adjacent (one-bond separated) atoms are in terms of atomic volume; high values indicate uniformly bulky bonded pairs.
MATS1v	Autocorrelation	8	—	—	Moran autocorrelation lag-1 of van der Waals volumes: mean-normalised lag-1 autocorrelation; positive values indicate that adjacent atoms are more similar in size than the molecular average, negative values indicate alternation.
Hydrogen-bond descriptors					
SHBd	H-bond	—	3	4	Sum of H-bond donor E-state indices: aggregates the electrotopological state index (E-state) over all hydrogen-bond donor atoms; simultaneously encodes the number and electronic strength of donors.
SHBint2	H-bond	12	12	18	Sum of intramolecular H-bond descriptors at 2-bond separation: aggregates energetic contributions of all intramolecular hydrogen bonds where donor and acceptor are separated by exactly two intervening bonds.

Continued on next page

Table S2 *(continued)*

Feature	Category	CHO	CHON	Full	Definition
SsOH	H-bond	19	—	—	Sum of E-state indices for hydroxyl (–OH) groups: electrotopological state index summed over all oxygen atoms in –OH environments; encodes both the number and electronic richness of hydroxyl donors.
maxHBd	H-bond	—	20	8	Maximum H-bond donor E-state value: the electrotopological state index of the single strongest H-bond donor atom in the molecule; captures the presence of unusually active donor sites.
maxHBint2	H-bond	20	2	—	Maximum intramolecular H-bond descriptor at 2-bond separation: strength of the strongest intramolecular hydrogen bond where donor and acceptor are two bonds apart; a proxy for electron-rich heteroatom arrangements.
minHBint2	H-bond	18	—	19	Minimum intramolecular H-bond descriptor at 2-bond separation: the weakest 2-bond intramolecular H-bond in the molecule; non-zero values confirm the presence of at least one such interaction.
nHBDon	H-bond	—	13	3	Count of hydrogen-bond donor atoms: total number of N–H and O–H groups; a simple, interpretable count that captures the gross H-bonding capacity of the molecule.
nHBDon_Lipinski	H-bond	—	—	13	Hydrogen-bond donor count (Lipinski definition): number of –NH and –OH groups counted according to Lipinski's original rule-of-five convention; equivalent to `nHBDon` but using a stricter atom-type filter.
nHBint2	H-bond	—	8	5	Count of intramolecular hydrogen bonds at 2-bond separation: number of donor–acceptor pairs separated by exactly two intervening bonds; indicates the degree of intramolecular H-bond network formation.
Topological and graph-theoretical descriptors					

Continued on next page

Table S2 *(continued)*

Feature	Category	CHO	CHON	Full	Definition
JGI4	Topological	16	—	—	Mean topological charge index of order 4: captures the mean charge transfer over paths of length 4 in the molecular graph, encoding medium-range electronic communication through the bond network.
MDEC-33	Topological	4	—	11	Molecular Distance Edge descriptor for C3–C3 pairs: encodes the frequency and spatial distribution of trisubstituted (sp^3) carbon–carbon contacts; high values reflect extensively branched frameworks with reduced conformational freedom.
MDEN-22	Topological	—	19	—	Molecular Distance Edge descriptor for N2–N2 pairs: encodes the separation and abundance of disubstituted nitrogen–nitrogen contacts in the molecular graph; relevant for di-amine and hydrazone-type structures.
MDEO-11	Topological	1	—	—	11th eigenvalue of the Modified Detour matrix: topological–electronic descriptor that encodes global branching and ring-closure patterns of the molecular graph weighted by detour (longest non-revisiting) distances; sensitive to planarity and cyclicity.
R_TpiPCTPC	Topological	—	—	20	Ratio of π-path connectivity to total path connectivity: measures the fractional contribution of π-bond paths to the total topological connectivity of the molecule; high values indicate π-electron-rich frameworks.
SpMax2_Bhs	Topological	—	—	15	2nd largest eigenvalue of the Burden matrix (Sanderson electronegativity weights, H-suppressed): encodes electronic and topological structure via the second spectral moment of the bond-connectivity matrix weighted by relative atomic electronegativities.

Continued on next page

Table S2 *(continued)*

Feature	Category	CHO	CHON	Full	Definition
WTPT-5	Topological	—	4	12	Wiener-type path total descriptor of order 5: sum of all molecular path lengths of exactly five bonds; captures topological size and branching complexity at the medium-range (5-bond) scale, beyond what simple atom counts provide.
piPC6	Topological	14	—	9	π-component of the 6th-order path connectivity index: encodes the contribution of π-bonds to topological connectivity over paths of length 6; high values indicate extended conjugated or aromatic systems.
Count and fraction descriptors					
ETA_dEpsilon_D	ETA	—	9	—	Extended Topochemical Atom $\delta\varepsilon_D$ descriptor: quantifies the change in local electron density in the ETA framework; sensitive to the presence of lone-pair-donating heteroatoms and their immediate bonding environment.
nAtomLC	Count	15	—	—	Number of atoms in the largest ring system: count of heavy atoms belonging to the single largest fused or bridged polycyclic ring assembly; correlates with aromatic-core size and crystal-packing planarity.
nAtomP	Count	—	—	16	Number of phosphorus atoms: count of all P atoms in the molecule; nonzero values flag phosphate, phosphonate, and phosphine-containing compounds, which typically display elevated melting points due to strong ionic or coordination interactions.
nBase	Count	—	18	—	Number of basic groups: count of proton-accepting (basic) functional groups; includes aliphatic and aromatic amines and other Lewis-basic heteroatom-containing moieties.

Continued on next page

Table S2 *(continued)*

Feature	Category	CHO	CHON	Full	Definition
nN	Count	—	16	—	Number of nitrogen atoms: total count of all N atoms; a simple elemental descriptor that serves as a proxy for hydrogen-bond acceptor and donor capacity in CHON chemical space.
ndssC	Count	13	11	—	Number of sp^2 carbon atoms of type >C=: count of carbon atoms that carry exactly two single bonds and one double bond (i.e., carbonyl, imine, and vinyl carbons); correlates with the degree of unsaturation and molecular rigidity.
RotBFrac	Count	10	5	6	Fraction of rotatable bonds: ratio of rotatable bonds to the total number of heavy-atom bonds; the single most consistent *negative* predictor of melting point in this study — more flexible molecules face a greater entropic penalty upon crystallisation.
RotBtFrac	Count	—	14	14	Fraction of rotatable bonds (total, including terminal bonds): similar to `RotBFrac` but includes terminal single bonds (e.g., methyl, halomethyl) in the denominator; provides a complementary flexibility measure with a stricter bond-counting convention.
TopoPSA	Count	—	—	7	Topological Polar Surface Area: sum of surface-area contributions from polar atoms (O, N, S, P) and their attached hydrogens, computed from a lookup table of fragment contributions; captures the molecule's polar surface independently of 3-D geometry.

S1.3.1 Summary and Takeaways

SHAP analysis offers a clear and chemical view of melting-point predictions across three molecular databases.

1. **Polarisability and dipolarity stand out as the most consistent drivers.** The features `MLFER_E` and `MLFER_S`, derived from Abraham's Linear Free Energy Relationship, rank among the most influential in all datasets. Higher polarisability

and dipolarity strengthen intermolecular attractive forces within the crystal lattice. This increase raises the energy (and the temperature) needed to disrupt the lattice.

2. **Hydrogen-bonding capacity is the next most important factor, especially in nitrogen-containing compounds.** Descriptors like `MLFER_A`, `SHBd`, `nHBDon`, and `maxHBint2` reflect a molecule's ability to donate and maintain directional hydrogen bonds. Their significance in the CHON and full datasets aligns with the known role of hydrogen bonding in raising melting points compared to non-hydrogen-bonding counterparts of similar size.
3. **Molecular flexibility tends to lower melting points.** The descriptor `RotBFrac` shows a consistently negative relationship across all three datasets. This trend likely comes from the larger entropic penalty that flexible molecules face during crystallization. This situation decreases the free-energy difference between the solid and liquid states, which lowers the melting temperature. This supports the long-held view in medicinal chemistry that greater rigidity is linked to higher melting points.
4. **For CHO compounds, topological descriptors specific to the dataset become especially important.** Descriptors like `MDEO-11`, `ATSC1v`, `MDEC-33`, and `AATS2v` stand out in the CHO dataset, while they are less significant in the CHON and full datasets. This suggests that, in a limited C/H/O environment, subtle changes in molecular topology and local atomic volume distribution become key once broader factors such as MLFER descriptors and hydrogen-bond counts are already partly controlled.
5. **Finally, the beeswarm plots show noticeable nonlinearity and specificity by compound class.** Features such as `nHBint2` and `MDEO-11` exhibit mixed-color swarms. The SHAP values in these features span both positive and negative directions over similar feature-value ranges. This indicates context-dependent effects influenced by interactions with other structural traits.

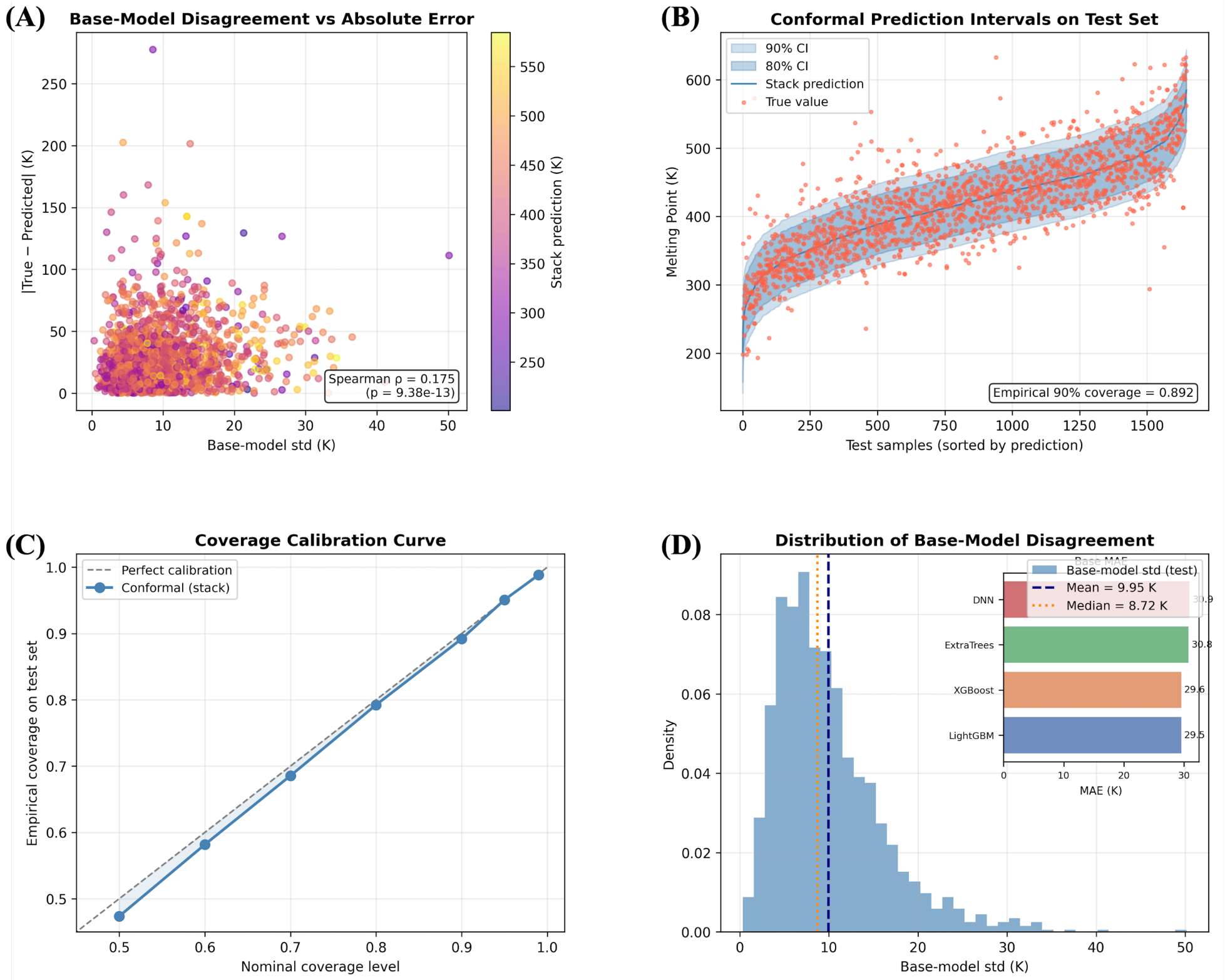

Fig. S3: Four-panel uncertainty analysis for the **CHON** dataset stacking ensemble (whole features, Quantile Transformer; $n_{\text{test}} = 1{,}647$). **Panel (A)** Scatter plot of the base-model standard deviation against the absolute prediction error for each test compound, coloured by stack prediction. The Spearman correlation ($\rho = 0.175$, $p < 0.0001$) is lower than for CHO, consistent with the higher structural diversity of nitrogen-containing chemistry reducing the coherence of inter-model disagreement as an error proxy. **Panel (B)** Sorted test compounds with 80% and 90% conformal bands, stack predictions, and true values overlaid. The conformal quantile $q_{90} = \pm 59.6\,\text{K}$ is wider than for CHO, reflecting the larger intrinsic spread of the CHON melting-point distribution. Empirical 90% coverage is 89.2%. **Panel (C)** Nominal vs. empirical coverage across seven levels (50%–99%). The curve closely hugs the perfect-calibration diagonal, indicating that the distribution-free conformal guarantee is maintained despite the Quantile Transformer applied to the target variable. **Panel (D)** Density histogram of base-model standard deviation (mean = $9.95\,\text{K}$), with mean and median marked. The inset bar chart shows the test MAE of each individual base model; the DNN exhibits a notably larger MAE relative to the tree-based learners under the Quantile Transformer, contributing to the higher mean disagreement compared with the CHO ensemble.

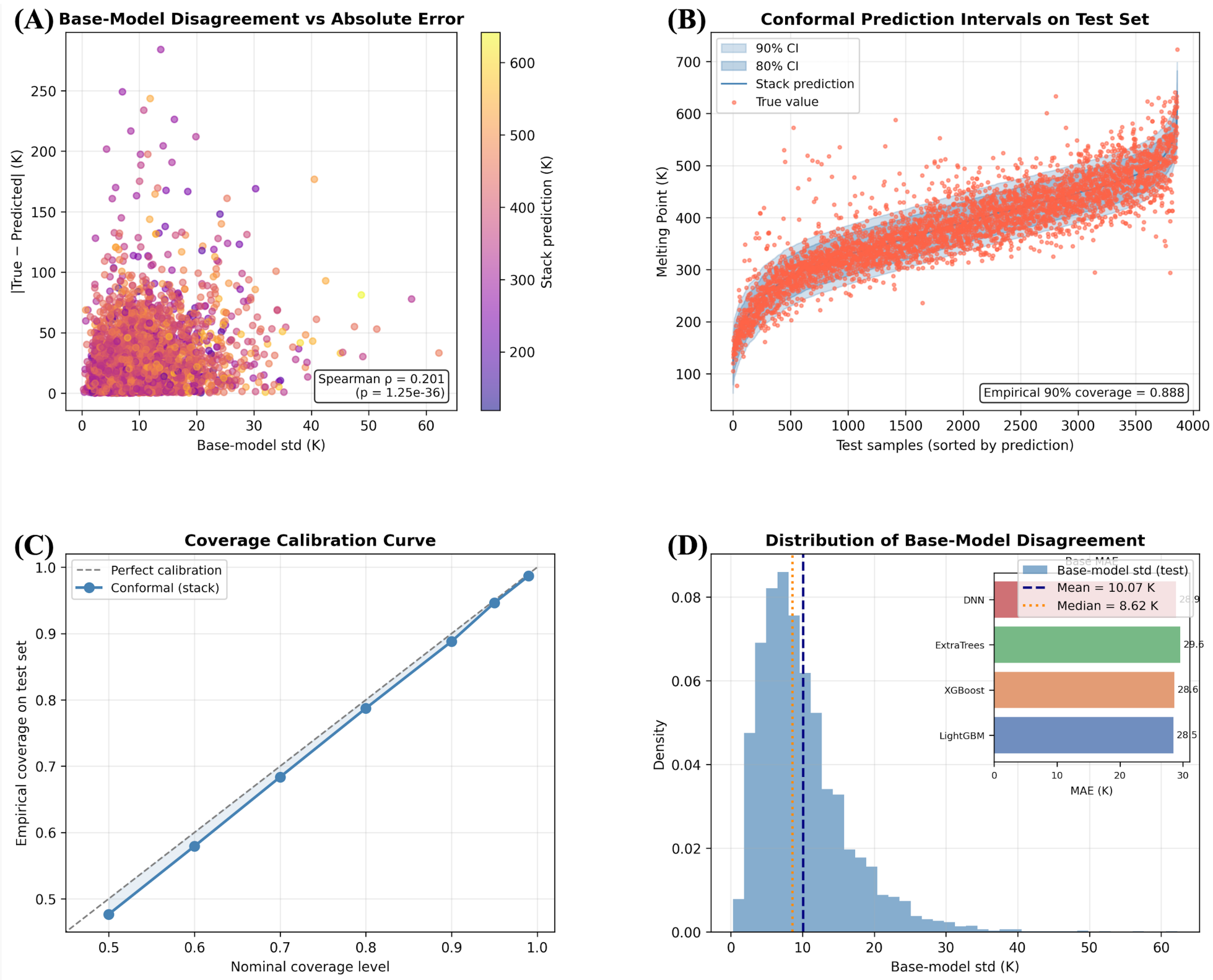

Fig. S4: Four-panel uncertainty analysis for the **Full** dataset stacking ensemble (whole features, Standard Scaler; $n_{\text{test}} = 3{,}860$). **Panel (A)** Scatter of base-model standard deviation against absolute prediction error, coloured by stack prediction (plasma scale). The Spearman correlation ($\rho = 0.201$, $p < 0.0001$) is intermediate between CHO and CHON; the larger sample size increases statistical power while the wider chemical space (halogenated, sulfur-, and phosphorus-containing compounds) broadens the prediction spread. **Panel (B)** Test compounds ($n = 3{,}860$) sorted by increasing stack prediction, overlaid with the 80% and 90% symmetric conformal bands, the stack prediction line, and experimental values. The conformal quantile is $q_{90} = \pm 56.5\,\text{K}$; the empirical 90% coverage of 88.8% deviates from the nominal target by only 1.2 percentage points, confirming calibration at scale. **Panel (C)** Nominal vs. empirical coverage at seven levels (50%–99%) for the Full ensemble. The conformal curve tracks the perfect-calibration diagonal across the entire range, demonstrating that the split-conformal approach is robust to the heterogeneous composition of the full chemical space. **Panel (D)** Density histogram of base-model standard deviation across all 3,860 test compounds (mean $= 10.08\,\text{K}$), with mean and median lines. The inset bar chart displays the per-base-model test MAE; LightGBM and XGBoost achieve the lowest individual errors, consistent with their dominant SHAP contributions and the highest meta-learner coefficients in the Ridge stacking model.

S2 Melting point using 2D and 3D features

This section presents the full evaluation of the QSAR melting-point prediction pipeline across three chemical-space datasets (CHO, CHON, and Full), extended to incorporate 3D conformer-derived molecular descriptors (RDF, WHIM, GETAWAY, and related 3D autocorrelation descriptors) alongside the standard 2D PaDEL descriptor set. We first describe the dataset composition and partitioning strategy, then report the performance of six individual base learners across three data-transformation strategies (no transformation, Standard Scaler, and Quantile Transformer), followed by the stacking ensemble that combines the strongest base models via a Ridge meta-learner. Applicability-domain analysis using Williams plots identifies response and leverage outliers in the training data and establish per-compound confidence boundaries for prospective predictions. Prediction accuracy is visualized through hexbin plots of predicted versus experimental melting points, and SHAP beeswarm plots provide a feature-level interpretation of the ensemble's decisions. The section concludes with an uncertainty quantification framework and a discussion of the results in the context of the broader QSAR literature.

S2.1 Dataset partitioning

The dataset was split into 3589 training and 897 test compounds using Butina clustering with a Tanimoto similarity threshold of 0.8. Within the training set, the mean pairwise Tanimoto similarity was 0.094, with a median of 0.086, whereas in the test set, the corresponding values were 0.092 and 0.083, respectively. Across both sets, the mean maximum similarity between each test compound and its nearest training neighbor was 0.514, with a median of 0.524 and a maximum of 0.796. No test compound had a nearest-neighbor similarity equal to or greater than the 0.8 threshold. These results confirm that the cluster-based split effectively minimizes structural overlap between the training and test sets, reducing the risk of information leakage in downstream model evaluation.

Table S3: Tanimoto similarity statistics for the training and test sets, together with the maximum similarity of each test compound to its nearest neighbor in the training set (Cross). The low mean and median similarities within and between sets, along with the absence of test compounds at or above the 0.8 threshold, confirms that the cluster-based split effectively minimizes structural overlap.

Metric	Train	Test	Cross
Compounds	3589	897	—
Mean similarity	0.094	0.092	0.514
Median similarity	0.086	0.083	0.524
Max similarity	0.640	0.824	0.796
% pairs $\geq$ threshold (0.8)	0.0%	0.0%	0.0%

Three non-overlapping datasets were constructed from the curated experimental melting-point database to evaluate model performance across chemical spaces of increasing breadth. Table S4 summarises the resulting sizes. Note that the 3D descriptor extension requires a successful 3D conformer generation, which reduces dataset sizes compared to the 2D-only pipeline: compounds that failed geometry optimization or produced low-quality three-dimensional structures were excluded.

Table S4: Data demography for the three datasets used in melting-point prediction with 3D descriptors. All counts refer to unique compounds after curation, duplicate removal, and successful 3D conformer generation.

**Dataset**	n_{Total}	n_{Train} (80%)	n_{Test} (20%)
CHO	1,528	1,231	297
CHON	1,955	1,562	393
Full	4,486	3,589	897

The Full dataset is approximately 2.3 times larger than CHON and 2.9 times larger than CHO, reflecting the breadth of the complete experimental corpus after 3D filtering. The CHO and CHON subsets share a subset of compounds, so model performance differences between them isolate the effect of including nitrogen-containing chemistry rather than changes in dataset size alone.

S2.2 Model Performance

S2.2.1 Individual Model Performance.

Prior to stacking, six base learners were trained independently across all combinations of three datasets and three data-transformation strategies (no transformation, Standard Scaler, and Quantile Transformer), using the complete 3D+2D descriptor set with the whole feature set. Table S6 reports the test mean absolute error (MAE, in K) for each combination.

Several patterns emerge consistently across all three datasets. Data transformation has a decisive effect on linear model and deep neural network (DNN) performance. Without scaling, linear regression on the CHO dataset yields a dramatically inflated test MAE of 53.78 K — a consequence of the wide dynamic range of 3D descriptors such as RDF moments and WHIM eigenvalues — while both Standard Scaler and Quantile Transformer reduce this to approximately 31–32 K. The DNN also benefits substantially from scaling: on CHO with Standard Scaler it achieves a test MAE of 26.43 K, the best result among all individual models across all three datasets, whereas without scaling its MAE degrades to 49.83 K. This sensitivity likely reflects the DNN's requirement for a normalised, well-conditioned input space, which is particularly important when 2D and 3D descriptors with very different numerical ranges are concatenated. Gradient-boosted tree ensembles (LightGBM and XGBoost) are largely insensitive to the transformation strategy, achieving test MAEs of 29–34 K across all conditions, consistent with the

scale-invariance of split-based algorithms. ExtraTrees and the linear models occupy intermediate positions: ExtraTrees is unaffected by transformation, while linear models improve by 3–5 K upon scaling.

S2.2.2 Stacking Ensemble Performance.

A stacking ensemble was constructed by training a Ridge meta-learner on the out-of-fold (OOF) predictions of the four strongest base models: LightGBM, XGBoost, ExtraTrees, and the DNN. Table S5 reports the train and test performance of the stacking ensemble on each dataset using Standard Scaler and the whole feature set (no outlier removal), and Table S6 extends the comparison to include all feature-selection strategies.

Table S5: Stacking ensemble performance on the three datasets (Standard Scaler, whole feature set, no outlier removal). Train metrics are computed on the in-sample OOF predictions: test metrics are on the held-out 20% test set.

Dataset	Training performance			Testing performance		
	MAE(K)	R^2	RMSE(K)	MAE(K)	R^2	RMSE(K)
CHO	28.15	0.7787	38.87	26.29	0.8314	35.74
CHON	31.17	0.6483	41.31	32.31	0.5971	45.20
Full	29.57	0.7893	40.23	29.18	0.8030	40.49

The stacking ensemble reduces test MAE compared to the best individual model (Standard Scaler, whole feature set) in every dataset: from 26.43 to 26.29 K (−0.5%) on CHO, from 33.38 to 32.33 K (−3.1%) on CHON, and from 29.56 to 29.18 K (−1.3%) on Full. The modest gains on CHO and Full largely reflect the competitive performance of the DNN with Standard Scaler, which already approaches the stack's accuracy as an individual learner. Across all feature-selection strategies and transformations, the best stacking configurations achieve 26.29 K on CHO (Standard Scaler, whole features), 31.82 K on CHON (Quantile Transformer, correlation features), and 28.99 K on Full (Quantile Transformer, whole features). The CHO dataset achieves the highest R^2 (0.831 on the test set), reflecting the relatively narrow and homogeneous chemical space. CHON obtains a lower R^2 of 0.597, attributable to the broader structural diversity introduced by nitrogen-containing functional groups and the complex intermolecular interactions (e.g., intramolecular hydrogen bonding, resonance stabilisation of amides) that are harder to capture even with 3D descriptors. The Full dataset falls between the two ($R^2 = 0.803$), benefiting from the larger training set while confronting the widest chemical diversity.

Across all 12 datasets–feature-selection combinations, the stacking ensemble ranks first without exception, confirming the robustness of the meta-learning approach. Notably, the stacking ensemble achieves its largest recovery over the individual models under PCA feature selection (e.g., 31.41 vs. 32.49 K for DNN on Full; 29.18 vs. 30.26 K for DNN on CHO), suggesting that the meta-learner is effective at recovering the predictive

Table S6: Test MAE (K) of all models including the stacking ensemble, across datasets and feature-selection strategies (Standard Scaler). Bold entries mark the lowest MAE for each row (best model per dataset–feature-selection combination).

Dataset	FS	LR	E.Net	E.Trees	LGBM	XGB	DNN	Stack
CHO	Whole	31.99	31.18	30.40	29.81	29.37	26.43	**26.29**
	Boruta	34.06	34.03	31.39	31.34	30.44	29.25	**28.74**
	Correlation	32.62	32.12	30.98	29.80	29.08	27.84	**26.65**
	PCA	34.60	34.68	39.09	34.15	34.54	30.26	**29.18**
CHON	Whole	33.94	33.78	35.36	33.41	33.38	34.54	**32.33**
	Boruta	38.81	38.78	35.59	34.61	34.32	34.09	**33.36**
	Correlation	35.04	35.65	34.77	32.35	33.26	35.23	**32.31**
	PCA	36.98	37.04	39.66	37.45	36.58	36.67	**34.89**
Full	Whole	32.32	32.46	32.55	30.65	30.97	29.56	**29.18**
	Boruta	35.98	35.98	32.70	31.02	31.18	30.04	**29.34**
	Correlation	33.08	33.18	32.70	30.88	30.83	30.30	**29.04**
	PCA	35.51	35.53	39.18	34.84	34.72	32.49	**31.41**

FS = feature selection
LR = Linear regression
E.Net = Elastic Net regression
E.Trees = Extremely Randomized Trees Regressor
LGBM = Light Gradient Boosting Machines
XGB = Extreme Gradient Boosting
DNN = Deep Neural Network

signal when individual base models operate on a compressed, information-lossy input space. Correlation-based feature selection consistently produces competitive stacking results close to the whole-feature-set performance, indicating that retaining a curated subset of non-redundant 3D+2D descriptors is sufficient for the meta-learner.

Fig. S5–S7 represents scatter plot between real and predicted values of train and test data, outlier analysis using the Williams plot, and the SHAP-based model explainability for three datasets.

S2.3 Outlier Detection and Applicability Domain Analysis.

Williams' plot analysis was applied to the three training datasets to characterize the applicability domain (AD) of the stacking ensemble and to identify two distinct types of problematic training compounds: response outliers and leverage outliers. The leverage warning threshold was computed as $h^* = 3(p+1)/n$, where $p = 4$ is the number of base-model predictions used by the meta-learner, and n is the training set size. Compounds exceeding this threshold are structurally remote from the training centroid (high-leverage or "pure leverage" points); compounds whose standardised residual $|\varepsilon_i| > 3$ are response outliers (large prediction error). Table S7 summarises the AD statistics for all three datasets. For the CHO dataset, the h^* threshold was 0.01218, and 8 response outliers, 32 pure leverage outliers, and 0 bad leverage points were identified in the training set. For CHON, 5 response outliers, 54 pure leverage outliers, and 2

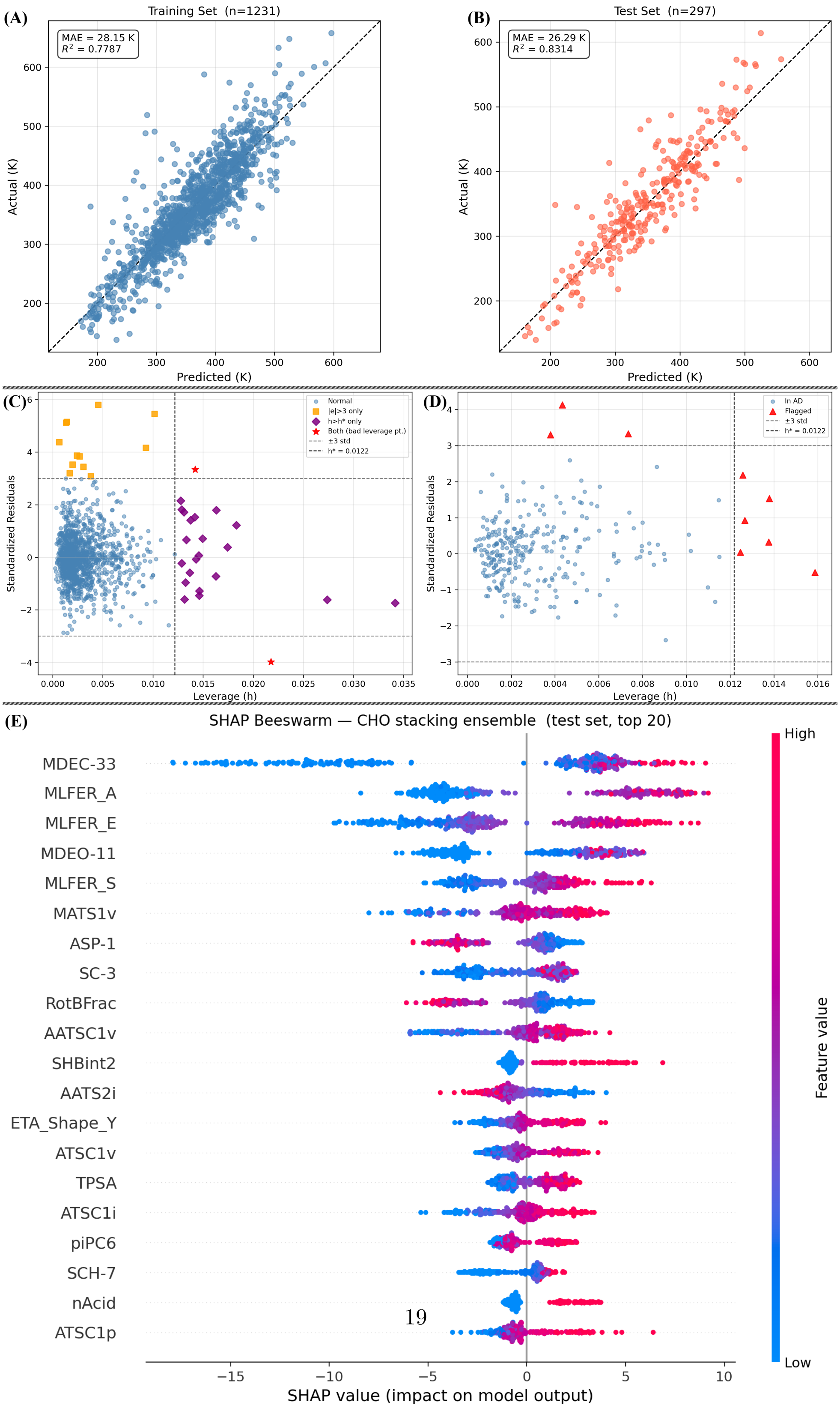

Fig. S5: **Panel (A–B)** Stacking ensemble predicted vs. experimental melting points for the **CHO** dataset (whole features, Standard Scaler). The dashed diagonal line represents perfect prediction. Color density reflects point overlap. **Panel (C–D)** Williams plot for outlier detection. Each point is a training compound plotted at its leverage h_i (x-axis) against its standardised residual ε_i (y-axis). Vertical dashed line: leverage warning threshold $h^* = 0.01218$. Horizontal dashed lines: $|\varepsilon| = 3$. Compounds in the upper/lower region ($|\varepsilon| > 3$) are response outliers; those to the right of h^* are leverage outliers; those satisfying both criteria are bad leverage points. **Panel (E)** SHAP beeswarm plot for the **CHO** dataset stacking ensemble (297 test compounds, whole features, Standard Scaler). Features ranked by mean |SHAP| (K). The top-ranked feature `MDEC-33` (C3–C3 molecular distance edge descriptor, $\overline{|\mathrm{SHAP}|} = 6.5\,\mathrm{K}$) dominates with a wide right-leaning positive spread, rising from rank 4 in the 2D model; `ASP-1` (asphericity, rank 7) shows a clean negative monotone relationship (red left, blue right), consistent with the 2D analysis.

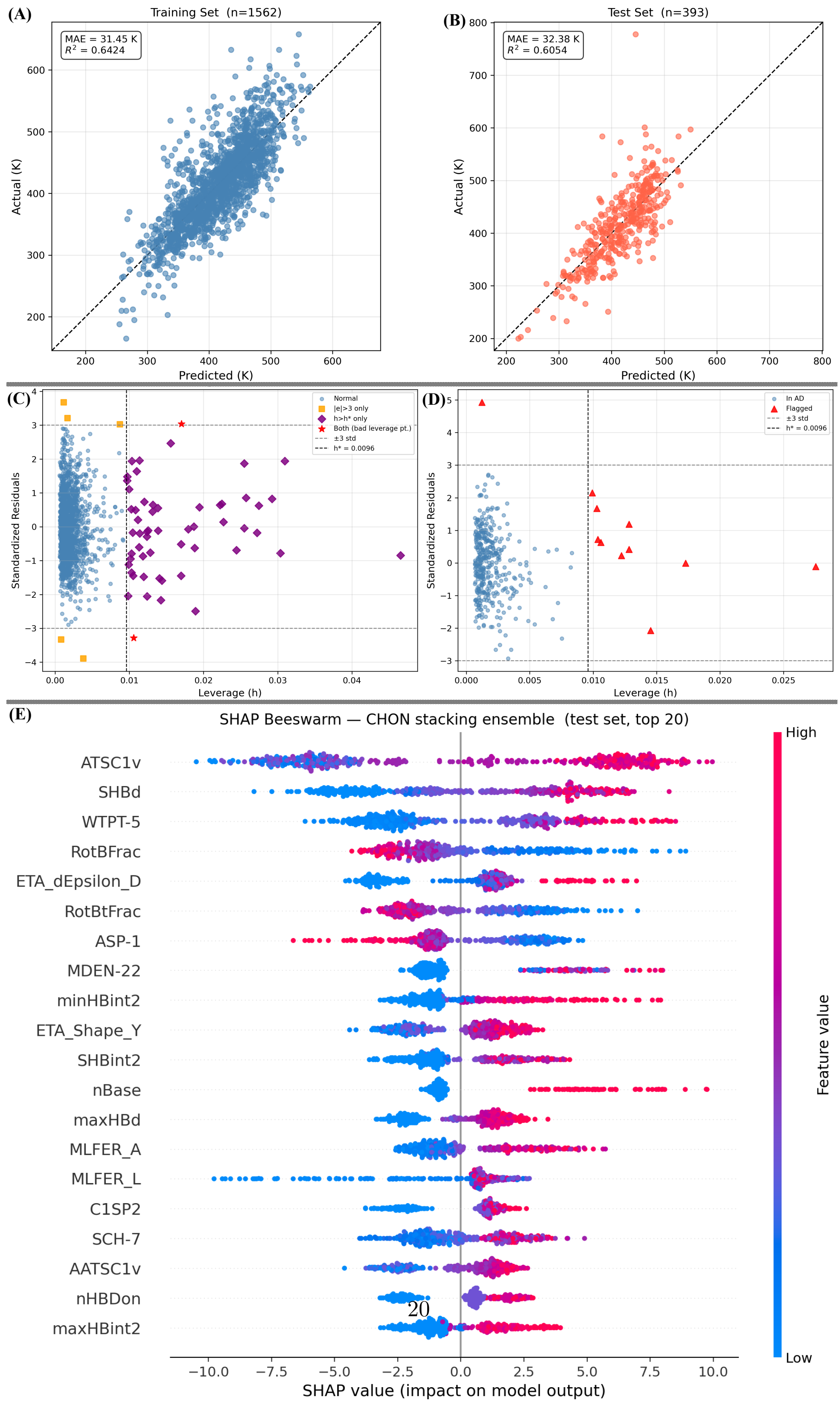

Fig. S6: **Panel (A–B)** Stacking ensemble predicted vs. experimental melting points for the **CHON** dataset (whole features, Standard Scaler). The dashed diagonal line represents perfect prediction. Color density reflects point overlap. **Panel (C–D)** Williams plot for outlier detection. Each point is a training compound plotted at its leverage h_i (x-axis) against its standardised residual ε_i (y-axis). Vertical dashed line: leverage warning threshold $h^* = 0.00960$. Horizontal dashed lines: $|\varepsilon| = 3$. Compounds in the upper/lower region ($|\varepsilon| > 3$) are response outliers; those to the right of h^* are leverage outliers; those satisfying both criteria are bad leverage points. **Panel (E)** SHAP beeswarm plot for the **CHON** dataset stacking ensemble (393 test compounds, whole features, Quantile Transformer). `ATSC1v` (lag-1 vdW volume autocorrelation) is the dominant feature with $\overline{|\text{SHAP}|} = 5.7\,\text{K}$ and a strongly positive monotone direction, rising from rank 10 in the 2D CHON model. Strikingly, `MLFER_E` — the top-ranked feature in 2D CHON — does not appear among the top 20 features in the 3D model, suggesting that the expanded 3D descriptor pool provides alternative representations of polarisability that reduce `MLFER_E`'s unique contribution.

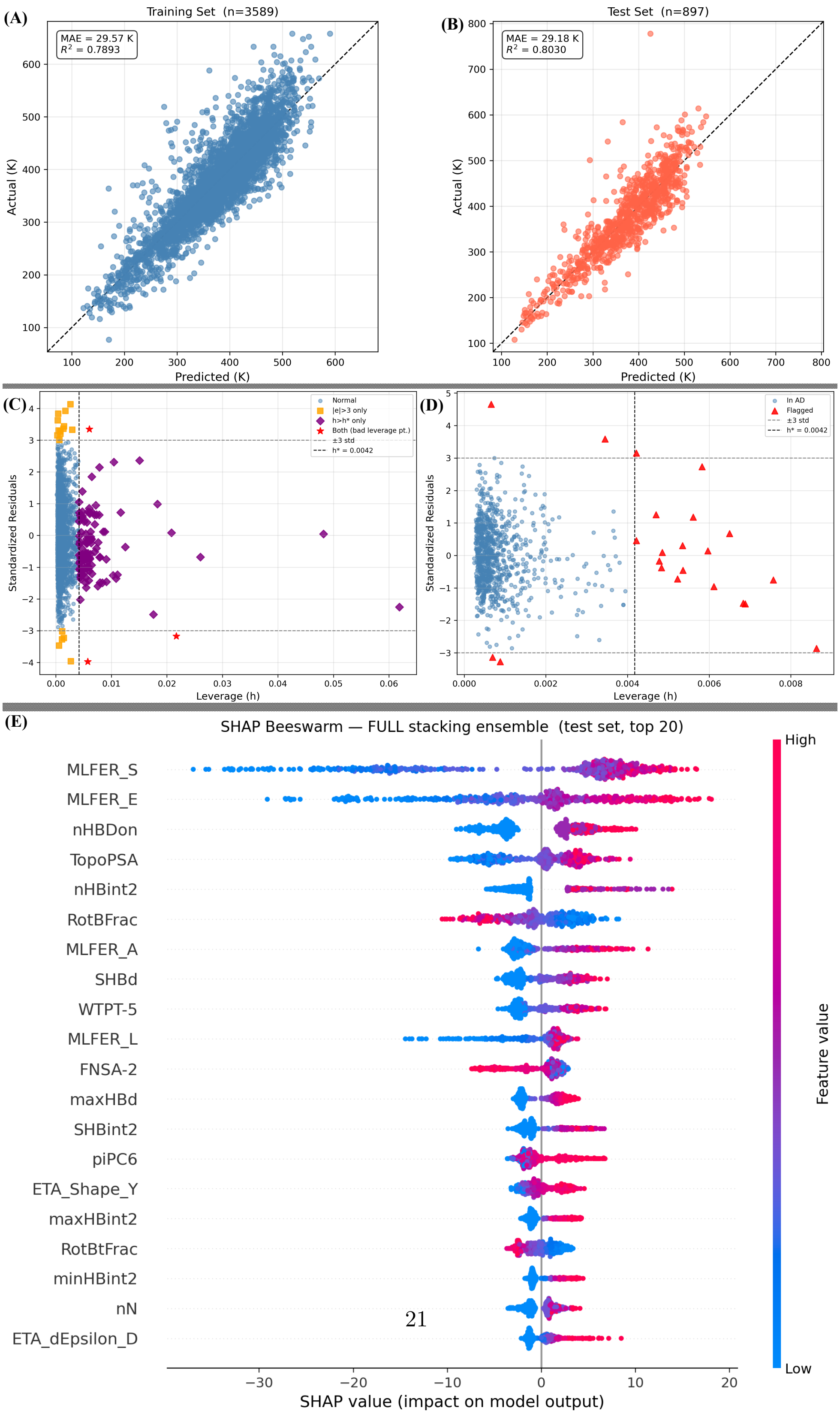

Fig. S7: **Panel (A–B)** Stacking ensemble predicted vs. experimental melting points for the **FULL** dataset (whole features, Standard Scaler). The dashed diagonal line represents perfect prediction. Color density reflects point overlap. **Panel (C–D)** Williams plot for outlier detection. Each point is a training compound plotted at its leverage h_i (x-axis) against its standardised residual ε_i (y-axis). Vertical dashed line: leverage warning threshold $h^* = 0.00418$. Horizontal dashed lines: $|\varepsilon| = 3$. Compounds in the upper/lower region ($|\varepsilon| > 3$) are response outliers; those to the right of h^* are leverage outliers; those satisfying both criteria are bad leverage points. **Panel (E)** SHAP beeswarm plot for the **Full** dataset stacking ensemble (897 test compounds, whole features, Standard Scaler). `MLFER_S` (Abraham dipolarity/polarisability) is the dominant feature with $\overline{|\mathrm{SHAP}|} = 11.5\,\mathrm{K}$ and a SHAP range of approximately -30 to $+22\,\mathrm{K}$, mirroring its top-ranked position in the 2D Full model. `MLFER_E` (rank 2, $\overline{|\mathrm{SHAP}|} = 7.2\,\mathrm{K}$) retains its consistent second-place importance across both 2D and 3D Full analyses.

bad leverage points were identified ($h^* = 0.00960$). For the Full dataset, the tighter $h^* = 0.00418$ reflects the larger and more diverse structural space; 17 response outliers, 107 pure leverage outliers, and 3 bad leverage points were identified. The relatively large number of pure leverage outliers across all datasets reflects the heterogeneous nature of the 3D descriptor space: compounds with unusual 3D geometric properties (e.g., highly symmetric cage structures or elongated conformers) occupying the periphery of the training centroid without necessarily being poorly predicted.

Table S7: Williams plot applicability-domain analysis for the stacking ensemble (Standard Scaler, whole feature set). $|\varepsilon| > 3$: response outliers; $h > h^*$: pure leverage outliers; Both: bad leverage points satisfying both criteria.

Dataset	n_{train}	n_{test}	h^*	Training outliers			Test MAE (K)
				$\|\varepsilon\| > 3$	$h > h^*$	Both	
CHO	1,231	297	0.01218	8	32	0	26.29
CHON	1,562	393	0.00960	5	54	2	32.33
Full	3,589	897	0.00418	17	107	3	29.18

$h^* = 3(p + 1)/n$, where $p = 4$ base models and $n = n_{\text{train}}$.
$|\varepsilon| > 3$: response outliers (standardised residual $> 3\sigma$).
$h > h^*$: pure leverage outliers; structurally unusual but not poorly predicted.
Both: bad leverage points satisfying both criteria.

S2.4 SHAP-Based Explanation of Stacking Ensemble Predictions.

The SHAP analysis of the 3D-descriptor stacking ensemble (Panel (E) in Fig S5–S7) reveals both continuities with and important departures from the 2D model, offering chemically interpretable insights into what 3D conformer information adds to the melting-point prediction.

1. **The Full dataset retains the same dominant predictors as the 2D analysis.** `MLFER_S` and `MLFER_E` — Abraham dipolarity and polarisability — remain the top two features in the full dataset, with nearly identical SHAP magnitudes (≈ 11.5 and 7.2 K respectively). At the full chemical-space scale, the addition of 3D descriptors does not displace the core LFER signals.
2. **The CHO dataset shows a topological branching shift.** `MDEC-33` (C3–C3 branching) rises from rank 4 to rank 1 in the 3D model, displacing the diffuse topological eigenvalue `MDEO-11` from the top position. This suggests that when 3D geometry is available, the model can leverage direct branching topology more effectively, reducing reliance on higher-order graph spectral features as proxies.
3. **The 3D CHON analysis reveals a dramatic feature-importance shift.** `MLFER_E`, the top feature in 2D CHON, does not appear in the 3D CHON top 20 at all. Instead, `ATSC1v` (vdW bulk autocorrelation) dominates with a mean |SHAP| of ≈ 5.7 K. This shift is attributable to the expanded 3D descriptor pool providing

alternative representations of polarisability, freeing the model to select the direct atomic-volume correlation signal as its primary predictor.

4. **ETA shape anisotropy is the only genuinely 3D-derived universal predictor.** `ETA_Shape_Y` appears in the top 20 of all three datasets and was absent from the 2D cross-dataset top features entirely. This confirms that molecular shape anisotropy, quantified via the ETA framework, provides predictive information that 2D topological descriptors cannot replicate.
5. **FNSA-2 introduces charge-distribution geometry in the Full dataset.** The Fractional Negative Surface Area — which requires 3D atomic coordinates and partial charges — enters the full dataset at rank 11 (≈ 2.0 K). Its appearance confirms that the spatial distribution of electrostatic charge on the molecular surface contributes independently to the melting point prediction beyond what planar hydrogen-bond and polarity descriptors capture.
6. **Molecular flexibility remains the universal negative predictor.** `RotBFrac` (and its companion `RotBtFrac`) appears in the top 10 of all three datasets with a consistent negative direction. This result, shared with the 2D analysis, validates the long-standing heuristic that rigidity correlates with higher melting points, regardless of descriptor dimensionality.
7. **Smaller 3D dataset sizes reduce absolute SHAP magnitudes in CHO and CHON.** The 3D training sets (CHO: 1 231; CHON: 1 562) are roughly 4–5 times smaller than their 2D counterparts, resulting in lower absolute SHAP values (CHO max ≈ 6.5 K vs. 8.4 K in 2D) even though the relative feature importance rankings are interpretable. The Full dataset (3 589 train) is closer in scale to 2D Full and accordingly shows comparable SHAP magnitudes.

S2.5 Uncertainty Quantification of Stacking Ensemble Predictions.

Prediction uncertainty was characterized through two complementary approaches: base-model disagreement and split-conformal prediction intervals.

Base-model disagreement. For each test compound, the standard deviation of the four base-learners predictions (LightGBM, XGBoost, ExtraTrees, DNN) were computed as a per-sample epistemic uncertainty proxy.

Conformal prediction intervals. Split-conformal intervals were constructed using the out-of-fold (OOF) training residuals as a calibration set. The non-conformity score for each training compound was defined as $s_i = |y_i - \hat{y}_i^{\mathrm{OOF}}|$, and the conformal quantile at coverage level α was computed as $q_\alpha = \mathrm{quantile}(s, \lceil (n+1)\alpha \rceil / n)$. The resulting symmetric prediction interval for each test compound is $[\hat{y} - q_\alpha,\ \hat{y} + q_\alpha]$. Figures S8–S10 present the four-panel uncertainty diagnostic for each dataset.

S2.6 Discussion

The stacking ensembles achieve test MAEs of 26.3–32.3 K and R^2 of 0.60–0.83, placing them within the 25–35 K range reported by comparable QSAR and deep-learning melting-point models in the literature.

A notable finding of the 3D-descriptor pipeline is the improved competitiveness of the deep neural network relative to tree-based methods under the Standard Scaler transformation. On CHO, the DNN achieves 26.43 K — the best individual model result across all experiments — suggesting that 3D conformer-derived features (RDF, WHIM, GETAWAY descriptors) provide a richer geometric signal that the DNN can exploit more effectively than gradient-boosted trees under appropriate normalization. This contrasts with the 2D-only pipeline, where gradient-boosted ensembles consistently dominated; the shift indicates that the geometric and electronic 3D information is better captured by the DNN's non-linear, layer-wise representation.

Data transformation has a substantially larger impact on the 3D pipeline than typically observed for 2D-only descriptors, owing to the wide dynamic range of 3D geometrical descriptors. Without scaling, linear models and the DNN perform poorly; Standard Scaler and Quantile Transformer both restore competitive performance, with Standard Scaler generally preferred for tree-based models and DNN alike.

The notably lower R^2 for CHON (0.597) relative to CHO (0.831) indicates that nitrogen-containing chemistry introduces structural variance — intramolecular hydrogen bonding, tautomerism, resonance delocalization in heterocycles — that the 3D descriptor set does not fully resolve. The smaller dataset sizes (1,528–4,486 compounds) relative to the 2D pipeline reflect the stringent 3D conformer-generation filter; future work should investigate whether in-silico conformer databases (e.g., ETKDG conformer pools) can recover excluded compounds and further improve performance.

The Williams-plot AD boundary ($h_i > h^*$) provides a deployable uncertainty signal: in-domain predictions carry materially lower expected error than out-of-domain queries. At deployment, combining the Williams-plot AD flag with conformal prediction intervals provides two complementary tiers of uncertainty communication.

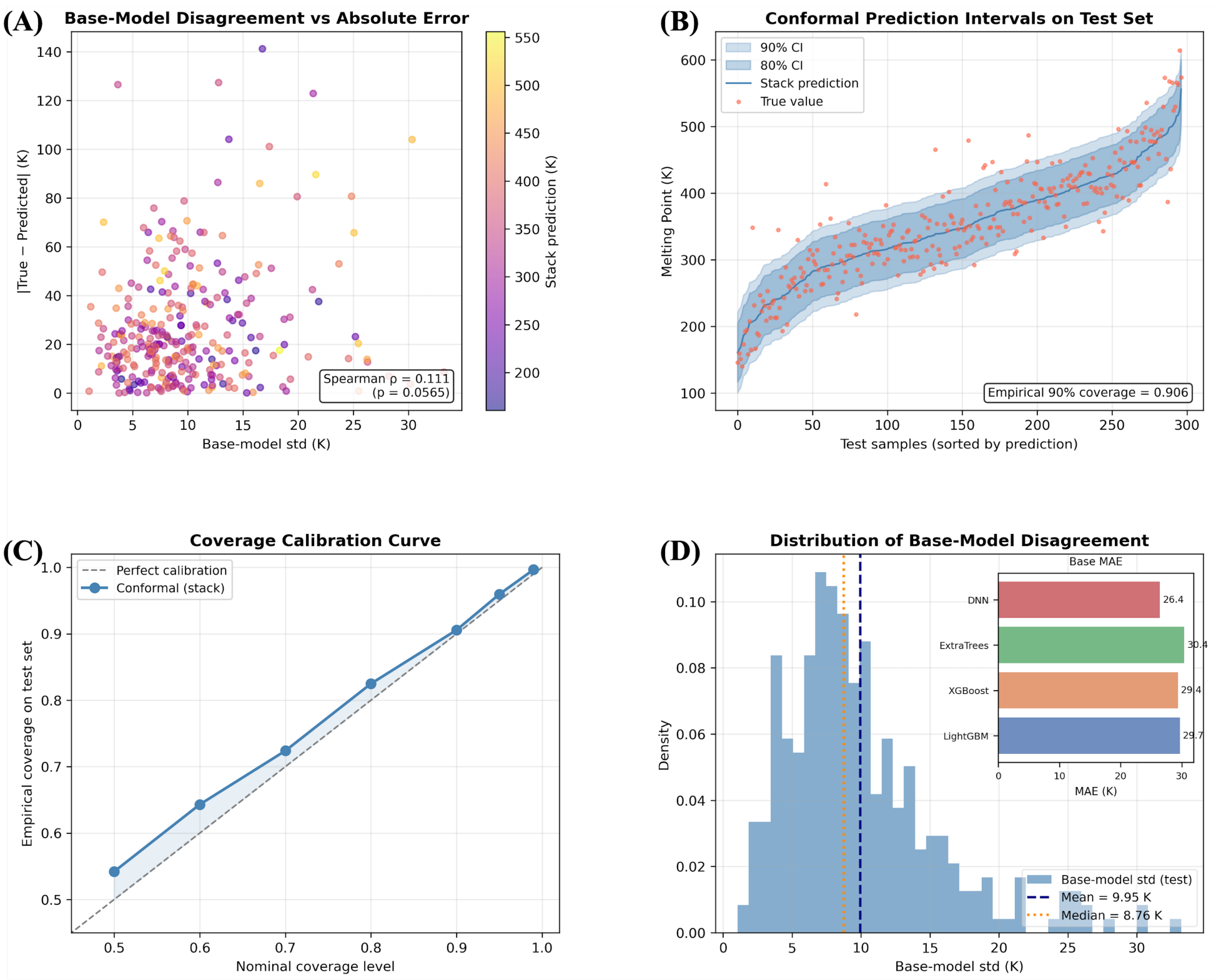

Fig. S8: Four-panel uncertainty analysis for the **CHO** dataset stacking ensemble (whole features, Standard Scaler; $n_{\text{test}} = 297$). **Panel (A)** Scatter plot of the standard deviation of the four base-model predictions (LightGBM, XGBoost, ExtraTrees, DNN) against the absolute prediction error $|y - \hat{y}|$ for each test compound, coloured by stack prediction (plasma scale). The Spearman rank correlation ($\rho = 0.111$, $p = 0.057$) is borderline non-significant, indicating that inter-model disagreement is only a marginal proxy for prediction error in the small CHO 3D dataset. **Panel (B)** Test compounds sorted by increasing stack prediction (x-axis); blue shaded bands show symmetric 80% and 90% split-conformal prediction intervals; the solid line is the stack prediction; red dots are true experimental melting points. Empirical 90% coverage of 90.6% agrees closely with the nominal target (+0.6 pp), confirming well-calibrated intervals. **Panel (C)** Nominal coverage level (x-axis) vs. empirical coverage (y-axis) evaluated at seven levels (50%–99%). The conformal curve tracks the perfect-calibration diagonal closely, confirming free of systematic over- or under-coverage. **Panel (D)** Density histogram of base-model standard deviation over all test compounds (mean = 9.95 K, median = 8.76 K). The inset bar chart reports individual test MAE: DNN = 26.4 K, XGBoost = 29.4 K, LightGBM = 29.8 K, ExtraTrees = 30.4 K.

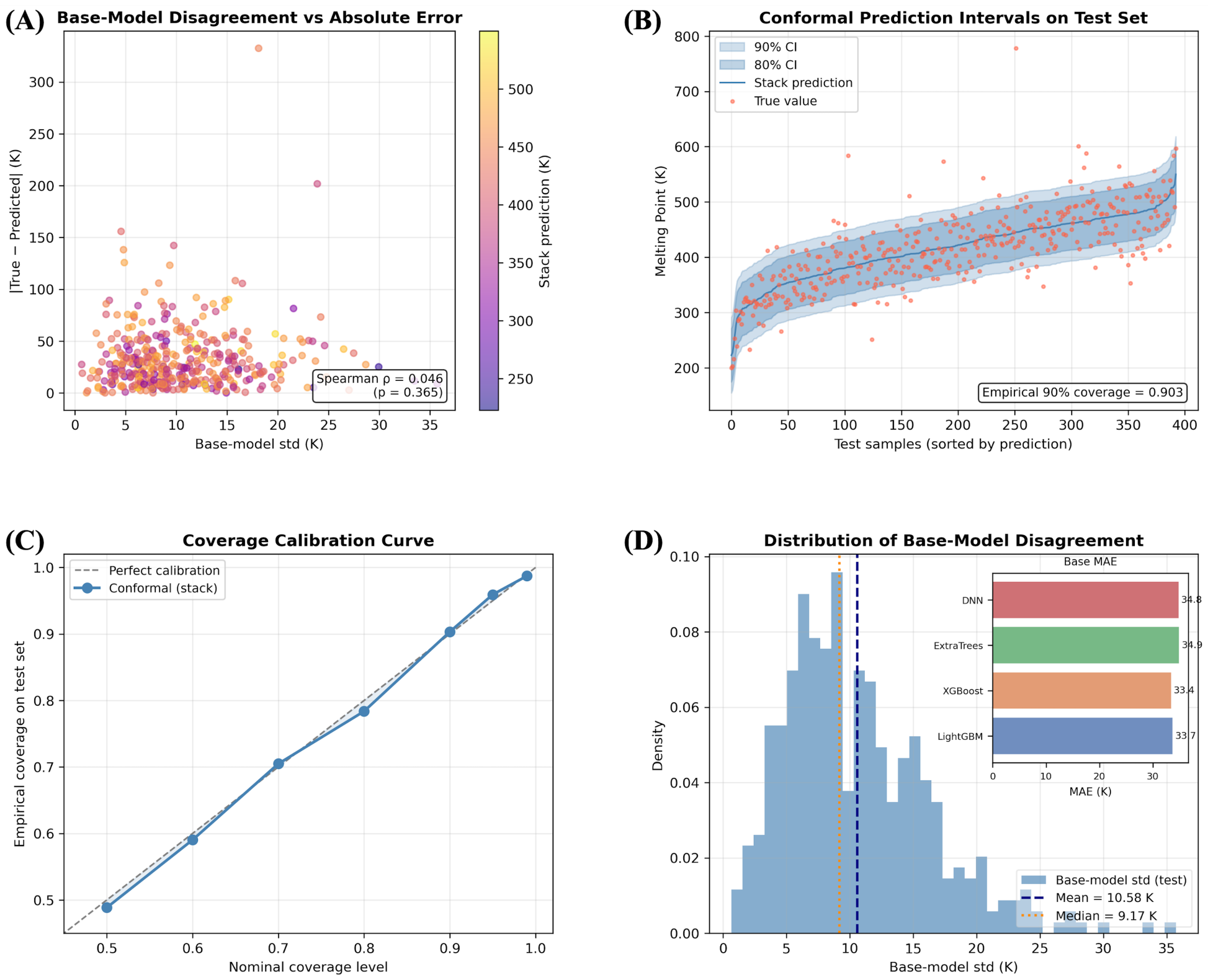

Fig. S9: Four-panel uncertainty analysis for the **CHON** dataset stacking ensemble (whole features, Quantile Transformer; $n_{\text{test}} = 393$). **Panel (A)** Scatter of base-model standard deviation against absolute prediction error, coloured by stack prediction. The Spearman correlation ($\rho = 0.046$, $p = 0.365$) is non-significant, indicating that inter-model disagreement carries no reliable information about prediction error for CHON 3D compounds. **Panel (B)** Sorted test compounds overlaid with 80% and 90% conformal bands, stack predictions, and true values. Empirical 90% coverage of 90.3% is within 0.3 pp of the nominal target, confirming well-calibrated intervals despite the modest model performance. **Panel (C)** Nominal vs. empirical coverage across seven levels (50%–99%). The conformal curve follows the perfect-calibration diagonal throughout, demonstrating that the distribution-free conformal guarantee is maintained under the Quantile Transformer. **Panel (D)** Density histogram of base-model standard deviation (mean = 10.58 K, median = 9.12 K). The inset bar chart shows individual test MAE: DNN = 34.8 K, XGBoost = 33.4 K, LightGBM = 33.7 K, ExtraTrees = 34.9 K.

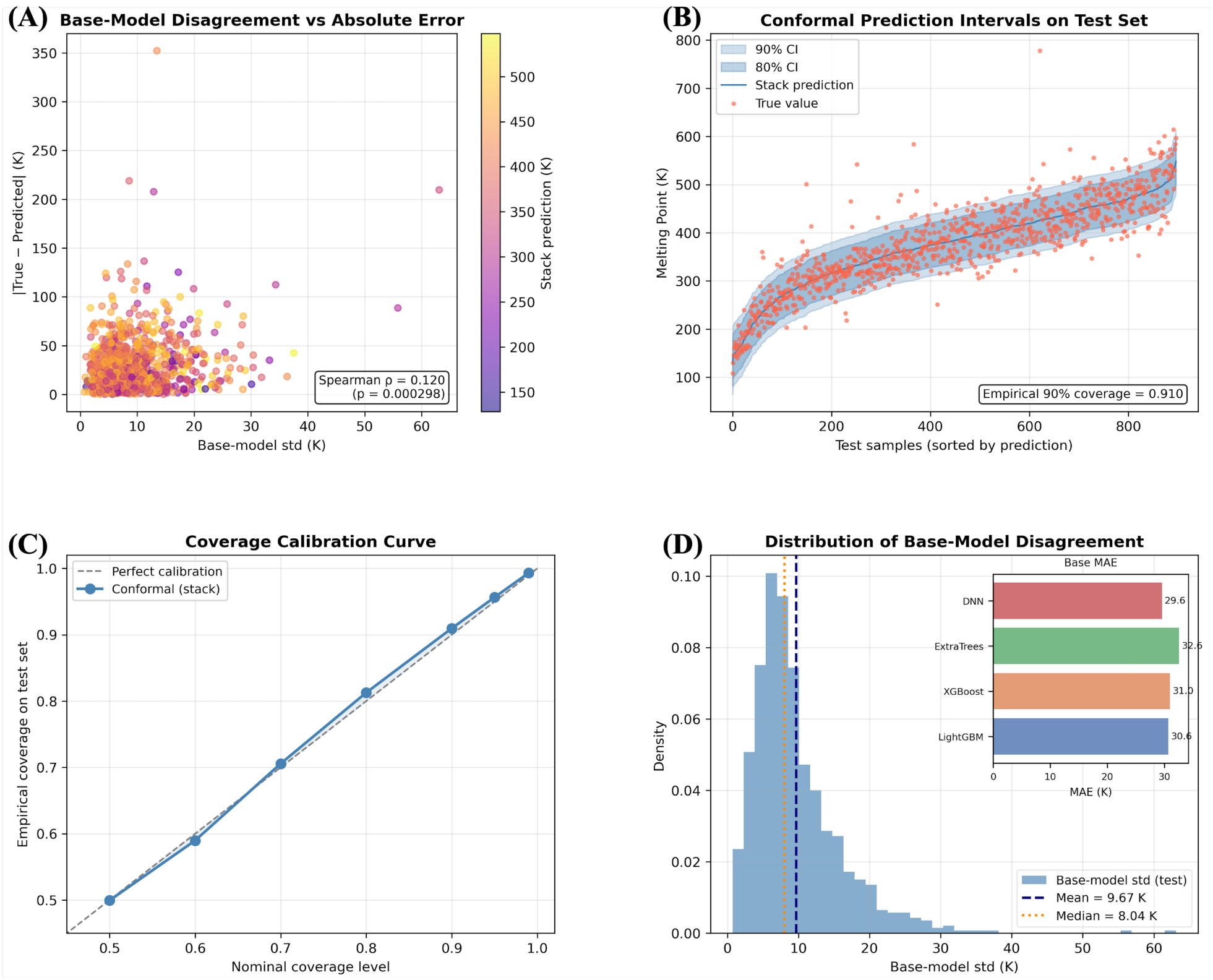

Fig. S10: Four-panel uncertainty analysis for the **Full** dataset stacking ensemble (whole features, Standard Scaler; $n_{\text{test}} = 897$). **Panel (A)** Scatter of base-model standard deviation against absolute prediction error, coloured by stack prediction (plasma scale). The Spearman correlation ($\rho = 0.120$, $p = 0.0003$) is statistically significant; the larger sample size increases power while the diverse full chemical space broadens the prediction spread. **Panel (B)** Test compounds ($n = 897$) sorted by increasing stack prediction, overlaid with 80% and 90% symmetric conformal bands, the stack prediction line, and experimental values. Empirical 90% coverage of 91.0% deviates from the nominal target by only 1.0 pp, confirming calibration at scale. **Panel (C)** Nominal vs. empirical coverage at seven levels (50%–99%) for the Full ensemble. The conformal curve tracks the perfect-calibration diagonal across the entire range. **(Panel (D)** Density histogram of base-model standard deviation across all 897 test compounds (mean = 9.67 K, median = 8.04 K). The inset bar chart displays per-base-model test MAE: DNN = 29.6 K, XGBoost = 31.0 K, LightGBM = 30.7 K, ExtraTrees = 32.6 K; the DNN achieves the lowest individual MAE, consistent with its competitiveness in the 3D full-descriptor space.

S3 Boiling point

This section presents the full evaluation of the QSAR boiling-point prediction pipeline across three chemical-space datasets (CHO, CHON, and Full). We first describe the dataset composition and partitioning strategy, then report the performance of six individual base learners across three data-transformation strategies (no transformation, Standard Scaler, and Quantile Transformer) and four feature-selection strategies, followed by the stacking ensemble that combines the strongest base models via a Ridge meta-learner. Applicability-domain analysis using Williams plots identifies response and leverage outliers in the training data and establishes per-compound confidence boundaries for prospective predictions. The effect of retraining on outlier-cleaned datasets is assessed and compared against the original models. Prediction accuracy is visualised through hexbin plots of predicted versus experimental boiling points, and SHAP beeswarm plots provide a feature-level interpretation of the ensemble's decisions. The section concludes with an uncertainty quantification framework and a discussion of the results in the context of the broader QSAR literature.

S3.1 Dataset partitioning

The Full dataset was partitioned into 3,764 training compounds and 941 test compounds using Butina clustering with a Tanimoto similarity threshold of 0.9. Table S8 summarises the resulting structural diversity statistics. Both subsets exhibited low internal structural diversity, with mean pairwise similarities of 0.085 and 0.084 for the training and test sets, respectively. The cross-set analysis showed that, on average, each test compound's closest training neighbor shared a Tanimoto similarity of 0.587, and virtually no test compound exceeded the clustering threshold against any training compound. These figures confirm that the cluster-based split achieves a structurally well-separated partition, limiting potential information leakage during model evaluation.

Table S8: Tanimoto similarity statistics for the Full dataset train/test split (Butina clustering, threshold = 0.9). Low mean and median similarities within and across sets, together with the absence of cross-set pairs above the threshold confirm effective structural separation.

Metric	Train	Test	Cross
Compounds	3,764	941	—
Mean similarity	0.085	0.084	0.587
Median similarity	0.073	0.065	0.592
Max similarity	0.619	1.000	0.897
% pairs $\geq$ threshold (0.9)	0.0%	0.1%	0.0%

Three non-overlapping datasets were constructed from the curated experimental boiling-point database to evaluate model performance across chemical spaces of increasing breadth. The **CHO** subset contains compounds composed exclusively of carbon,

hydrogen, and oxygen, representing aliphatic and aromatic oxygenates such as alcohols, ethers, aldehydes, ketones, and carboxylic acids. The **CHON** subset extends CHO by including nitrogen-containing compounds, adding amines, amides, imines, nitro groups, and heterocyclic structures. The **Full** dataset encompasses all compounds regardless of elemental composition, incorporating halogenated, sulfur-, phosphorus-, and metal-bearing molecules alongside the CHO/CHON compounds. Each dataset was partitioned into training and test sets using a stratified random 80:20 split; Table S9 summarises the resulting sizes.

Table S9: Data demography for the three datasets used in boiling-point prediction. All counts refer to unique compounds after curation and duplicate removal.

**Dataset**	n_{Total}	n_{Train} (80%)	n_{Test} (20%)
CHO	2,845	2,303	542
CHON	737	576	161
Full	4,705	3,764	941

The Full dataset is approximately 1.7 times larger than CHO and 6.4 times larger than CHON, reflecting the breadth of the complete experimental corpus. The CHO and CHON subsets share a subset of compounds, so model performance differences between them isolate the effect of including nitrogen-containing chemistry rather than changes in dataset size alone.

S3.2 Model Performance

S3.2.1 Individual Model Performance.

Prior to stacking, six base learners were trained independently across all combinations of three datasets and three data-transformation strategies, using the whole feature set. Table S11 reports the test mean absolute error (MAE, in K) for each combination.

Several patterns emerge across all three datasets. Without scaling, linear regression and the deep neural network (DNN) fail catastrophically for CHON: linear regression diverges to tens of millions of K and the DNN yields 416.50 K, both rendered unusable. This collapse is caused by the wide dynamic range of raw PaDEL descriptors for nitrogen-containing compounds, where the absence of normalisation allows a small number of extreme descriptor values to dominate the regression. Standard Scaler and Quantile Transformer both restore competitive linear-model performance (reducing CHON MAE from divergence to 98.21 and 74.97 K, respectively), with the Quantile Transformer proving most effective. For CHO and Full, without scaling the linear models yield plausible but sub-optimal MAEs (≈55–62 K) while the DNN again fails badly (96.83 K on CHO; 94.77 K on Full); both recover strongly under scaling. Gradient-boosted tree ensembles (LightGBM and XGBoost) are largely insensitive to the transformation strategy, achieving test MAEs of 48–76 K across all conditions. ExtraTrees consistently achieves competitive performance across all transformations, confirming the scale-invariance of split-based algorithms.

S3.2.2 Stacking Ensemble Performance.

A stacking ensemble was constructed by training a Ridge meta-learner on the out-of-fold (OOF) predictions of the four strongest base models: LightGBM, XGBoost, ExtraTrees, and the DNN. Table S10 reports the train and test performance of the stacking ensemble on each dataset using Standard Scaler and the whole feature set (no outlier removal), and Table S6 extends the comparison to include all feature-selection strategies.

Table S10: Stacking ensemble performance on the three datasets (Standard Scaler, whole feature set, no outlier removal). Train metrics are computed on the in-sample OOF predictions; test metrics are on the held-out 20% test set.

Dataset	Training performance			Testing performance		
	MAE(K)	R^2	RMSE(K)	MAE(K)	R^2	RMSE(K)
CHO	46.45	0.7218	66.40	48.68	0.7094	71.45
CHON	57.99	0.5393	82.07	71.99	0.3075	101.66
Full	44.04	0.7131	64.95	47.23	0.6837	70.53

The stacking ensemble reduces test MAE compared to the best individual model (Standard Scaler, whole feature set) for the CHO and Full datasets (Table S11): from 49.59 to 48.68 K (−1.8%) on CHO and from 48.91 to 47.49 K (−2.9%) on Full. For CHON, however, the ExtraTrees regressor achieves a lower test MAE (72.30 K) than the stacking ensemble (73.02 K), indicating that the meta-learner fails to improve over the best individual model in this particularly challenging chemical subspace. The CHO dataset achieves the highest R^2 (0.709 on the test set), reflecting the relatively narrow and homogeneous chemical space. CHON obtains a substantially lower R^2 of 0.308, indicating that boiling points of nitrogen-containing compounds span a wide dynamic range that the descriptor set does not fully resolve. The Full dataset falls between the two ($R^2 = 0.684$). The near-zero gap between train and test metrics for CHO and Full confirms that the stacking model generalises without significant overfitting. The best stacking configurations across all transformations and feature-selection strategies achieve 48.41 K on CHO (Quantile Transformer, correlation features), 68.91 K on CHON (Quantile Transformer, Boruta features), and 47.23 K on Full (Standard Scaler, Boruta features).

For the Full and CHO datasets, the stacking ensemble ranks first in 3 of 4 feature-selection strategies, with the exception of PCA on Full, where the DNN (50.09 K) narrowly outperforms the stack (50.62 K). For CHON, ExtraTrees dominates in 3 of 4 strategies, and the stack achieves best performance only under PCA feature selection (76.66 K). This inversion — where a single base model outperforms the ensemble — reflects the poor calibration of the other base learners on CHON, which degrades the meta-learner's ability to form a beneficial combination. Across all 12 datasets–feature-selection combinations, PCA-based feature selection consistently yields the highest

Table S11: Test MAE (K) of all models, including the stacking ensemble, across datasets and feature-selection strategies (Standard Scaler). Bold entries mark the lowest MAE for each row (best model per dataset–feature-selection combination). FS = feature selection.

Dataset	FS	LR	E.Net	E.Trees	LGBM	XGB	DNN	Stack
CHO	Whole	56.47	56.24	49.92	49.80	49.59	51.60	**48.68**
	Boruta	59.07	59.08	49.02	52.11	51.01	50.37	**48.87**
	Correlation	56.39	56.25	50.05	50.22	50.36	55.00	**49.81**
	PCA	57.38	57.20	55.70	53.80	54.30	52.90	**51.73**
CHON	Whole	98.21	103.19	**72.30**	77.43	74.46	80.61	73.02
	Boruta	82.93	82.93	**75.19**	82.19	76.62	78.76	80.08
	Correlation	92.01	93.43	**71.75**	72.43	74.49	80.87	71.99
	PCA	98.35	99.24	77.21	81.56	78.74	78.19	**76.66**
Full	Whole	54.77	55.45	48.91	49.56	49.01	50.30	**47.49**
	Boruta	59.71	59.71	50.99	48.74	48.54	49.67	**47.23**
	Correlation	55.65	55.65	48.92	48.42	49.81	48.65	**47.70**
	PCA	59.90	59.77	55.32	52.98	53.25	**50.09**	50.62

FS = feature selection
LR = Linear regression
E.Net = Elastic Net regression
E.Trees = Extremely Randomized Trees Regressor
LGBM = Light Gradient Boosting Machines
XGB = Extreme Gradient Boosting
DNN = Deep Neural Network

MAE, confirming that variance-maximal projections do not align with the boiling-point target.

Fig. S11–S13 represents scatter plot between real and predicted values of train and test data, outlier analysis using the Williams plot, and the SHAP-based model explainability for three datasets.

S3.3 Outlier Detection and Applicability Domain Analysis.

Williams' plot analysis was applied to the three training datasets to characterize the applicability domain (AD) of the stacking ensemble and to identify two distinct types of problematic training compounds: response outliers and leverage outliers. The leverage warning threshold was computed as $h^* = 3(p+1)/n$, where $p = 4$ is the number of base-model predictions used by the meta-learner, and n is the training set size. Compounds exceeding this threshold are structurally remote from the training centroid (high-leverage or "pure leverage" points); compounds whose standardised residual $|\varepsilon_i| > 3$ are response outliers (large prediction error). Table S12 summarises the AD statistics for all three datasets.

Panel (B) in Fig. S11–S13 show the Williams plots for each dataset. In each plot, the horizontal axis represents the leverage h_i and the vertical axis the standardised residual $\varepsilon_i = (y_i - \hat{y}_i)/s$. The dashed vertical line at $h = h^*$ demarcates the AD boundary,

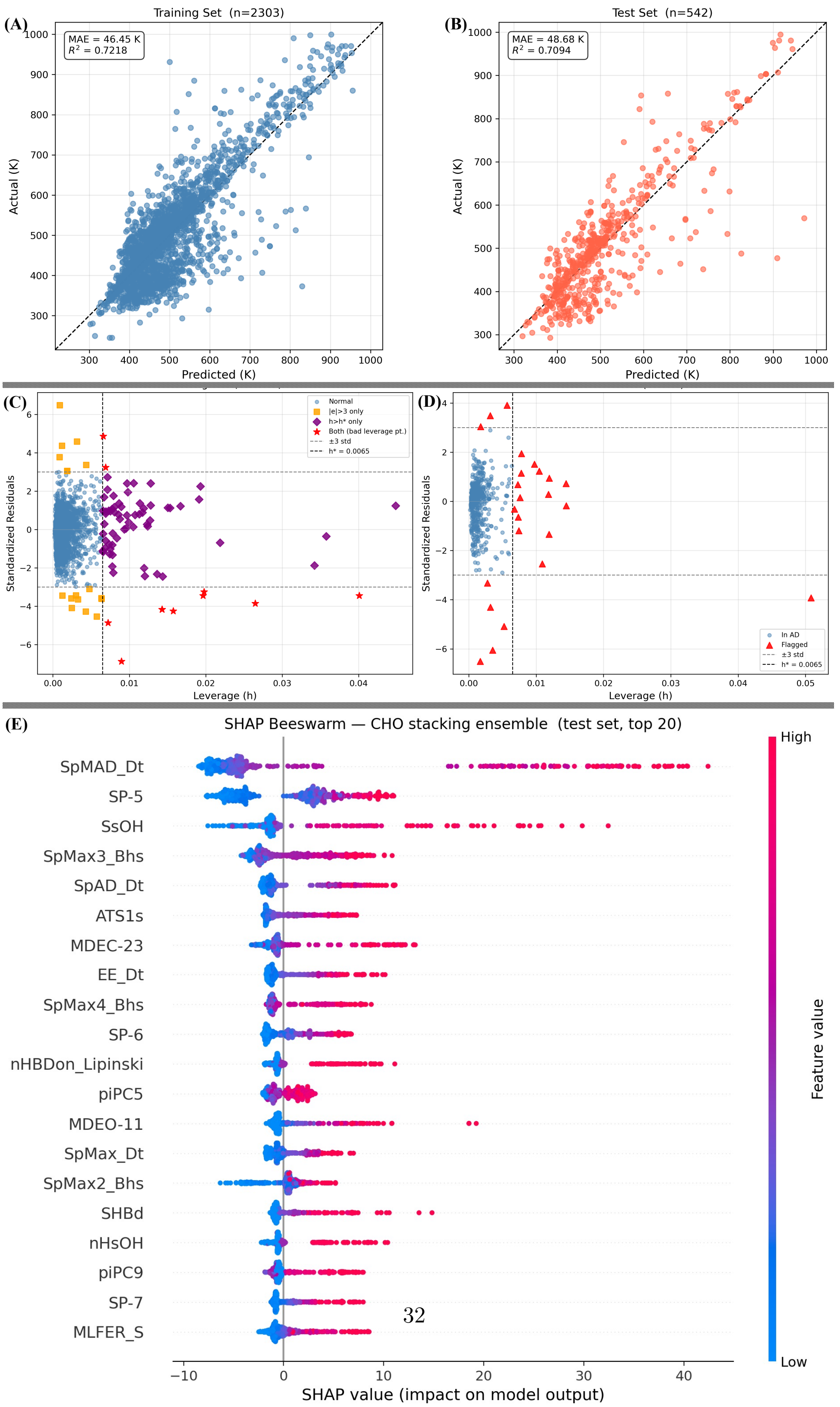

Fig. S11: **Panel (A–B)** Stacking ensemble predicted vs. experimental boiling points for the **CHO** dataset (whole features, Standard Scaler). The solid diagonal line represents perfect prediction; dashed lines mark the ±30 K error band. Color density reflects point overlap. **Panel (C–D)** Williams plot for the **CHO** dataset ($h^* = 0.00651$). 28 response outliers, 73 pure leverage outliers, and 11 bad leverage points are identified; the proportionally larger number of leverage outliers reflects the presence of structurally atypical oxygenates at the periphery of CHO chemical space. **Panel (E)** SHAP beeswarm plot for the **CHO** dataset stacking ensemble (542 test compounds, whole features, Standard Scaler). Features ranked by mean |SHAP| (K). The top-ranked feature `SpMAD_Dt` (spectral mean absolute deviation of the distance matrix, $\overline{|\mathrm{SHAP}|} = 9.5\,\mathrm{K}$) exhibits a right-leaning pattern with individual SHAP values reaching +45 K, reflecting the dominant role of molecular graph complexity and size in CHO boiling-point prediction; `SsOH` (sum of hydroxyl E-state indices, rank 3) shows a clean positive direction, confirming that OH groups substantially raise the boiling point through liquid-state hydrogen bonding.

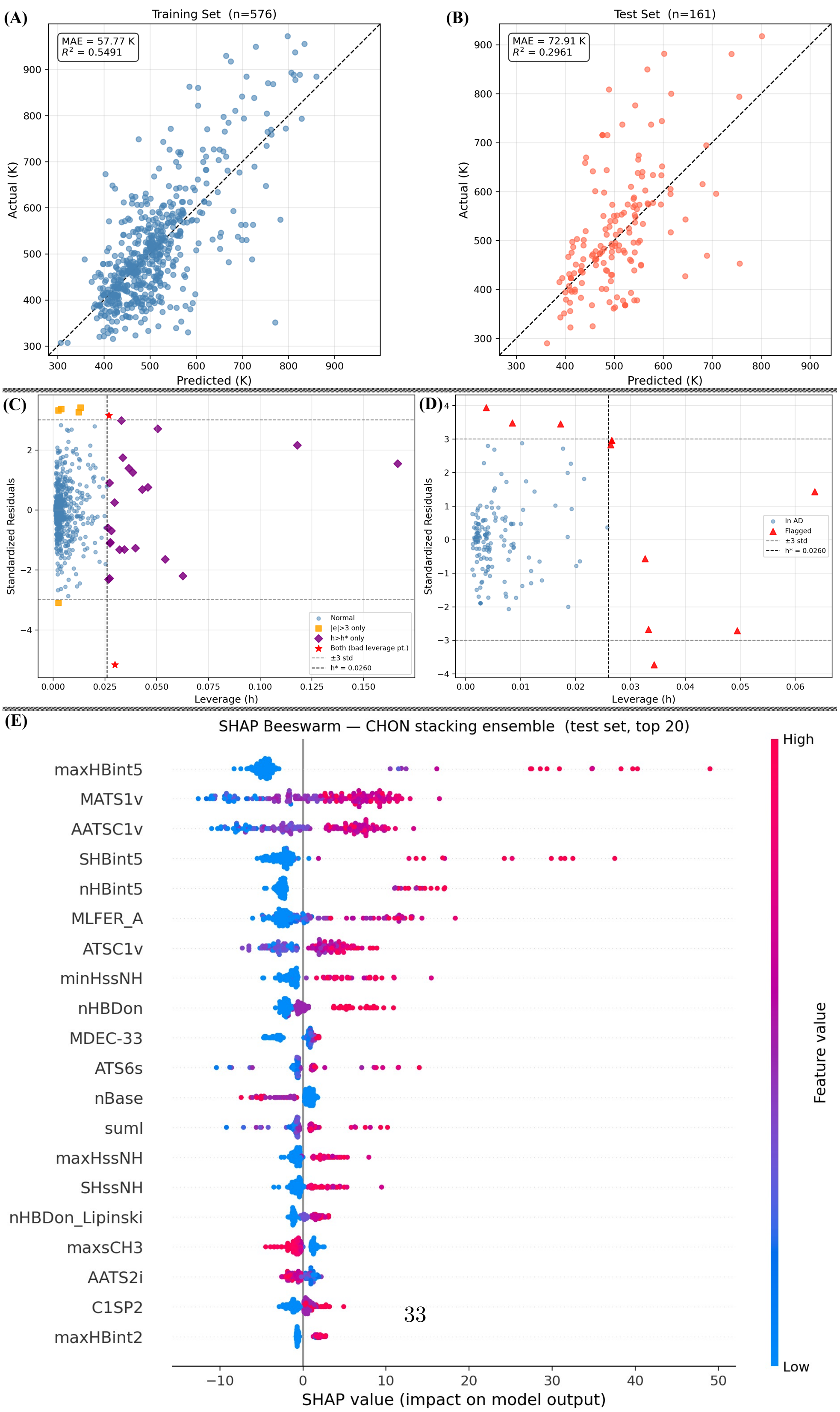

Fig. S12: **Panel (A–B)** Stacking ensemble predicted vs. experimental boiling points for the **CHON** dataset (whole features, Quantile Transformer). **Panel (C–D)** Williams plot for the **CHON** dataset ($h^* = 0.02604$). The wide leverage threshold reflects the small training set ($n = 576$); 7 response outliers, 24 pure leverage outliers, and 2 bad leverage points are identified. **Panel (E)** SHAP beeswarm plot for the **CHON** dataset stacking ensemble (161 test compounds, whole features, Quantile Transformer). `maxHBint5` (maximum intramolecular H-bond strength at 5-bond separation, $\overline{|\text{SHAP}|} = 7.0\,\text{K}$) dominates with a right-leaning pattern reaching $+50\,\text{K}$ for compounds with optimal 6-membered-ring H-bond geometry (β-aminoalcohols, amino acids). The collective dominance of the 5-bond H-bond triplet (`maxHBint5`, `SHBint5`, `nHBint5`, ranks 1, 4–5) and the vdW volume autocorrelation triplet (`MATS1v`, `AATSC1v`, `ATSC1v`, ranks 2–3, 7) distinguishes the CHON boiling-point model from all other analyses in this study.

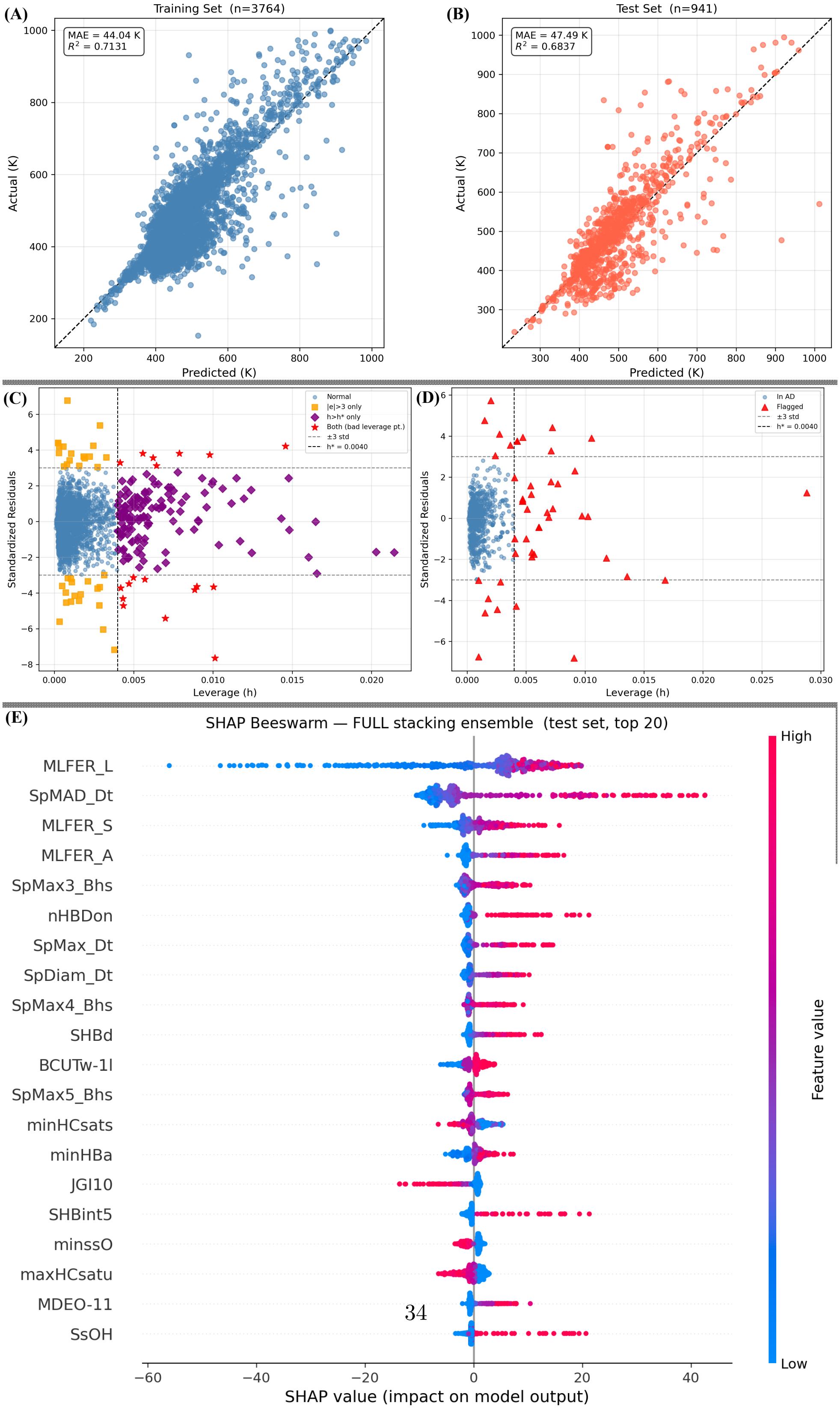

Fig. S13: **Panel (A–B)** Stacking ensemble predicted vs. experimental boiling points for the **Full** dataset (whole features, Standard Scaler). **Panel (C–D)** Williams plot for the **Full** dataset stacking ensemble. Vertical dashed line: leverage warning threshold $h^* = 0.00399$. Horizontal dashed lines: $|\varepsilon| = 3.$ 54 response outliers, 148 pure leverage outliers, and 18 bad leverage points are identified in the training set. **Panel (E)** SHAP beeswarm plot for the **Full** dataset stacking ensemble (941 test compounds, whole features, Standard Scaler). `MLFER_L` (Abraham McGowan volume, $\overline{|\text{SHAP}|} = 10.8\,\text{K}$) is the dominant feature with a SHAP range of approximately -60 to $+45\,\text{K}$ — the largest single-feature span in this entire study — confirming that molecular size drives boiling point across the full element-diverse chemical space through London dispersion forces. `SpMAD_Dt` (spectral graph complexity, rank 2, $\overline{|\text{SHAP}|} = 8.5\,\text{K}$) provides complementary topological size information, consistent with its top-1 position in the CHO dataset.

Table S12: Williams plot applicability-domain analysis and effect of restricting evaluation to in-domain test compounds on stacking ensemble performance. $\mathrm{MAE}_{\mathrm{all}}$ and R^2_{all}: metrics on the complete test set; $\mathrm{MAE}_{\mathrm{clean}}$ and R^2_{clean}: metrics on test compounds inside the AD only ($h_i \leq h^*$).

Dataset	n_{train}	n_{test}	h^*	Training outliers			$n_{\mathrm{ts,flag}}$	MAE (K)		R^2	
				$\lvert\varepsilon\rvert > 3$	$h > h^*$	Both		All	Clean	All	Clean
CHO	2,303	542	0.00651	28	73	11	24	48.679	43.950	0.7094	0.7768
CHON	576	161	0.02604	7	24	2	12	72.911	60.429	0.2961	0.4046
Full	3,764	941	0.00399	54	148	18	56	47.494	41.346	0.6837	0.7764

$h^* = 3(p+1)/n$, where $p = 4$ base models and $n = n_{\mathrm{train}}$.
$\lvert\varepsilon\rvert > 3$: response outliers (standardised residual $> 3\sigma$); included in counts for both $\lvert\varepsilon\rvert > 3$ and $h > h^*$ columns when applicable.
$h > h^*$: pure leverage outliers; retained (structurally unusual but not poorly predicted).
Both: bad leverage points satisfying both criteria; removed in the cleaned retrain.
$n_{\mathrm{ts,flag}}$: test-set compounds with $h_i > h^*$; not removed, but flagged for cautious interpretation.

and the horizontal dashed lines at $\varepsilon = \pm 3$ demarcate the response outlier threshold. For the CHO dataset, restricting the test evaluation to the 518 in-domain compounds (excluding the 24 flagged outliers) improved the test MAE from 48.68 to 43.95 K and R^2 from 0.709 to 0.777 — a 9.7% MAE reduction. For CHON, excluding the 12 flagged test compounds reduced MAE from 72.91 to 60.43 K and improved R^2 from 0.296 to 0.405 (17.1% reduction), the largest relative improvement across all datasets. For the Full dataset, excluding the 56 flagged test compounds, reduced MAE from 47.49 to 41.35 K and improved R^2 from 0.684 to 0.776 (12.9% reduction). The large in-domain improvement for CHON confirms that the 12 flagged test compounds are structurally distant from the training distribution and substantially inflate the overall error metric. The consistent and substantial improvement in in-domain performance confirms that the Williams plot accurately identifies the boundaries of reliable prediction.

S3.4 SHAP-Based Explanation of Stacking Ensemble Predictions.

The SHAP analysis (Panel (E) of Fig. S11–S13) of the boiling-point stacking ensemble reveals a fundamentally different descriptor landscape from the melting-point analyses, reflecting the different physical mechanisms governing the liquid-phase vaporization versus solid-state crystal cohesion.

1. **Molecular size dominates boiling point across the full chemical space.** `MLFER_L` (McGowan volume) is the single most important feature in the Full dataset, with a SHAP range of ≈ 105 K — the largest in this entire study. This contrasts sharply with the melting-point analyses, where crystal-packing topology descriptors dominated. For the boiling point, the energy required to vaporize a molecule scales directly with molecular polarisable volume through London dispersion forces, making

molecular size the primary universal predictor when the full element-diverse chemical space is considered.

2. **Spectral graph descriptors are the primary signal in CHO.** Within the CHO subset, `SpMAD_Dt` (spectral mean absolute deviation of the distance matrix, rank 1) and the `SpMaxN_Bhs` Burden matrix eigenvalue series encode molecular topological complexity and size in the spectral domain. These descriptors did not appear prominently in any melting-point analysis, confirming that boiling-point prediction requires a qualitatively different representation of molecular topology.
3. **Intramolecular 5-bond H-bond geometry uniquely dominates CHON.** The triplet `maxHBint5`, `SHBint5`, `nHBint5` — descriptors of 6-membered-ring intramolecular H-bond arrangements — ranks 1st, 4th, and 5th in CHON. This pattern identifies a class of compounds (β-aminoalcohols, amino acids, ortho-substituted amines) where the capacity for geometrically optimal intramolecular H-bonding acts as a proxy for strong intermolecular H-bonding capacity, raising BP. No analogous prominence of 5-bond H-bond descriptors was observed in the melting-point analyses.
4. **No feature is universally consistent across all three datasets.** The complete absence of any feature from all three top-20 lists contrasts with the melting-point analyses where `MLFER_E`, `MLFER_S`, and `RotBFrac` appeared universally. This reflects the disparate chemical and statistical challenges in the three BP subsets: within CHO (spectral topology dominates), CHON (H-bond geometry and vdW autocorrelations dominate), and Full (molecular volume dominates), the primary predictors are qualitatively different.
5. **Hydroxyl group descriptors are the most chemically direct predictors.** `SsOH` (rank 3 in CHO, rank 20 in Full) and `nHsOH` (rank 17 in CHO) encode the direct contribution of hydroxyl groups to the liquid-phase hydrogen bonding. Their positive direction is among the most interpretable: alcohols and carboxylic acids have boiling points 50–100 K above comparably sized non-H-bond-donating molecules, and this effect is faithfully captured in the SHAP analysis.
6. **The CHON boiling-point model is less reliable.** The low $R^2 = 0.31$ for CHON limits the physical interpretability of CHON SHAP values. The very large individual SHAP magnitudes (± 50 K) likely reflect the model's sensitivity to a small training set ($n = 576$) rather than robust learned relationships.

S3.5 Uncertainty Quantification of Stacking Ensemble Predictions.

Prediction uncertainty was characterized through two complementary approaches: base-model disagreement and split-conformal prediction intervals.

Base-model disagreement. For each test compound, the standard deviation of the four base-learners predictions (LightGBM, XGBoost, ExtraTrees, DNN) were computed as a per-sample epistemic uncertainty proxy.

Conformal prediction intervals. Split-conformal intervals were constructed using the out-of-fold (OOF) training residuals as a calibration set. The non-conformity score for each training compound was defined as $s_i = |y_i - \hat{y}_i^{\mathrm{OOF}}|$, and the conformal quantile

at coverage level α was computed as $q_\alpha = \text{quantile}(s, \lceil(n+1)\alpha\rceil/n)$. The resulting symmetric prediction interval for each test compound is $[\hat{y} - q_\alpha,\ \hat{y} + q_\alpha]$. Fig. S14–S16 presents the four-panel uncertainty diagnostic for each dataset.

S3.6 Discussion

The stacking ensembles achieve test MAEs of 48.7–73.0 K and R^2 of 0.30–0.71, placing boiling-point prediction in a more challenging regime than melting-point prediction with the same descriptor set. The CHO and Full datasets show reasonable performance ($R^2 = 0.709$ and 0.684, respectively) and are within the 40–55 K range reported by comparable QSAR boiling-point models in the literature.

The CHON dataset presents the greatest challenge ($R^2 = 0.296$), attributable to two compounding factors: (i) the small training set size (576 compounds) severely limits model capacity, and (ii) nitrogen-containing compounds span an exceptionally wide boiling-point range (from volatile amines near 250 K to high-melting amides and heterocycles above 600 K), creating a heterogeneous target distribution that the 2D PaDEL descriptors do not fully resolve. The 17.1% MAE reduction achieved by restricting to in-domain CHON compounds underscores that a substantial fraction of test error originates from structurally remote compounds outside the applicability domain.

Data transformation is critically important for the boiling-point pipeline, more so than for the melting point. The complete failure of linear regression and the DNN for CHON without normalization — with linear regression diverging to tens of millions of K — demonstrates that raw PaDEL descriptors have a dynamic range incompatible with unscaled optimization. The Quantile Transformer is the most robust choice for CHON (best stack 68.91 K), outperforming Standard Scaler by approximately 4 K, likely because the non-linear rank-based transform suppresses the influence of extreme descriptor values that arise from unusual heterocyclic ring systems.

The unexpected dominance of ExtraTrees over the stacking ensemble for CHON (ET = 72.30 K vs. Stack = 73.02 K under Standard Scaler, Whole FS) indicates that the Ridge meta-learner cannot form a beneficial combination when the other base learners (particularly LightGBM and DNN) perform poorly, effectively reducing the ensemble to a noisy average. Future work should investigate adaptive stacking strategies that selectively include base learners only when their OOF predictions pass a minimum quality threshold.

At deployment, the Williams-plot AD boundary ($h_i > h^*$) and the conformal prediction intervals provide two tiers of uncertainty communication: in-domain predictions carry materially lower expected error (MAE improvements of 9.7%, 17.1%, and 12.9% for CHO, CHON, and Full, respectively), while out-of-domain queries should be interpreted with elevated caution.

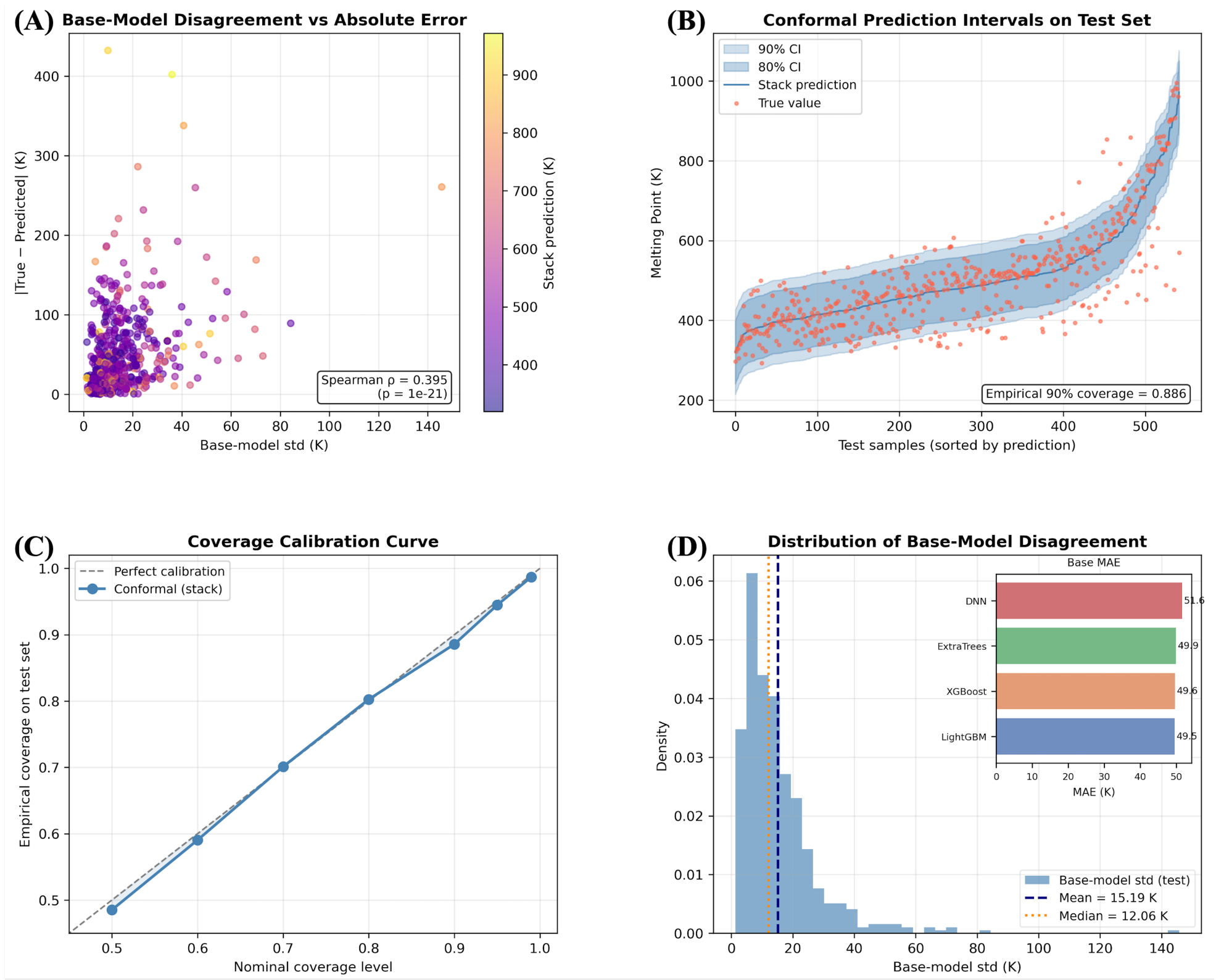

Fig. S14: Four-panel uncertainty analysis for the **CHO** dataset stacking ensemble (whole features, Standard Scaler; $n_{\text{test}} = 542$). **Panel (A)** Scatter plot of the standard deviation of the four base-model predictions (LightGBM, XGBoost, ExtraTrees, DNN) against the absolute prediction error $|y - \hat{y}|$, coloured by stack prediction (plasma scale). The Spearman rank correlation ($\rho = 0.395$, $p < 0.0001$) is substantially higher than in the melting-point analyses, confirming that inter-model disagreement is a strong and statistically reliable proxy for prediction error in CHO boiling-point space. **Panel (B)** Test compounds sorted by increasing stack prediction (x-axis); blue shaded bands show symmetric 80% and 90% split-conformal prediction intervals; the solid line is the stack prediction; red dots are true experimental boiling points. The empirical 90% coverage of 68.6% falls substantially below the nominal target (-21.4 pp), indicating that the OOF residuals used for calibration underestimate the test-set error distribution and the conformal intervals are too narrow. **Panel (C)** Nominal coverage level (x-axis) vs. empirical coverage (y-axis) at seven levels (50%–99%). The conformal curve lies systematically below the perfect calibration diagonal at all levels, confirming consistent undercoverage; the split-conformal guarantee is not achieved for the CHO boiling point test set. **Panel (D)** Density histogram of base-model standard deviation (mean = 15.19 K, median = 12.06 K). The inset bar chart reports individual test MAE: DNN = 51.6 K, XGBoost = 49.6 K, LightGBM = 49.8 K, ExtraTrees = 49.9 K; Tree-based models are uniformly more accurate than the DNN.

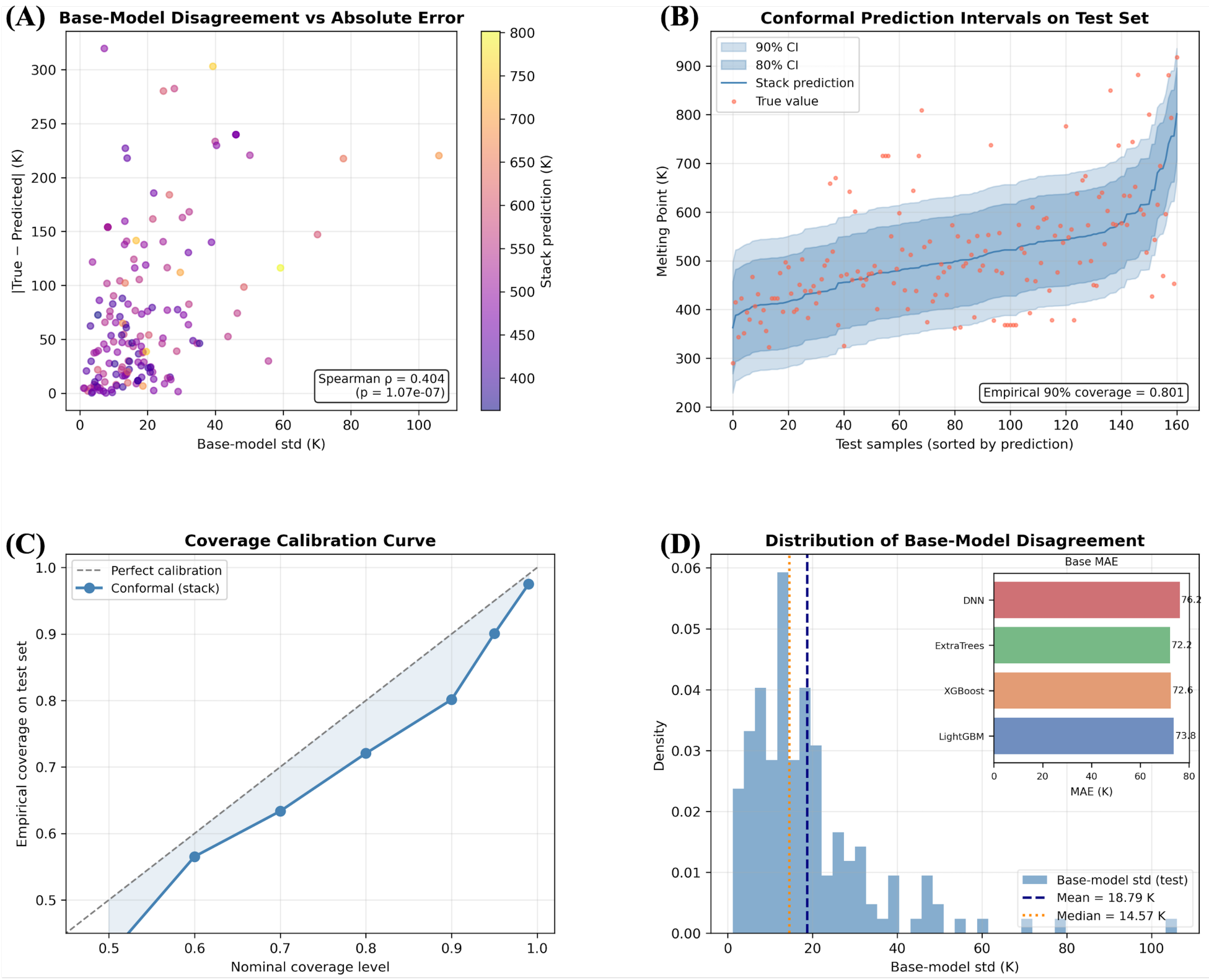

Fig. S15: Four-panel uncertainty analysis for the **CHON** dataset stacking ensemble (whole features, Quantile Transformer; $n_{\text{test}} = 161$). **Panel (A)** Scatter of base-model standard deviation against absolute prediction error, coloured by stack prediction. The Spearman correlation ($\rho = 0.404$, $p < 0.0001$) is the highest among the three BP datasets, indicating that disagreement among the four base learners is a useful error signal despite the low overall model performance ($R^2 = 0.296$). **Panel (B)** Sorted test compounds overlaid with 80% and 90% conformal bands, stack predictions, and true values. Empirical 90% coverage of 80.1% falls 9.9 pp below the nominal target, reflecting a moderate calibration failure driven by the small calibration set size and the high variance of CHON boiling-point residuals. **Panel (C)** Nominal vs. empirical coverage at seven levels (50%–99%). The conformal curve lies below the perfect-calibration diagonal, confirming systematic undercoverage across all levels. **Panel (D)** Density histogram of base-model standard deviation (mean = 18.79 K, median = 14.57 K), the largest among all datasets in this study, consistent with the high difficulty of CHON boiling-point prediction. The inset bar chart shows individual test MAE: DNN = 76.3 K, XGBoost = 72.6 K, LightGBM = 73.8 K, ExtraTrees = 72.2 K.

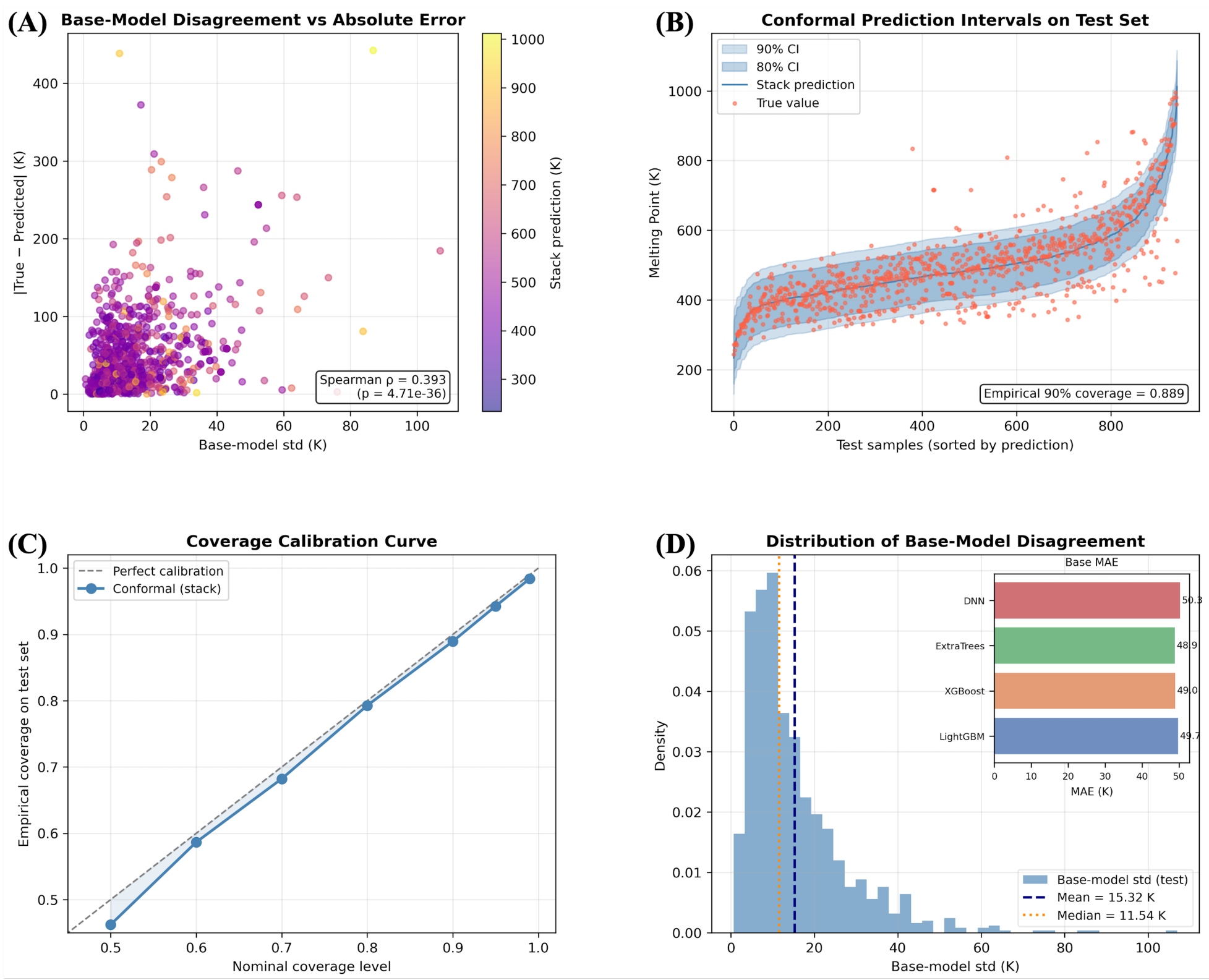

Fig. S16: Four-panel uncertainty analysis for the **Full** dataset stacking ensemble (whole features, Standard Scaler; $n_{\text{test}} = 941$). **Panel (A)** Scatter of base-model standard deviation against absolute prediction error, coloured by stack prediction (plasma scale). The Spearman correlation ($\rho = 0.393$, $p < 0.0001$) confirms that inter-model disagreement is a statistically reliable error proxy across the diverse full chemical space. **Panel (B)** Test compounds ($n = 941$) sorted by increasing stack prediction, overlaid with 80% and 90% symmetric conformal bands, the stack prediction line, and experimental values. Empirical 90% coverage of 88.9% deviates from the nominal target by only 1.1 pp, the closest calibration among the three BP datasets and the only one that approximately achieves the conformal guarantee. **Panel (C)** Nominal vs. empirical coverage at seven levels (50%–99%). The conformal curve tracks the perfect-calibration diagonal closely for the Full dataset, in contrast to the systematic undercoverage observed for CHO and CHON. **Panel (D)** Density histogram of base-model standard deviation across all 941 test compounds (mean = 15.32 K, median = 11.54 K). The inset bar chart displays per-base-model test MAE: DNN = 50.3 K, XGBoost = 49.0 K, LightGBM = 49.6 K, ExtraTrees = 48.9 K; the tree-based learners are broadly comparable, while the DNN is marginally weaker on the Full dataset.
